# β-Amino- and Alkoxy-Substituted Disilanides

**DOI:** 10.3390/molecules24213823

**Published:** 2019-10-23

**Authors:** Istvan Balatoni, Johann Hlina, Rainer Zitz, Alexander Pöcheim, Judith Baumgartner, Christoph Marschner

**Affiliations:** Institut für Anorganische Chemie, Technische Universität Graz, Stremayrgasse 9, A-8010 Graz, Austria; istvan.balatoni@yahoo.com (I.B.); johann.hlina@tugraz.at (J.H.); Rainer_Zitz@hotmail.com (R.Z.); alexander.poecheim@tugraz.at (A.P.)

**Keywords:** silanide, disilene adduct, germanide

## Abstract

Our recent study on formal halide adducts of disilenes led to the investigation of the synthesis and properties of β-fluoro- and chlorodisilanides. The reaction of the functionalized neopentasilanes (Me_3_Si)_3_SiSiPh_2_NEt_2_ and (Me_3_Si)_3_SiSiMe_2_OMe with KO*^t^*Bu in the presence of 18-crown-6 provided access to structurally related β-alkoxy- and amino-substituted disilanides. The obtained Et_2_NPh_2_Si(Me_3_Si)_2_SiK·18-crown-6 was converted to a magnesium silanide and further on to Et_2_NPh_2_Si(Me_3_Si)_2_Si-substituted ziroconocene and hafnocene chlorides. In addition, an example of a silanide containing both Et_2_NPh_2_Si and FPh_2_Si groups was prepared with moderate selectivity. Also, the analogous germanide Et_2_NPh_2_Si(Me_3_Si)_2_GeK·18-crown-6 could be obtained.

## 1. Introduction

The development of silanide chemistry [1,2,3,4,5] has advanced the progress of organosilicon chemistry over the last decades. Being the silicon analogs of carbanions, these compounds are invaluable for the construction of Si-X bonds. Especially, the formation of Si-Si bonds, which has long been restricted to Wurtz-type coupling of halosilanes, has been facilitated by the use of silanides, which allow much easier access to inherently asymmetric di- and oligosilanes [6]. In addition, these compounds also allow a much more flexible access to transition-metal silyl complexes and silylated organic compounds.

Typically, silanides are highly reactive species which are easily hydrolyzed to hydrosilanes. They need to be prepared, stored, and handled under strict exclusion of moisture. Initially, the chemistry of silanides was mainly restricted to alkyl-, aryl-, and silyl-substituted compounds. The introduction of functional groups to silanides represented a logical advance to allow further transformations of the formed di- and oligosilanes. Examples of hydrogen-substituted silanides date back to Gilman’s cleavage of HPh_2_SiSiPh_2_H with lithium [7], and related compounds have been used also later [8,9]. The attachment of Lewis basic heteroatoms to silanides was pioneered by Kawachi and Tamao, who introduced α-aminosilanides in the early 1990s [10,11] and could demonstrate their use as hydroxy anion equivalent in organic synthesis [11]. Later on, α-alkoxy- [12,13,14] and fluorosilanides [15,16,17] were introduced, and these compounds (silylenoids) turned out to be ambiphilic with a tendency to self-condensation. This was not observed for aminosilanides, although by NMR spectroscopy they clearly showed a relationship to those more reactive compounds [18].

Recently, we have reported a study on β-halodisilanides XPh_2_SiSi(SiMe_3_)_2_K (X = F, Cl) [19], which we not obtained by self-condensation of halosilylenoids but by reaction of XPh_2_SiSi(SiMe_3_)_3_ with potassium *tert*-butoxide. These compounds were found to possess distinctly different properties compared to the self-condensation product F(Me_3_Si)_2_SiSi(SiMe_3_)_2_K [15,16].

## 2. Results and Discussion

**Figure 1:**
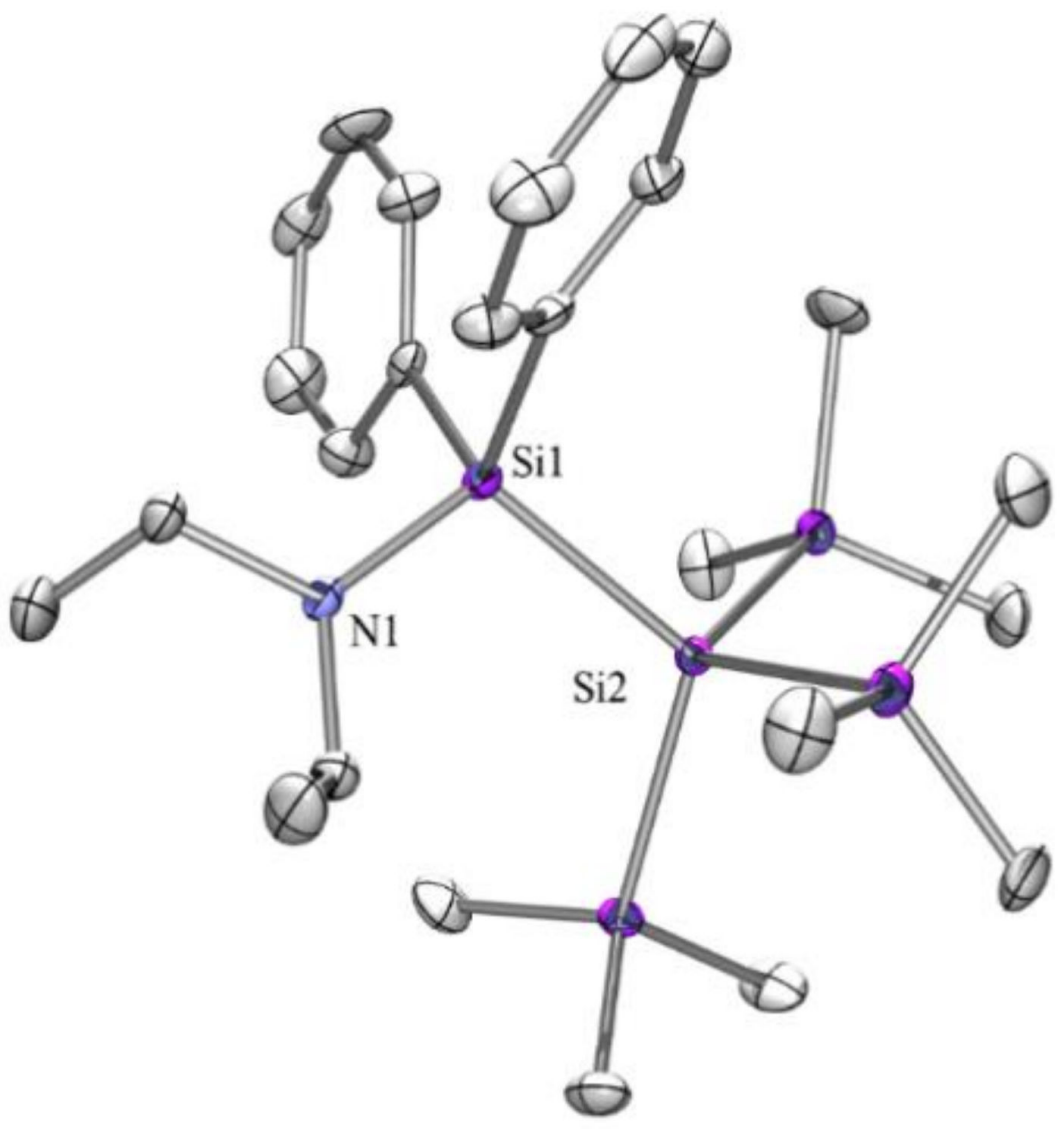
Molecular structure of compound **1** (thermal ellipsoid plot drawn at the 30% probability level). All hydrogen atoms are omitted for clarity (bond lengths in Å, angles in degrees). Si(1)-N(1) 1.717(3), Si(1)-C(1) 1.903(4), Si(1)-Si(2) 2.3744(14), Si(2)-Si(3) 2.3540(15), Si(2)-Si(4) 2.3657(15), Si(2)-Si(5) 2.3706(14), Si(3)-C(18) 1.870(5), N(1)-C(15) 1.458(5), N(1)-Si(1)-Si(2) 110.76(11), Si(3)-Si(2)-Si(4) 107.04(6).

After investigating the chemistry of α-aminooligosilanides some time ago [20], we now wish to extend our studies to β-aminooligodisilanides, which can be considered the formal condensation products of α-aminooligosilanides. As the latter do not undergo facile dimerization, a straightforward way to obtain β-aminooligodisilanides is the reaction of aminosilylchlorides with oligosilanides followed by further silanide formation via a reaction with potassium *tert*-butoxide. The synthesis of (Me_3_Si)_3_SiSiPh_2_NEt_2_ (**1**) was thus achieved by reaction of (Me_3_Si)_3_SiK [21,22] with Et_2_NPh_2_SiCl [23,24]. Single-crystal XRD analysis of the obtained compound (Figure 1) showed that it crystallized in the orthorhombic space group P2(1)2(1)2(1) (Table S1). An observed Si-N distance of 1.717(3) Å reflects the diminished steric demand of the diphenylsilylene unit compared to the respective Si(SiMe_3_)_2_ element of [Et_2_N(SiMe_3_)_2_SiSiMe_2_]_2_ [20], where the Si-N bond length is increased to 1.741(7) Å. A Si-Si-bond length of 2.3744(14) Å for the Et_2_NPh_2_Si–Si bond and other Si-Si lenghts between 2.3540(15) and 2.3706(14) Å (Table 1) as well as Si-Si-Si bond angles close to the ideal tetrahedral angle, characterized compound **1** as a rather typical neopentasilane. The same conclusion could be drawn from the ^29^Si NMR chemical shifts of **1** (Table 2). Resonances at −10.1 and −133.4 ppm for the trimethylsilyl groups and the central silicon are close to that of tetrakis(trimethylsilyl)silane. The shift of +2.0 ppm found for the Et_2_NPh_2_Si group is more unusual. Comparable compounds such as (Et_2_N)Ph_2_Si-SiMe_3_ (−15.3 ppm), (Et_2_N)Ph_2_Si-SiMe_2_-SiPh_2_(NEt_2_) (−15.7 ppm) [25], and (Et_2_N)Ph_2_Si–Si*^t^*Bu_2_H (−8.7 ppm) [26] featured the respective resonances more up-field.

**Scheme 1:**
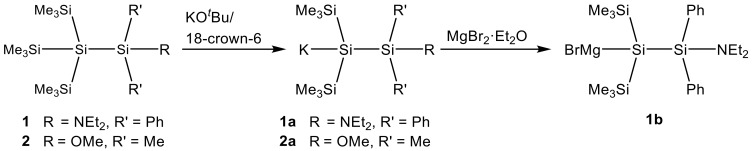
Reactions of aminosilane **1** and alkoxysilane **2** with potassium *tert-*butoxide in C_6_H_6_ in the presence of 18-crown-6 to give β-hetero-substituted oligosilanides **1a** and **2a**. For **1a,** transmetallation with MgBr_2_ to magnesium silanide **1b** was carried out.

**Figure 2:**
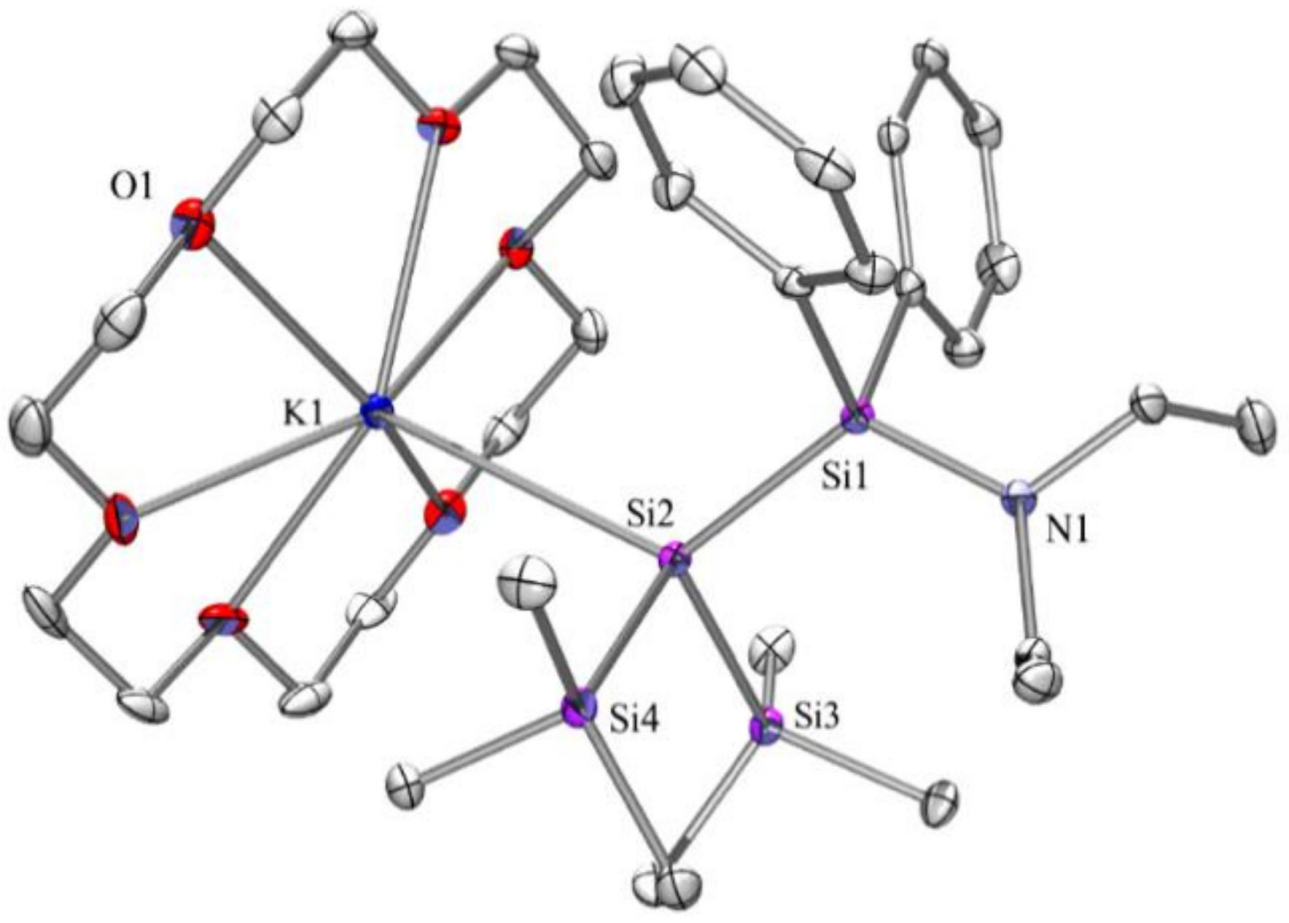
Molecular structure of **1a** (thermal ellipsoid plot drawn at the 30% probability level). All hydrogen atoms are omitted for clarity (bond lengths in Å, angles in degrees). Si(2)-Si(1) 2.3293(10), Si(2)-Si(4) 2.3345(10), Si(2)-Si(3) 2.3510(9), Si(2)-K(1) 3.4990(10), Si(4)-C(1) 1.892(3), Si(1)-N(1) 1.743(2), K(1)-O(1) 3.044(5), N(1)-C(21) 1.454(3), C(7)-C(8) 1.394(3), Si(1)-Si(2)-Si(4) 107.14(4), Si(1)-Si(2)-Si(3) 108.28(4), Si(4)-Si(2)-Si(3) 104.42(4), Si(1)-Si(2)-K(1) 123.29(3), Si(4)-Si(2)-K(1) 98.59(3), Si(3)-Si(2)-K(1) 112.87(3), N(1)-Si(1)-Si(2) 121.46(8).

The reaction of compound **1** with potassium *tert*-butoxide in the presence of 18-crown-6 [22] led to the formation of silanide **1a** (Scheme 1). Crystal structure analysis of **1a** (Figure 2), which crystallizes in the triclinic space group P-1, showed a silanide with shortened Si-Si bond lengths close to 2.33 Å (Table 1). A sum of Si-Si-Si bond angles of 319.85 deg showed a fairly pyramidalized silanide atom. The potassium ion appeared coordinated by the crown ether, and the Si-K distance of 3.5064(19) Å was within the range usually observed for this type of compounds. No intramolecular N^…^K interaction could be observed, but a weak interaction of the NEt_2_ group with a neighboring molecule might be possible. The Si–N distance 1.743(2) of **1a** is clearly large compared to that of 1.717(3) Å for **1**, which likely reflects a repulsive interaction between the lone pairs on nitrogen and silicon.

When comparing the structural parameters of **1a** to those of FPh_2_Si(Me_3_Si)_2_SiK·18-crown-6 [19], we found that the contraction of the central Si-Si bond was not so pronounced. This can be due to the fact that the disilene adduct character of **1a** is smaller than that of FPh_2_Si(Me_3_Si)_2_SiK·18-crown-6. Further comparison with the recently reported Ph_3_Si(Me_3_Si)_2_SiK·18-crown-6 [27] showed a high degree of similarity, which was further emphasized by the ^29^Si-NMR characterization of **1a** (Table 2), featuring the silanide resonance at −186.6 ppm and the signal for SiMe_3_ at −6.3 ppm (δ for Ph_3_Si(Me_3_Si)_2_SiK: −189.0 and −5.8 ppm [27]). The expected down-field shift for the SiPh_2_NEt_2_ caused the respective signal to appear at +16.3 ppm.

**Figure 3:**
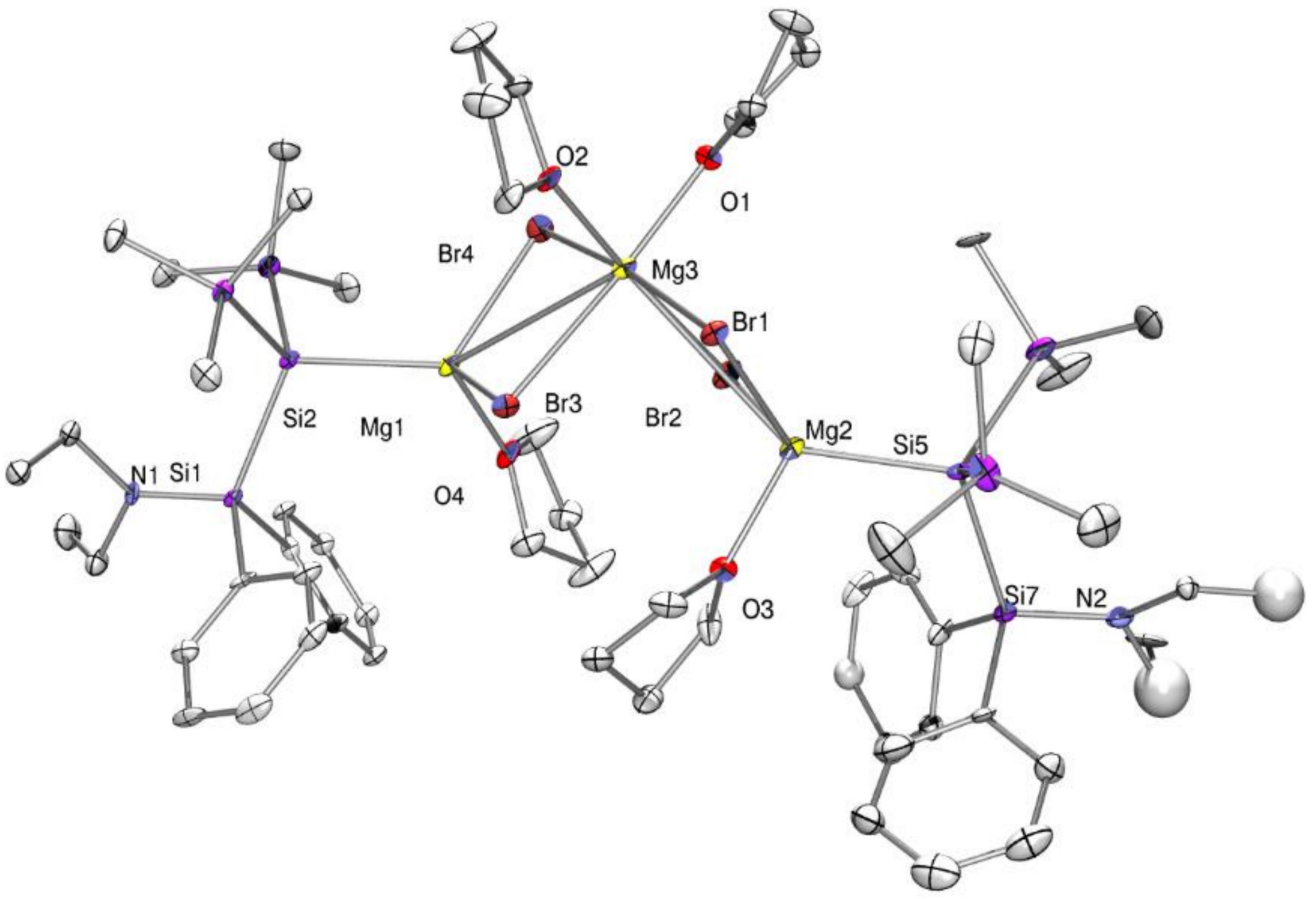
Molecular structure of **1b** (thermal ellipsoid plot drawn at the 30% probability level). Only the anion part of the molecule is shown. All hydrogen atoms are omitted for clarity (bond lengths in Å, angles in degrees). The right silanide part of the structure (Si(5)-Si(7) part) is disordered, and therefore no data on this part are listed. Si(1)-Si(2) 2.338(3), Si(2)-Mg(1) 2.601(4), Si(1)-N(1) 1.730(7), Mg(1)-O(4) 2.009(6), Mg(1)-Br(3) 2.531(3), Mg(1)-Mg(3) 3.641(4), Mg(2)-O(3) 2.029(7), Mg(2)-Br(2) 2.533(3), Mg(2)-Br(1) 2.569(3), Mg(2)-Mg(3) 3.696(4), Mg(3)-O(2) 2.067(6), Mg(3)-O(1) 2.082(6), Mg(3)-Br(4) 2.665(3), Mg(3)-Br(3) 2.723(3), Si(1)-Si(2)-Si(3) 112.69(12), Si(1)-Si(2)-Mg(1) 113.57(12), N(1)-Si(1)-Si(2) 114.4(3), Br(4)-Mg(1)-Br(3) 94.94(10), O(4)-Mg(1)-Si(2) 112.9(2), Br(4)-Mg(1)-Si(2) 121.50(12), Br(3)-Mg(1)-Si(2) 119.85(12), Br(2)-Mg(2)-Br(1) 93.28(10).

Frequently, in reactions with redox labile metal salts, potassium silanides turn out to be too reducing. To moderate the reactivity of these compounds, we found it convenient to transmetallate them to magnesium by metathesis reaction with MgBr_2_·Et_2_O [28,29]. The application of this method on **1a** caused the formation of **1b** (Scheme 1). Usually, we prepare silyl magnesium compounds *in situ* from the respective potassium compounds. Assuming quantitative conversion both for silanide formation and for transmetallation reactions, the stoichiometry of the magnesium silanide is determined by the amount of oligosilane starting material. Typically, we use an excess of MgBr_2_·Et_2_O for reasons of convenience. While more MgBr_2_ has no effect on reactivity, single-crystal XRD analysis of **1b** provided us with an example where an additional equivalent of MgBr_2_ co-crystallized with two molecules of Et_2_NPh_2_Si(Me_3_Si)_2_SiMgBr (Figure 3).

The slightly less anionic character of **1b**, compared to **1a**, is nicely reflected by its crystal structure. The central Si–Si bond distance of 2.338(3) Å is slightly longer than in **1a**, conversely, the Si-N distance of 1.730(7) is somewhat shortened, and the sum of bond angles (328.6(1) deg) is larger, indicating diminished pyramidalization. This is consistent with ^29^Si-NMR shifts of −165.5 ppm for the silanide atom and of +8.7 ppm for the SiPhNEt_2_ group (Table 2). Again, this is caused by the increased covalent character of the Si-Mg bond compared the to the Si-K interaction of **1a**.

In a related way, tris(trimethylsilyl)dimethylmethoxysilylsilane (**2**) [30] was treated in C_6_H_6_ with potassium *tert*-butoxide in the presence of 18-crown-6 (Scheme 1). The fact that the respective methoxylated silanide **2a** was formed at all is somewhat surprising, as we previously observed that in the reaction of either chloro- or fluorodimethylsilyltris(trimethylsilyl)silane with potassium *tert*-butoxide, the attack of the alkoxide occurred exclusively at the halodimethysilyl group. It seems likely that the selectivity in the reaction of **2** does not depend on a stronger steric shielding of the methoxydimethysilyl versus the halodimethysilyl group but rather is due to the fact that the formation of a *tert-*butoxydimethysilyl group seems thermodynamically not feasible. The same way as FPh_2_SiSi(SiMe_3_)_2_K is structurally related to F(Me_3_Si)_2_SiSi(SiMe_3_)_2_K, compound **2a** is related to MeO(Me_3_Si)_2_SiSi(SiMe_3_)_2_K, which we obtained from the self-condensation of MeO(Me_3_Si)_2_SiK [13].

**Figure 4:**
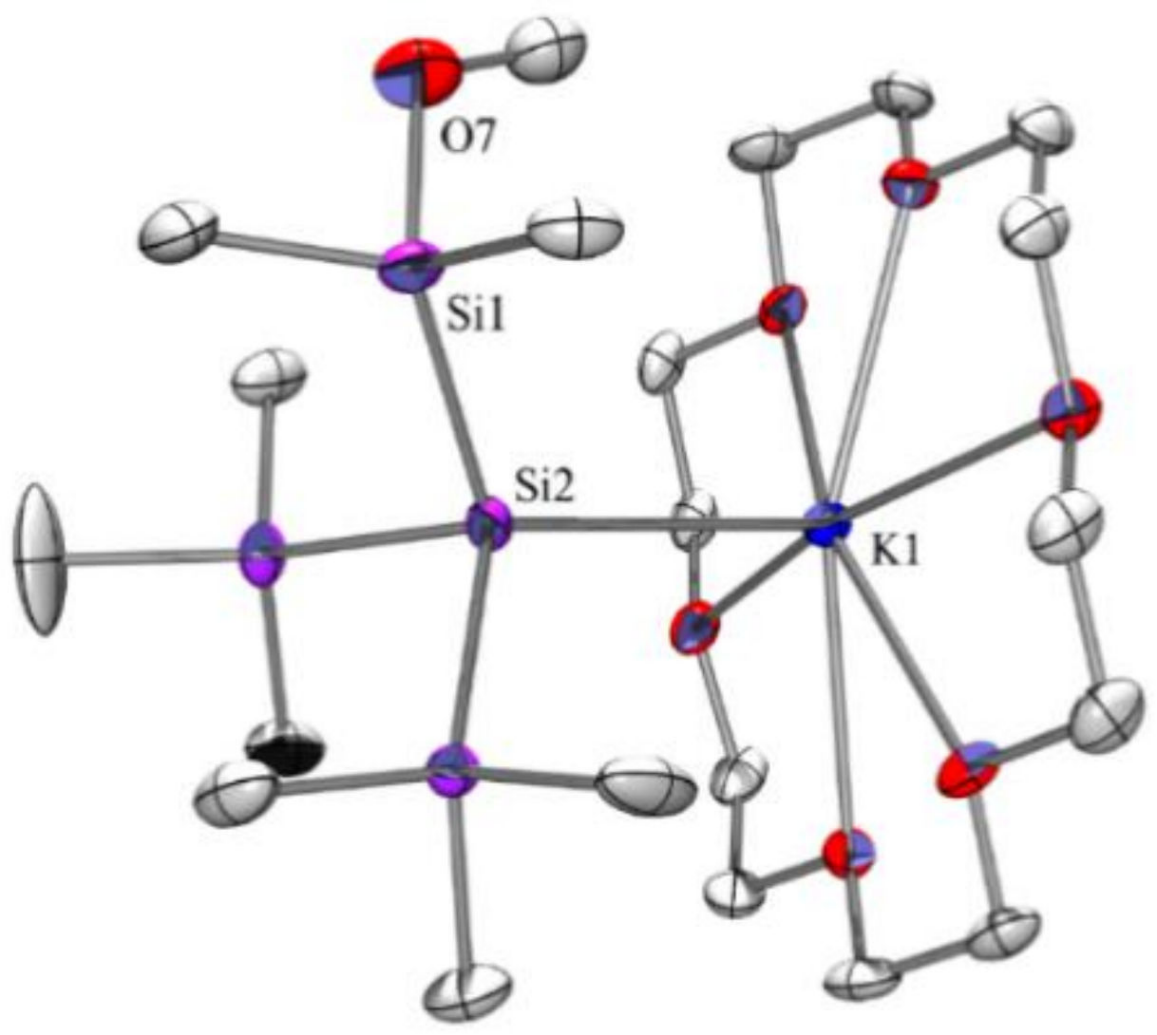
Molecular structure of **2a** (thermal ellipsoid plot drawn at the 30% probability level). All hydrogen atoms are omitted for clarity (bond lengths in Å, angles in degrees). Si(1)-Si(2) 2.329(2), Si(2)-Si(4) 2.331(2), Si(2)-K(1) 3.5064(19), Si(1)-O(7) 1.678(5), O(7)-C(1) 1.320(8), Si(1)-Si(2)-Si(4) 101.85(8), Si(1)-Si(2)-Si(3) 101.19(8), Si(4)-Si(2)-Si(3) 102.42(7), Si(1)-Si(2)-K(1) 122.17(7).

Single-crystal XRD analysis of **2a** (Figure 4) revealed it to be a typical isotetrasilanide with a high degree of pyramidalization (sum of bond angles: 305.46(8) deg), a short Si–SiOMe bond distance of 2.329(2) Å (2.3361(13) Å was found for MeO(Me_3_Si)_2_SiSi(SiMe_3_)_2_K), and a somewhat longer Si–O distance of 1.678(5) Å. Compared to **1a**, the stronger pyramidalization is consistent with a more shielded ^29^Si NMR resonance at −201.8 ppm (Table 2). Judging the fact that the silanide resonance of MeO(Me_3_Si)_2_SiSi(SiMe_3_)_2_K was observed at −170.4 ppm, it can be concluded that MeO(Me_3_Si)_2_SiSi(SiMe_3_)_2_K can be regarded as a methoxide adduct of a disilene [13], whereas **2a** is better described as a β-methoxydisilanide.

**Scheme 2:**
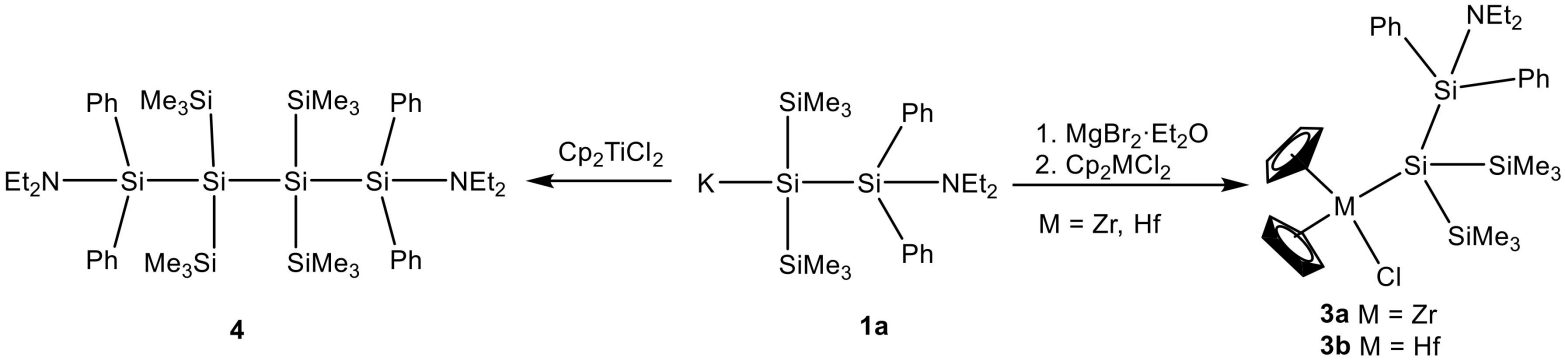
Reactions of **1a** with Cp_2_MCl_2_ (M = Zr, Hf) producing the respective β-aminodisilanyl metallocene chlorides **3a** and **3b**. Reaction of **1a** with Cp_2_TiCl_2_ causes oxidative coupling, yielding **4**.

Over the last years, we have prepared numerous oligosilanylated zirconocenes and hafnocenes, mostly by reaction of either potassium or magnesium oligosilanides with the respective group 4 metallocene dichlorides [31,32,33,34,35,36]. Reactions of **1b** with zirconocene and hafnocene dichlorides proceeded analogously and provided access to complexes **3a** and **3b** (Scheme 2).

**Figure 5:**
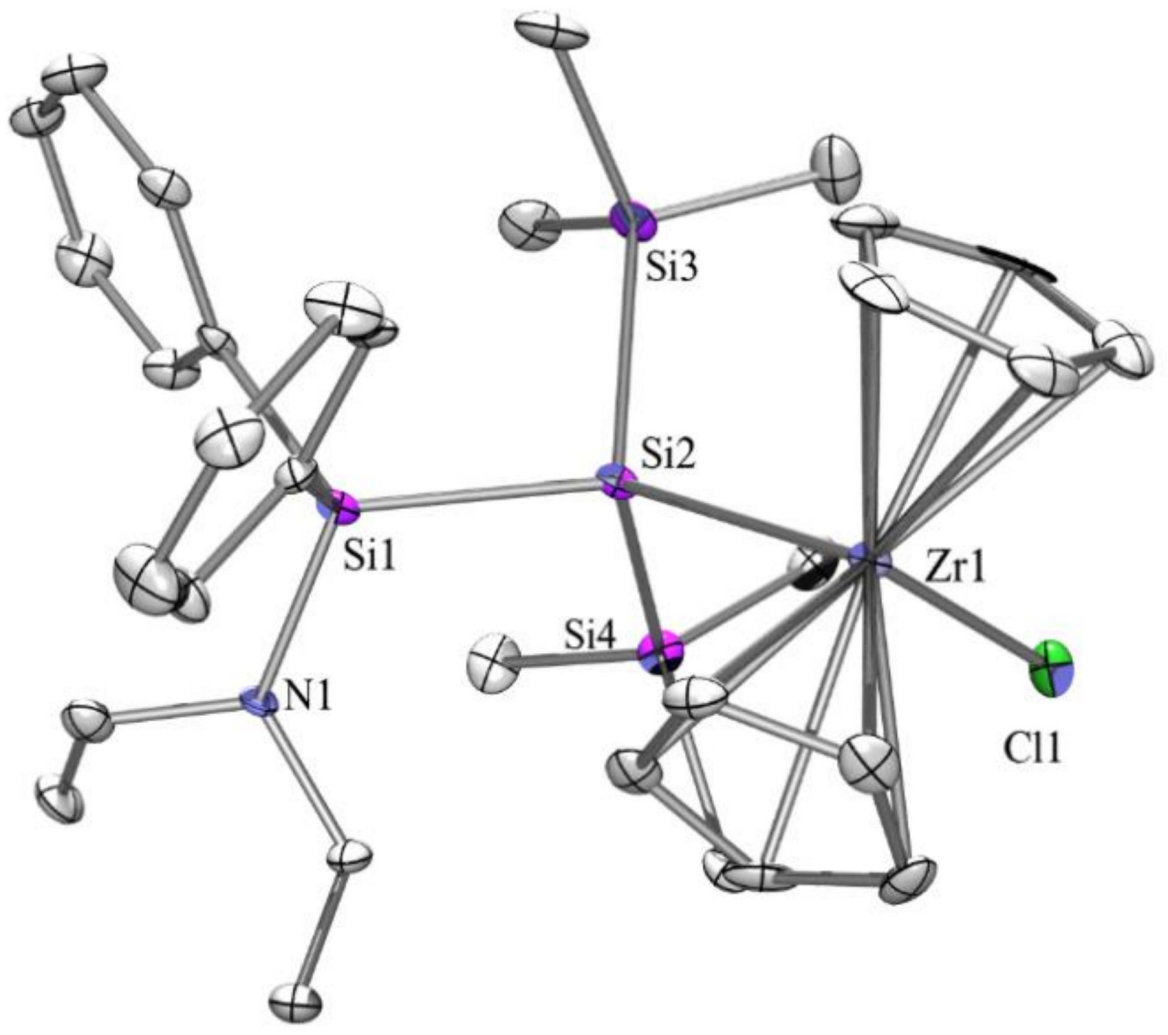
Molecular structure of **3a** (thermal ellipsoid plot drawn at the 30% probability level). All hydrogen atoms are omitted for clarity. About 20% of the chloride position is occupied by bromide (bond lengths in Å, angles in degrees). Cl(1)-Zr(1) 2.4565(19), Zr(1)-Si(2) 2.863(2), Si(1)-Si(2) 2.387(3), Si(1)-N(1) 1.741(6), Si(1)-C(15) 1.881(8), N(1)-C(11) 1.438(10), C(1)-C(2) 1.392(13), Si(3)-Si(2)-Si(1) 104.05(12), Si(3)-Si(2)-Zr(1) 116.95(10), Si(1)-Si(2)-Zr(1) 119.45(10), N(1)-Si(1)-Si(2) 113.2(2).

**Figure 6:**
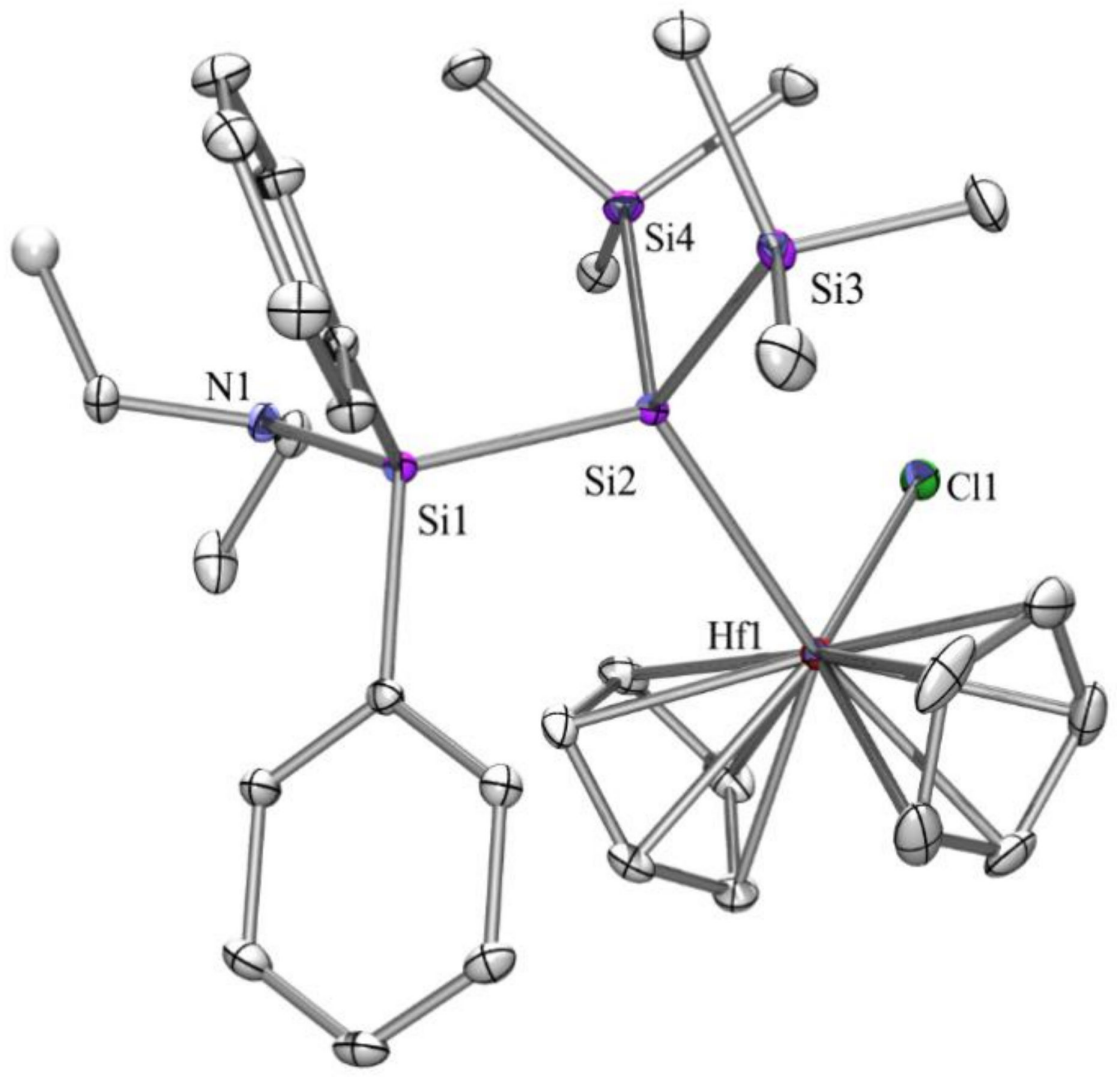
Molecular structure of **3b** (thermal ellipsoid plot drawn at the 30% probability level). All hydrogen atoms are omitted for clarity (bond lengths in Å, angles in degrees). Hf(1)-Cl(1) 2.3998(8), Hf(1)-Si(2) 2.8339(8), Si(1)-Si(2) 2.3937(9), Si(1)-N(1) 1.7315(19), Si(1)-C(17) 1.891(2), Cl(1)-Hf(1)-Si(2) 93.12(3), Si(3)-Si(2)-Si(1) 103.81(4), Si(3)-Si(2)-Hf(1) 117.25(3), Si(1)-Si(2)-Hf(1) 119.29(3), N(1)-Si(1)-Si(2) 113.67(7).

In the course of the reaction between **1b** (prepared *in situ* by addition of MgBr_2_·Et_2_O to a solution of **1a**) and Cp_2_ZrCl_2_ (Scheme 2), the formed compound **3a** reacted by about 20% with the formed MgClBr to yield Cp_2_Zr(Br)Si(SiMe_3_)_2_SiPh_2_NEt_2_ as a side product. The presence of the latter was recognized in the crystal structure of **3a** (Figure 5). Both the fairly covalent interaction between Si and Zr as well as the steric demand of the Cp_2_Zr(Cl) unit caused an elongation of the central Si-Si distance of the Si(SiMe_3_)_2_SiPh_2_NEt_2_ moiety to 2.387(3) Å. The observed Si-Zr bond length of 2.863(2) Å is close to those found previously for Cp_2_Zr(Cl)Si(SiMe_3_)_2_SiMe_2_Thex (2.853 Å) and Cp_2_Zr[Si(SiMe_3_)_3_]_2_ (2.878 Å) [32]. However, compared to the structurally related complex Cp_2_Zr(Cl)Si(SiMe_3_)_2_SiPh_2_F (d_Si-Zr_ = 2.799(1)/2.803(1) Å) [19], the bond is significantly longer. As expected, complex **3b** (Figure 6) was found to be isostructural to **3a**, with the Si-Hf bond (2.8339(8) Å) being slightly shorter than the Si-Zr bond of **3a**. The ^29^Si NMR resonances of the metallated silicon atoms of **3a** and **3b** were observed at −89.5 and −83.1 ppm, respectively. These values are close to those found for the structurally related compounds Cp_2_Zr(Cl)Si(SiMe_3_)_3_ (−89.5 ppm) and Cp_2_Hf(Cl)Si(SiMe_3_)_3_ (−79.7 ppm) [32]. The recently reported Cp_2_Zr(Cl)Si(SiMe_3_)_2_SiPh_2_F exhibited a very similar value of −90.9 ppm [19].

**Figure 7:**
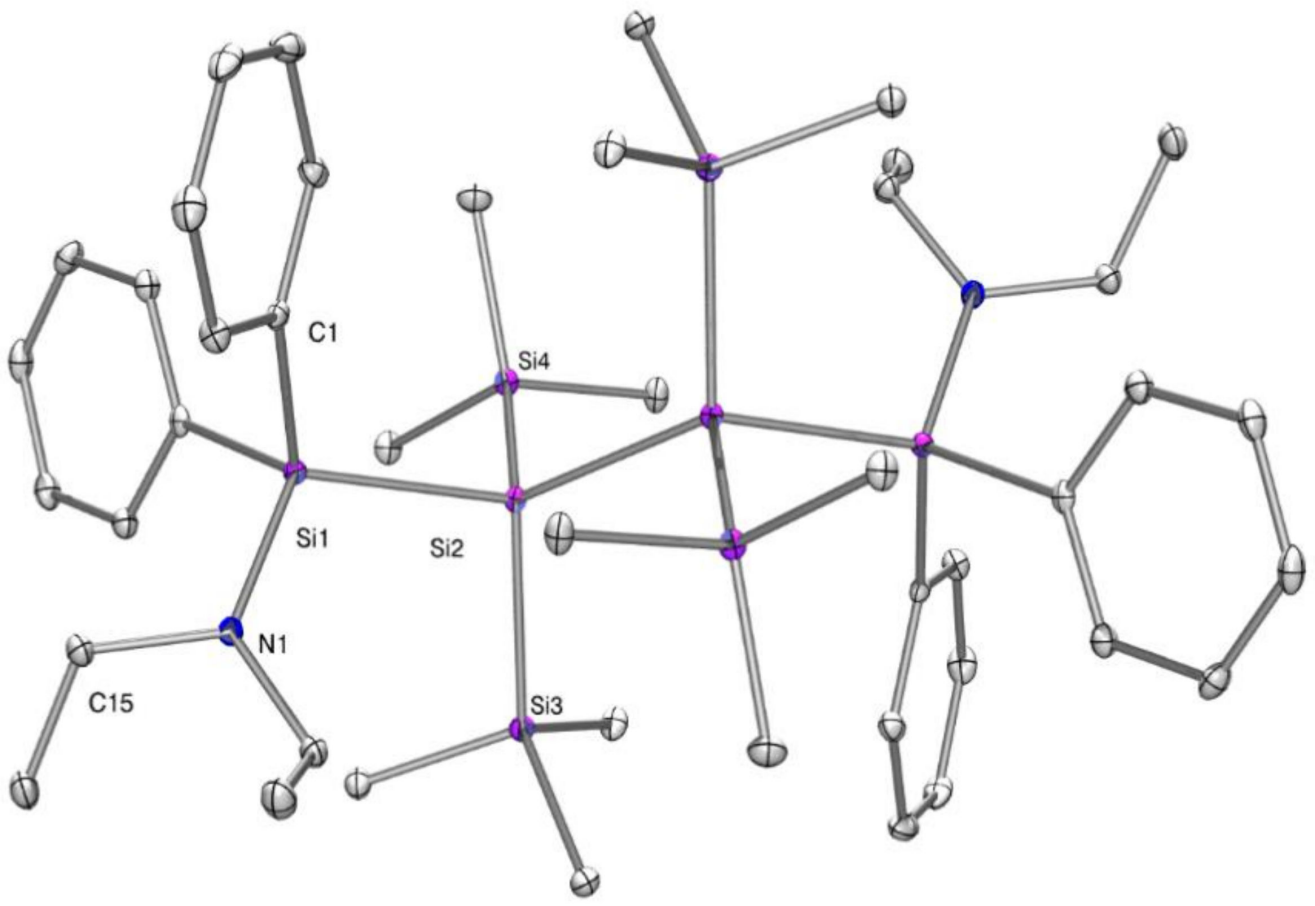
Molecular structure of **4** (thermal ellipsoid plot drawn at the 30% probability level). All hydrogen atoms are omitted for clarity (bond lengths in Å, angles in degrees). Si(1)-Si(2) 2.426(2) Si(2)-Si(4) 2.394(3), Si(2)-Si(3) 2.410(3), Si(2)-Si(2A) 2.464(4), Si(1)-C(18) 1.875(4), Si(1)-N(1) 1.727(3), N(1)-C(15) 1.473(4), Si(1)-Si(2)-Si(2a) 117.04(5), Si(3)-Si(2)-Si(4) 97.85(5).

An analogous reaction of **1a** with Cp_2_TiCl_2_ did not give the respective isotetrasilanyl titanocene chloride but the oxidative coupling product **4** (Scheme 2). This is not entirely surprising, as we found out earlier that disilylated titanocenes in the oxidation state +4 tend to undergo reductive elimination of disilanes [34,35]. The crystal structure of **4** (Figure 7) featured a rather long central Si-Si bond distance of 2.464(4) Å. Comparable hexasilylated disilanes such as (Me_3_Si)_3_SiSi(SiMe_3_)_3_ (2.403(2) Å) [37,38], (Et_3_Si)_3_SiSi(SiEt_3_)_3_ (2.417(1) Å) [39], and PhMe_2_Si(Me_3_Si)_2_SiSi(SiMe_3_)_2_SiMe_2_Ph (2.4166(9) Å) [40] exhibit substantially shorter central bonds. Of all acyclic compounds of this type, only H*^t^*Bu_2_Si(Me_3_Si)_2_SiSi(SiMe_3_)_2_Si*^t^*Bu_2_H (2.4896(8) Å) [41] was reported to exhibit a longer bond. For the tetrasilane unit, a *trans*-conformation was found, with a torsional angle of 180 deg and diethylamino substituents on different sides of the plane defined by the main chain. The ^29^Si-NMR resonance (−115.4 ppm, Table 2) of the central silicon atoms of **4** reflects its extended branched octasilane framework [42].

**Scheme 3:**
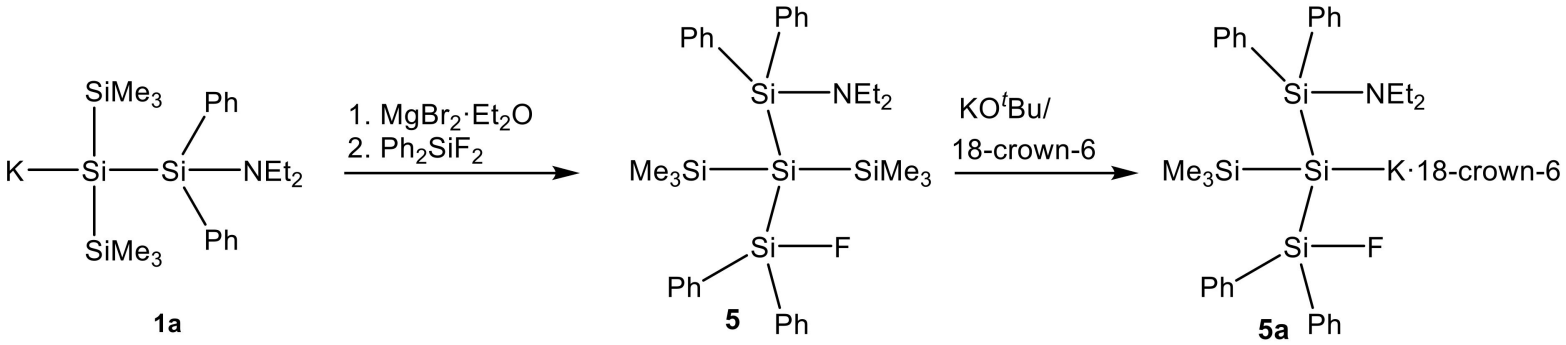
Synthesis of amino- and fluoro-substituted neopentasilane **5**, which serves as the starting material of **5a**.

Since compound **1a** appeared somewhat different from FPh_2_Si(Me_3_Si)_2_SiK [19], we wondered if it was possible to prepare a silanide containing both the Et_2_NPh_2_Si and the FPh_2_Si groups. For this reason, we reacted **1a** with Ph_2_SiF_2_, obtaining neopentasilane **5** in good yield (Scheme 3). The reaction of the latter with KO*^t^*Bu in the presence of 18-crown-6 gave access to the respective silanide **5a** (Scheme 3) at low temperature (−30 °C). Unfortunately, single-crystal XRD analysis of **5a** was not possible, but its NMR spectroscopic properties, in particular the ^1^*J*_SiF_ coupling constant and the SiK and SiPh_2_F chemical shifts, were very similar to those of FPh_2_Si(Me_3_Si)_2_SiK [19].

**Scheme 4:**
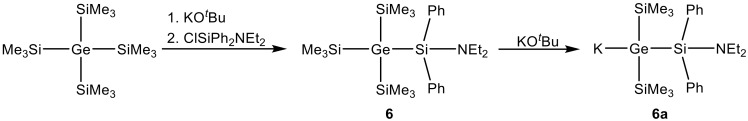
Two-step synthesis of **6a** starting from tetrakis(trimethylsilyl)germane.

As tris(trimethylsilyl)germyl potassium is a readily available compound [43], we decided to extend the chemistry of β-aminooligosilanides to that of aminosilyl-substituted germanides. For this reason, we reacted tris(trimethylsilyl)germyl potassium with Et_2_NPh_2_SiCl to obtain diethylaminodiphenylsilyltris(trimethylsilyl)germane (**6**), which in a subsequent step, was converted to potassium diethylaminodiphenylsilylbis(trimethylsilyl)germanide **6a** by reaction with KO*^t^*Bu (Scheme 4). The compound can be regarded as the diethylamide adduct of a silagermene.

As could be expected, ^1^H and ^13^C-NMR spectroscopic properties of **6** and **6a** were fairly close to those of **1** and **1a**. The ^29^Si-NMR spectra of **6** and **6a** displayed the typical behavior of silylated germanes, where the silyl resonances were shifted a few ppm towards a lower field [43].

## 3. Materials and Methods

### 3.1. General Remarks

All reactions involving air-sensitive compounds were carried out under an atmosphere of dry nitrogen or argon using either Schlenk techniques or a glove box. All solvents were dried using a column-based solvent purification system [44]. Chemicals were obtained from different suppliers and used without further purification.

^1^H (300 MHz), ^13^C (75.4 MHz), ^19^F (282.2 MHz), and ^29^Si (59.3 MHz) NMR spectra were recorded on a Varian INOVA 300 spectrometer. If not noted otherwise, all samples were measured in C_6_D_6_. To compensate for the low isotopic abundance of ^29^Si, the INEPT pulse sequence was used for the amplification of the signal [45,46]. Frequently, this did not allow observing diphenylsilyl Si signals; therefore, a simple inverse-gated single pulse experiment was used for those cases. Elementary analyses were carried out using a Heraeus VARIO ELEMENTAR instrument. As potassium and magnesium silanides usually give poor analysis data, the purity of these compounds was confirmed by ^1^H, ^13^C and ^29^Si-NMR spectra.

### 3.2. X-Ray Structure Determination

For X-ray structure analyses, the crystals were mounted onto the tip of a glass fiber, and data collection was performed with a BRUKER-AXS SMART APEX CCD diffractometer using graphite-monochromated Mo Kα radiation (0.71073 Å). The data were reduced to F^2^_o_ and corrected for absorption effects with SAINT [47] and SADABS [48,49], respectively. The structures were solved by direct methods and refined by a full-matrix least-squares method (SHELXL97) [50]. If not noted otherwise, all non-hydrogen atoms were refined with anisotropic displacement parameters. All hydrogen atoms were located in calculated positions to correspond to standard bond lengths and angles. All diagrams were drawn with 30% probability thermal ellipsoids, and all hydrogen atoms were omitted for clarity. Crystallographic data (excluding structure factors) for the structures of compounds **1**, **2a**, **1a**, **1b**, **3a**, **3b**, and **4** reported in this paper are deposited with the Cambridge Crystallographic Data Center as supplementary publication no. CCDC-1952884 (**1**), 1952880 (**2a**), 1952885 (**1a**), 1952883 (**1b**), 1952881 (**3a**), 1952886 (**3b**), and 1952882 (**4**). Copies of data can be obtained free of charge at: http://www.ccdc.cam.ac.uk/products/csd/request/. The figures of solid-state molecular structures were generated using Ortep-3 as implemented in WINGX [51] and rendered using POV-Ray 3.6 [52].

Tetrakis(trimethylsilyl)silane [53], N,N-diethylaminochlorodiphenylsilane [23,24], MgBr_2_·OEt_2_ [54], titanocene dichloride [55], and tetrakis(trimethylsilyl)germane [56] were prepared following published procedures.

#### 3.2.1. N,N-Diethylaminodiphenylsilyltris(trimethylsilyl)silane (**1**)

A solution of tris(trimethylsilyl)silyl potassium (freshly prepared from tetrakis(trimethylsilyl)silane (1.50 g, 4.68 mmol) and KO*^t^*Bu (0.54 g, 4.81 mmol) in THF (10 mL)) was added dropwise to a vigorously stirred solution of N,N-diethylaminochlorodiphenylsilane (1.36 g, 4.71 mmol) in THF (10 mL). After the addition, the yellow reaction mixture was stirred for 1 d at ambient temperature, after which all volatiles were evaporated under reduced pressure. The slightly yellow residue was extracted with pentane, and the concentrated extracts were stored at −50 °C to yield colorless crystals of **1** (1.45 g, 62%). NMR (δ in ppm): ^1^H: 7.77 (m, 4H), 7.22 (m, 6H), 2.11 (q, 4H, *J* = 7.1 Hz and 14.3 Hz, *CH_2_*CH_3_), 0.73 (t, 6H, *J* = 7.2 Hz, CH_2_*CH_3_*), 0.30 (s, 27H, SiMe_3_). ^13^C: 141.2, 134.7, 129.5, 127.9, 43.4, 14.6, 3.1. ^29^Si: 2.0 (SiPh_2_NEt_2_), −10.1 (SiMe_3_), −133.4 (Si_q_). Anal. calcd. for C_25_H_47_NSi_5_ (502.08): C 59.81, H 9.44, N 2.79. Found: C 59.61, H 9.45, N 2.76.

#### 3.2.2. N,N-Diethylaminodiphenylsilylbis(trimethylsilyl)silyl Potassium 18-Crown-6 (**1a**)

Compound **1** (500 mg, 0.996 mmol), KO*^t^*Bu (117 mg, 1.05 mmol), and 18-crown-6 (276 mg, 1.05 mmol) were dissolved in a minimum amount of benzene. The color of the mixture immediately turned to orange. After a few minutes, the reaction was finished as could be determined by ^29^Si-NMR spectroscopy of an aliquot sample. The solvent was removed in vacuum, and pentane was added to the residue. Product **1a** (171 mg, 32%) was crystallized as deep-orange crystals from pentane. NMR (δ in ppm): ^1^H: 8.14 (m, 4H, Ph), 7.27-7.36 (m, 6H, Ph), 3.58 (q, *J*_HH_ = 7 Hz, 4H, NC*H_2_*CH_3_), 3.13 (s, 24H, 18cr6), 1.19 (t, *J*_HH_ = 7 Hz, 6H, NCH_2_C*H_3_*), 0.54 (s, 18H, SiMe_3_). ^13^C: 148.1, 136.6, 126.9, 126.8, 70.0 (18cr6), 41.4 (N*C*H_2_CH_3_), 15.1 (NCH_2_*C*H_3_), 7.7 (SiMe_3_). ^29^Si: 16.3 (SiPh_2_NEt_2_), −6.3 (SiMe_3_), −186.6 (Si_q_).

#### 3.2.3. N,N-Diethylaminodiphenylsilylbis(trimethylsilyl)silyl Magnesium Bromide (**1b**)

Compound **1b** was prepared according to **1a**, with **1** (200 mg, 0.40 mmol) and KO*^t^*Bu (47 mg, 0.42 mmol). Instead of work-up after 3 h, the mixture was dropped to MgBr_2_·OEt_2_ (108 mg, 0.42 mmol) in THF (1 mL) and stirred for 3 h. Then, all volatiles were evaporated under reduced pressure, and the yellowish residue was extracted with benzene/pentane (3:1). The combined extracts were concentrated to a volume of about 0.5 mL, and addition of pentane (10 mL) afforded the precipitation of **1b** as a colorless microcrystalline solid (231 mg, 34%). NMR (δ in ppm: ^1^H: 7.90 (m, 4H, Ph), 7.26 (t, *J*_HH_ = 7 Hz, 3H, Ph), 7.14-7.18 (m, 3H, Ph), 3.61 (br s, 8H, OC*H_2_*CH_2_), 3.27 (q, *J*_HH_ = 7 Hz, 4H, NC*H_2_*CH_3_), 1.25 (br s, 8H, OCH_2_C*H_2_*), 1.04 (t, *J*_HH_ = 7 Hz, 6H, NCH_2_C*H_3_*), 0.44 (s, 18H, SiMe_3_). ^13^C: 143.8, 136.2, 128.3, 127.8, 69.8 (O*C*H_2_CH_2_), 41.7 (N*C*H_2_CH_3_), 25.1 (OCH_2_*C*H_2_), 15.0 (NCH_2_*C*H_3_), 5.6 (SiMe_3_). ^29^Si (inverse-gated): 8.7 (SiPh_2_NEt_2_), −8.3 (SiMe_3_), −165.5 (SiMg).

#### 3.2.4. Methoxydimethylsilyltris(trimethylsilyl)silane (**2**)

Procedure according to **1**, using: tetrakis(trimethylsilyl)silane (1000 mg, 3.12 mmol), KO*^t^*Bu (367 mg, 3.27 mmol), MgBr_2_·OEt_2_ (845 mg, 3.27), and Me_2_SiCl_2_ (422 mg, 3.27 mmol) which was added at 0 °C. After 12 h, the solvent was removed, and the residue was treated with pentane. The solvent was again removed, and the obtained slight-yellow oil dissolved in DME (10 mL) and dropped to a cooled (0 °C) solution of DME (50 mL), MeOH (105 mg, 3.27 mmol), and Et_3_N (331 mg, 3.27 mmol). After 12 h, the solvent was removed, and the residue was treated with pentane. Compound **2** (787 mg, 75%) was obtained as a colorless oil. NMR (δ in ppm): ^1^H: 3.28 (s, 3H), 0.37 (s, 6H), 0.30 (s, 27H). ^13^C: 50.5, 3.9, 2.9. ^29^Si: 24.5 (SiMe_2_OMe), −10.44 (SiMe_3_), −136.5 (Si_q_). Anal. calcd. for C_12_H_36_OSi_5_ (336.84): C 42.79, H 10.77. Found: C 42.58, H 10.80.

#### 3.2.5. 2-Methoxydimethylsilyltris(trimethylsilyl)silyl Potassium 18-Crown-6 (**2a**)

Same procedure as for **1a**, using: **2** (200 mg, 0.59 mmol), KO*^t^*Bu (70 mg, 0.62 mmol), and 18-crown-6 (165 mg, 0.62 mmol). Product **2a** (128 mg, 38%) was crystallized as deep-orange crystals from pentane. NMR (δ in ppm): ^1^H: 3.42 (s, 3H, OMe), 3.12 (s, 24H, 18-c-6) 0.26 (s, 18H, SiMe_3_), 0.21 (s, 6H, SiMe_2_). ^13^C: 70.1 (18-c-6), 50.4 (OMe), 7.6 (SiMe_2_), 7.5 (SiMe_3_). ^29^Si: 40.0 (SiMe_2_OMe), −4.5 (SiMe_3_), −201.8 (Si_q_).

#### 3.2.6. N,N-Diethylaminodiphenylsilylbis(trimethylsilyl)silyl Zirconocene Chloride (**3a**)

A cold solution of **1b** (449 mg, 1.00 mmol) (stored at −35 °C prior to the reaction) in THF (2 mL) was added dropwise to a cold solution of Cp_2_ZrCl_2_ (350 mg, 1.10 mmol) in THF (5 mL) under vigorous stirring. After the addition, the orange reaction mixture was kept at −35 °C for another 1 h, followed by evaporation of all volatiles under reduced pressure. The orange residue was extracted with benzene/pentane (1:2 ratio), the solutions were combined and evaporated to dryness, and the solid residue was washed with pentane (3 × 3 mL). The remaining solid was taken up in benzene (3 mL),w and the solution was layered with pentane, which afforded red crystalline **3a** (520 mg, 73%). NMR (δ in ppm): ^1^H: 7.90 (m, 4H, Ph), 7.22-7.28 (m, 4H, Ph), 7.14-7.19 (m, 2H, Ph), 5.92 (s, 10H, Cp), 3.19 (q, *J*_HH_ = 7 Hz, 4H, NC*H_2_*CH_3_), 1.02 (t, *J*_HH_ = 7 Hz, 6H, NCH_2_C*H_3_*), 0.47 (s, 18H, SiMe_3_). ^13^C: 142.2, 136.4, 129.0, 127.8, 112.1 (Cp), 41.7 (N*C*H_2_CH_3_), 14.0 (NCH_2_*C*H_3_), 5.7 (SiMe_3_). ^29^Si (inverse-gated): 7.6 (SiPh_2_NEt_2_), −6.7 (SiMe_3_), −89.5 (SiZr). Anal. calcd. for C_32_H_48_Br_0.2_Cl_0.8_NSi_4_Zr (694.65): C 55.33, H 6.96, N 2.02. Found: C 55.15, H 7.18, N 1.96.

#### 3.2.7. N,N-Diethylaminodiphenylsilylbis(trimethylsilyl)silyl Hafnocene Chloride (**3b**)

Reaction was done according to **3a**, using **1b** (1.00 mmol) and Cp_2_HfCl_2_ (449 mg, 1.10 mmol). Recrystallization with pentane afforded orange crystalline **3b** (552 mg, 69%). NMR (δ in ppm, C_6_D_6_): ^1^H: 7.91 (m, 4H, Ph), 7.23-7.28 (m, 4H, Ph), 7.16-7.18 (m, 2H, Ph), 5.82 (s, 10H, Cp), 3.20 (q, *J*_HH_ = 7 Hz, 4H, NC*H_2_*CH_3_), 1.03 (t, *J*_HH_ = 7 Hz, 6H, NCH_2_C*H_3_*), 0.47 (s, 18H, SiMe_3_). ^13^C: 142.5, 136.5, 128.9, 127.7, 111.1 (Cp), 41.7 (N*C*H_2_CH_3_), 14.0 (NCH_2_*C*H_3_), 5.9 (SiMe_3_). ^29^Si (inverse-gated): 9.5 (SiPh_2_NEt_2_), −5.7 (SiMe_3_), −83.1 (SiHf). Anal. calcd. for C_32_H_48_ClHfNSi_4_ (773.02): C 49.72, H 6.26, N 1.81. Found: C 50.08, H 6.08, N 1.84.

#### 3.2.8. 1,2-Bis(N,N-diethylaminodiphenylsilyl)-1,1,2,2,-Tetrakis(trimethylsilyl)disilane (**4**)

To a solution of **1** (400 mg, 0.80 mmol) in THF (3 mL), KO*^t^*Bu (94 mg, 0.84 mmol) was added. The color immediately turned to dark reddish. The reaction mixture was stirred for 15 h, whereupon the conversion was quantitative according to NMR measurements, and the solution was cooled to −78 °C. A solution of Cp_2_TiCl_2_ in THF (1 mL) was added dropwise to the reaction mixture, and stirring was continued at rt for 24 h. The solvent was removed in vacuo, and the residue was dissolved in pentane. The precipitated salts were removed by decantation, and the product was crystallized from pentane by slow evaporation, yielding crystalline colorless **4** (274 mg, 80%). NMR (δ in ppm): ^1^H: 7.83 (m, 8H, Ph), 7.22 (m, 12H, Ph), 3.06 (q, *J*_HH_ = 7 Hz, 8H, N*CH_2_*CH_3_), 0.95 (t, *J*_HH_ = 7 Hz, 12H, NCH_2_*CH_3_*), 0.34 (s, 36H, SiMe_3_). ^13^C: 140.5, 137.4, 129.5, 42.7, 14.3, 6.3. ^29^Si (inverse-gated): 4.8 (SiPh_2_NEt_2_), −7.9 (SiMe_3_), −115.4 (*Si*(SiMe_3_)_2_). Anal. calcd. for C_44_H_76_N_2_Si_8_ (857.79): C 61.61, H 8.93, N 3.27. Found: C 61.37, H 9.01 N 3.23.

#### 3.2.9. N,N-Diethylaminodiphenylsilyl(fluorodiphenylsilyl)bis(trimethylsilyl)silane (**5**)

A suspension of **1b** (freshly prepared from **1** (645 mg, 1.29 mmol), KO*^t^*Bu (152 mg, 1.36 mmol), and MgBr_2_·OEt_2_ (352 mg, 1.36 mmol)) in THF (9 mL) was added dropwise under vigorous stirring to difluorodiphenylsilane at ambient temperature. After the addition, the off-white reaction mixture was stirred for another 21 h, and then all volatiles were evaporated under reduced pressure. The remaining white solid was extracted with toluene/pentane (1:4), and the combined extracts were dried under vacuum, yielding **5** as colorless microcrystals (730 mg, 90%). NMR (δ in ppm): ^1^H: 7.56-7.62 (m, 8H, Ph), 7.13-7.15 (m, 6H, Ph), 7.06 (m, 6H, Ph), 3.00 (q, *J*_HH_ = 7 Hz, 4H, NC*H_2_*CH_3_), 0.86 (t, *J*_HH_ = 7 Hz, 6H, NCH_2_C*H_3_*), 0.24 (s, 18H, SiMe_3_). ^13^C: 139.3, 138.5 (d, *J*_CF_ = 13 Hz, Ph), 136.2, 134.3 (d, *J*_CF_ = 3 Hz, Ph), 130.0, 129.5, 128.1, 128.0, 41.8 (N*C*H_2_CH_3_), 14.6 (NCH_2_*C*H_3_), 3.4 (SiMe_3_). ^19^F: −168.6 (d, *J*_SiF_ = 318 Hz). ^29^Si (inverse-gated): 20.1 (d, *J*_SiF_ = 318 Hz, SiPh_2_F), 0.3 (d, *J*_SiF_ = 6 Hz, SiPh_2_NEt_2_), −9.2 (SiMe_3_), −131.2 (d, *J*_SiF_ = 19 Hz, Si_q_). Anal. calcd. for C_34_H_48_FNSi_5_ (630.19): C 64.80, H 7.68, N 2.22. Found: C 64.66, H 7.70, N 2.27.

#### 3.2.10. N,N-Diethylaminodiphenylsilyl(fluorodiphenylsilyl)(trimethylsilyl)silyl Potassium 18-Crown-6 (**5a**)

The reaction was done according to **1a**, using **5** (100 mg, 0.16 mmol), KO*^t^*Bu (19 mg, 0.17 mmol), and 18-crown-6 (44 mg, 0.17 mmol). Recrystallization from pentane at −60 °C afforded orange crystalline **5a** (102 mg, 75%). NMR (δ in ppm, D_2_O capillary, THF, −30 °C, reaction solution): ^1^H: 7.55 (m, 8H), 6.95 (m, 12H), 3.03 (q, *J* = 7Hz, 4H, C*H*_2_CH_3_), 0.70 (t, *J* = 7Hz, 6H, CH_2_C*H*_3_), −0.47 (s, 9H, SiMe_3_). ^13^C: 148.3 (d, *J*_CF_ = 17 Hz, Ph), 146.8, 136.3, 134.5, 126.7, 126.3, 126.2, 71.0, 40.8, 14.6, 6.4. ^19^F: −165.6 (^1^*J_Si-F_* = 357 Hz). ^29^Si (inverse-gated): 36.0 (d, ^1^*J_Si-F_* = 359 Hz, SiPh_2_F), 14.9 (SiPh_2_NEt_2_), −6.6 (d, ^3^*J_Si-F_* = 5 Hz, SiMe_3_), −198.9 (SiK).

#### 3.2.11. N,N-Diethylaminodiphenylsilyltris(trimethylsilyl)germane (**6**)

A solution of tris(trimethylsilyl)germyl potassium (freshly prepared from tetrakis(trimethylsilyl)germane (1.10 g, 3.00 mmol) and KO*^t^*Bu (354 mg, 3.15 mmol) in THF (6 mL)) was added dropwise to a vigorously stirred solution of chloro(diethylamino)diphenylsilane in THF (20 mL). The yellowish reaction mixture was stirred for another 0.5 h after the addition was finished. Then, all volatiles were evaporated to dryness, and the yellow residue was extracted with pentane. The yellow extracts were concentrated to a volume of about 4 mL and stored at −35 °C for crystallization, yielding **6** as colorless crystals (230 mg, 14%). NMR (δ in ppm): ^1^H: 7.71-7.74 (m, 4H, Ph), 7.15-7.24 (m, 6H, Ph), 3.08 (q, *J*_HH_ = 7 Hz, 4H, NC*H_2_*CH_3_), 0.96 (t, *J*_HH_ = 7 Hz, 6H, NCH_2_C*H_3_*), 0.27 (s, 27H, SiMe_3_). ^13^C: 140.3 (Ph), 136.0 (Ph), 129.4 (Ph), 127.9 (Ph), 41.7 (N*C*H_2_CH_3_), 14.8 (NCH_2_*C*H_3_), 3.8 (SiMe_3_). ^29^Si (inverse-gated): 4.4 (SiPh_2_NEt_2_), −5.4 (SiMe_3_). Anal. calcd. for C_25_H_47_GeNSi_4_ (546.63): C 54.93, H 8.67, N 2.56. Found: C 54.78, H 8.82, N 2.56.

#### 3.2.12. N,N-Diethylaminodiphenylsilylbis(trimethylsilyl)germyl Potassium 18-Crown-6 (**6a**)

A vial was charged with **6** (273 mg, 0.500 mmol), KO*^t^*Bu (59 mg, 0.53 mmol), 18-crown-6 (139 mg, 0.525 mmol), and benzene (4 mL). The reaction mixture turned yellow immediately and was left standing for 4 h. Then, all volatiles were evaporated under reduced pressure. The yellow residue was washed with pentane and dried under vacuum, yielding **6a** as a yellow microcrystalline solid (241 mg, 31%). NMR (δ in ppm): ^1^H: 8.11-8.14 (m, 4H, Ph), 7.16-7.37 (m, 6H, Ph), 3.57 (q, *J*_HH_ = 7 Hz, 4H, N*CH_2_*CH_3_), 3.15 (s, 24H, 18cr6), 1.19 (t, *J*_HH_ = 7 Hz, 6H, NCH_2_*CH_3_*), 0.56 (s, 18H, SiMe_3_). ^13^C: 148.4 (Ph), 136.5 (Ph), 126.9 (Ph), 126.7 (Ph), 70.0 (18cr6), 41.6 (N*CH_2_*CH_3_), 15.2 (NCH_2_*CH_3_*), 7.9 (SiMe_3_). ^29^Si (inverse-gated): 17.6 (SiPh_2_NEt_2_), −5.0 (SiMe_3_).

## 4. Conclusions

Some years ago, we studied the chemistry of α-fluoro- [15,16], alkoxy- [13] and amino-substituted [20] oligosilanides. For fluoro- and alkoxysilanides, we found a tendency to self-condensation, yielding β-fluoro- and alkoxydisilanides. Unusual spectroscopic and chemical properties suggested that these compounds should be regarded as base adducts of symmetrical disilenes. Recently, we reported the synthesis of some β-halodisilanides with different substituents at the 1- and 2-positions of the disilane unit. NMR spectroscopic, structural, and chemical characterization clearly showed that the disilene adduct character of the novel compounds was much diminished.

The preparation and characterization of β-N,N-diethylaminodisilanides, outlined in the current study, clearly showed that also these compounds should be regarded as oligosilanides and not as amide disilene adducts. The study includes the conversion of the initially obtained potassium silanides to the respective derivatives of magnesium, zirconium, and hafnium. In addition, we extended this chemistry to a β-N,N-diethylaminodiphenylsilylbis(trimethylsilyl)germanide.

## Figures and Tables

**Table 1:** Structural data of starting material **1,** metallated compounds **1a**, **1b**, **2a**, **3a**, and **3b**, coupling product **4,** and reference compounds XPh_2_Si(Me_3_Si)_3_SiK (X = F, Cl).

Compound	Si^1^-X	Si^1^-Si^2^	Si^2^-SiMe_3_	Si^2^-M
**1**	1.717(3)	2.3744(14)	2.3540(15), 2.3657(15), 2.3706(14)	n.a.
**1a**	1.743(2)	2.3293(10)	2.3345(10), 2.3510(9)	3.4990(10) (K)
**1b**	1.730(7)	2.338(3)		2.601(4) (Mg)
**2a**	1.678(5) (X = O)	2.329(2)	2.331(2) 2.335(2)	3.5064(19) (K)
FPh_2_Si(Me_3_Si)_3_SiK ^[a]^	1.639(2) (X = F)	2.2970(15)	2.341(1)/ 2.347(1)	3.5609(14) (K)
ClPh_2_Si(Me_3_Si)_3_SiK ^[a]^	2.153(2) (X = Cl)	2.315(2)	2.356(2)/ 2.360(2)	n.a.
**3a**	1.741(6)	2.387(3)		2.863(2) (Zr)
**3b**	1.7315(19)	2.3937(9)		2.8339(8) (Hf)
**4**	1.727(3)	2.464(4)	2.394(3), 2.410(3)	n.a.

[a] Data taken from reference [19].

**Table 2:** ^29^Si-NMR chemical shift data for **1**, **1a**, **1b**, **2**, **2a**, **3a**, **3b**, **4**, **5**, **5a**, **6**, **6a** and the reference substances XPh_2_Si(Me_3_Si)_3_SiK (X = F, Cl).

Compound	δ ^29^Si^1^-X (NEt_2_)	δ ^29^Si^2^	δ ^29^SiMe_3_	δ ^29^Si Other
**1**	+2.0	−133.4	−10.1	
**1a**	+16.3	−186.6	−6.3	
**1b**	+8.7	−165.5	−8.3	
**2**	+24.5 (OMe)	−136.5	−10.4	
**2a**	+40.0 (OMe)	−201.8	−4.5	
FPh_2_Si(Me_3_Si)_3_SiK^[a]^	+41.4 (F)	−201.6	−4.4	
ClPh_2_Si(Me_3_Si)_3_SiK^[a]^	+39.7 (Cl)	−195.7	−5.7	
**3a**	+7.6	−89.5	−6.7	
**3b**	+9.5	−83.1	−5.8	
**4**	+4.8	−115.4	−7.9	
**5**	+0.3 (d, *J*_SiF_ = 6 Hz)	−131.2 (d, *J*_SiF_ = 19 Hz)	−9.2	+20.1 (SiPh_2_F) (d, *J*_SiF_ = 318 Hz)
**5a**	+14.9	−198.9 (SiK)	−6.6 (d, ^3^*J_Si–F_* = 5Hz)	+36.0 (SiPh_2_F) (d, *J_Si–F_* = 359 Hz)
**6**	+4.4	n.a.	−5.4	
**6a**	+17.6	n.a	−5.0	

[a] Data taken from reference [19].

## Supplementary Materials

The following are available online at https://www.mdpi.com/1420-3049/24/21/3823/s1, Figures S1–S46: ^1^H, ^13^C, ^19^F, and ^29^Si-NMR spectra of compounds **1**, **1a**, **1b**, **2**, **2a**, **3a**, **3b**, **4**, **5**, **5a**, **6**, and **6a**, Table S1: Crystallographic data for compounds **1**, **1a**, **1b**, **2a**, **3a**, **3b**, and **4**.
