# Supplementary material for: β-Amino- and Alkoxy-Substituted Disilanides
Source: Molecules. 2019 Oct 23;24(21):3823. doi: 10.3390/molecules24213823 (PMC6864825; doi:10.3390/molecules24213823)
Supplement: Supplementary file 1 [file molecules-24-03823-s001.pdf]

Supporting Info for

β-Amino and Alkoxy Substituted Disilanides

submitted to *Molecules*

Istvan Balatoni, Johann Hlina, Rainer Zitz, Alexander Pöcheim,

Judith Baumgartner* and Christoph Marschner

Institut für Anorganische Chemie, Technische Universität Graz, Stremayrgasse 9,

8010 Graz, Austria

Contents

1. Crystallographic table of compounds **31**, **1a**, **1b**, **2a**, **3a**, **3b**, and **4** **S1**
2. NMR spectra
   - Compound **1** **S2**
   - Compound **1a** **S3**
   - Compound **1b** **S5**
   - Compound **2** **S7**
   - Compound **2a** **S9**
   - Compound **3a** **S10**
   - Compound **3b** **S12**
   - Compound **4** **S14**
   - Compound **5** **S16**
   - Compound **5a** **S19**
   - Compound **6** **S21**
   - Compound **6a** **S23**

Table S1. Crystallographic data for compounds **1**, **1a**, **1b**, **2a**, **3a**, **3b**, and **4**

	**1**	**1a**	**1b**	**2a**	**3a**	**3b**	**4**
Empirical formula	$C_{25}H_{47}NSi_5$	$C_{34}H_{64}KNO_6Si_4$	$C_{60}H_{108}Br_4Mg_3O_4N_2Si_8$	$C_{21}H_{51}KO_7Si_4$	$C_{32}H_{48}Br_{0.25}Cl_{0.75}NSi_4Zr$	$C_{32}H_{48l}ClHfNSi_4$	$C_{44}H_{76}N_2Si_8$
M_w	502.09	734.32	1538.77	567.08	696.86	773.01	857.79
Temperature [K]	180(2)	100(2)	100(2)	100(2)	100(2)	110(2)	110(2)
Size [mm]	0.32×0.22×0.17	0.33×0.20×0.12	0.26×0.18×0.12	0.38×0.33×0.28	0.33×0.24×0.14	0.44×0.36×0.28	0.36×0.26×0.16
Crystal system	orthorhombic	triclinic	triclinic	monoclinic	monoclinic	monoclinic	triclinic
Space group	P2(1)2(1)2(1)	P-1	P-1	P2(1)/c	P2(1)/c	P2(1)/c	P-1
a [Å]	9.569(2)	10.849(2)	11.961(4)	16.949(3)	20.710(4)	20.641(4)	10.364(2)
b [Å]	17.036(3)	11.404(2)	17.368(5)	9.572(2)	10.592(2)	10.600(2)	11.648(2)
c [Å]	19.170(4)	19.168(3)	19.951(6)	20.757(4)	16.924(3)	16.960(3)	12.349(2)
α [°]	90	98.780(3)	89.683(5)	90	90	90	108.77(2)
β [°]	90	97.95(3)	73.470(4)	106.33(3)	112.04(3)	112.08(3)	103.49(2)
γ [°]	90	114.01(3)	79.557(5)	90	90	90	107.99(2)
V [Å ^{3}]	3125(2)	2087(2)	3903(2)	3231(2)	3441(2)	3439(2)	1247(3)
Z	4	2	2	4	4	4	1
ρ_{calc} [gcm^{-3}]	1.067	1.168	1.309	1.166	1.345	1.493	1.142
Absorption coefficient [mm^{-1}]	0.241	0.281	2.250	0.345	0.830	3.273	0.246
F(000)	1096	796	1604	1232	1458	1568	466
θ range	1.60<θ<26.37	1.10<θ<26.35	1.56<θ<26.36	2.04<θ<26.37	2.12<θ<25.00	2.13<θ<26.35	1.87<θ<26.47
Reflections collected/unique	22280/6344	16687/8376	30329/15576	24731/6592	23818/6059	25263/6990	9464/4932
Completeness to θ [%]	100	98.3	97.8	99.8	99.9	99.7	95.4
Data/restraints/parameters	6344/0/292	8376/20/597	15576/90/835	6592/0/307	6059/0/361	6990/0/355	4932/0/252
Goodness of fit on F^2	1.34	1.22	1.30	1.04	1.15	1.05	1.19
Final R indices [I>2σ(I)]	R1=0.068, *w*R2=0.143	R1=0.057, *w*R2=0.120	R1=0.115, *w*R2=0.220	R1=0.087, *w*R2=0.176	R1=0.099, *w*R2=0.190	R1=0.020, *w*R2=0.049	R1=0.070, *w*R2=0.136
R indices (all data)	R1=0.071, *w*R2=0.144	R1=0.062, *w*R2=0.122	R1=0.130, *w*R2=0.226	R1=0.158, *w*R2=0.20	R1=0.150, *w*R2=0.209	R1=0.022, *w*R2=0.050	R1=0.083, *w*R2=0.141
Largest diff. Peak/hole [e$^-$/ Å^3]	0.47/−0.38	0.47/−0.56	1.57/−0.89	0.58/-0.50	1.77/−1.74	1.0/−0.48	0.6/−0.37

2. ^{1}H, ^{13}C, ^{19}F, and ^{29}Si NMR spectra

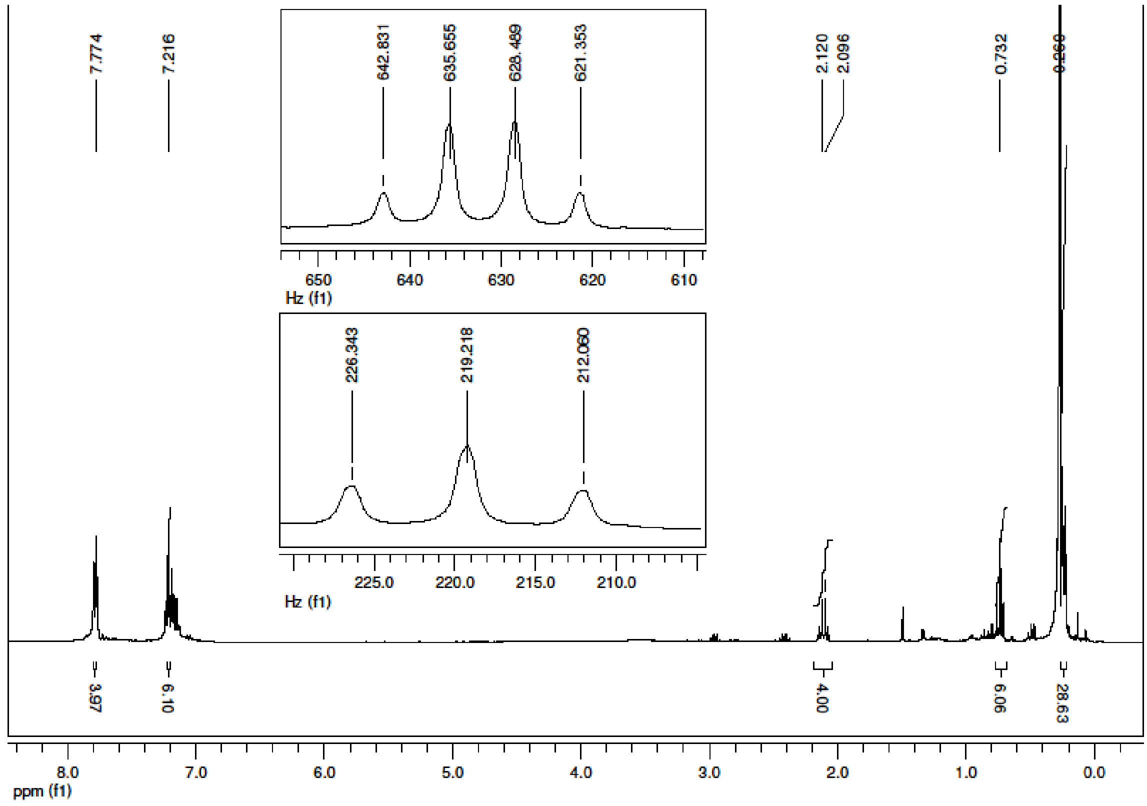

Figure S1. ^{1}H NMR spectrum of **1** in C_6D_6

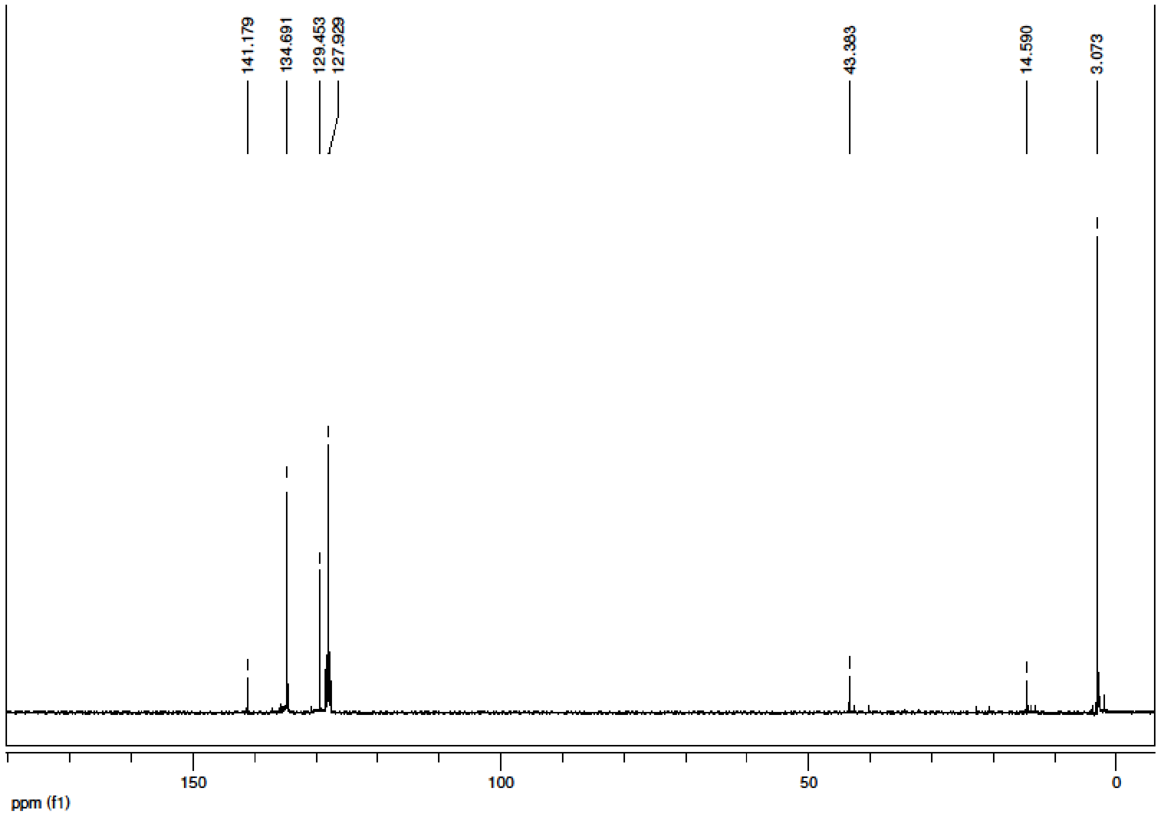

Figure S2. ^{13}C NMR spectrum of **1** in C_6D_6

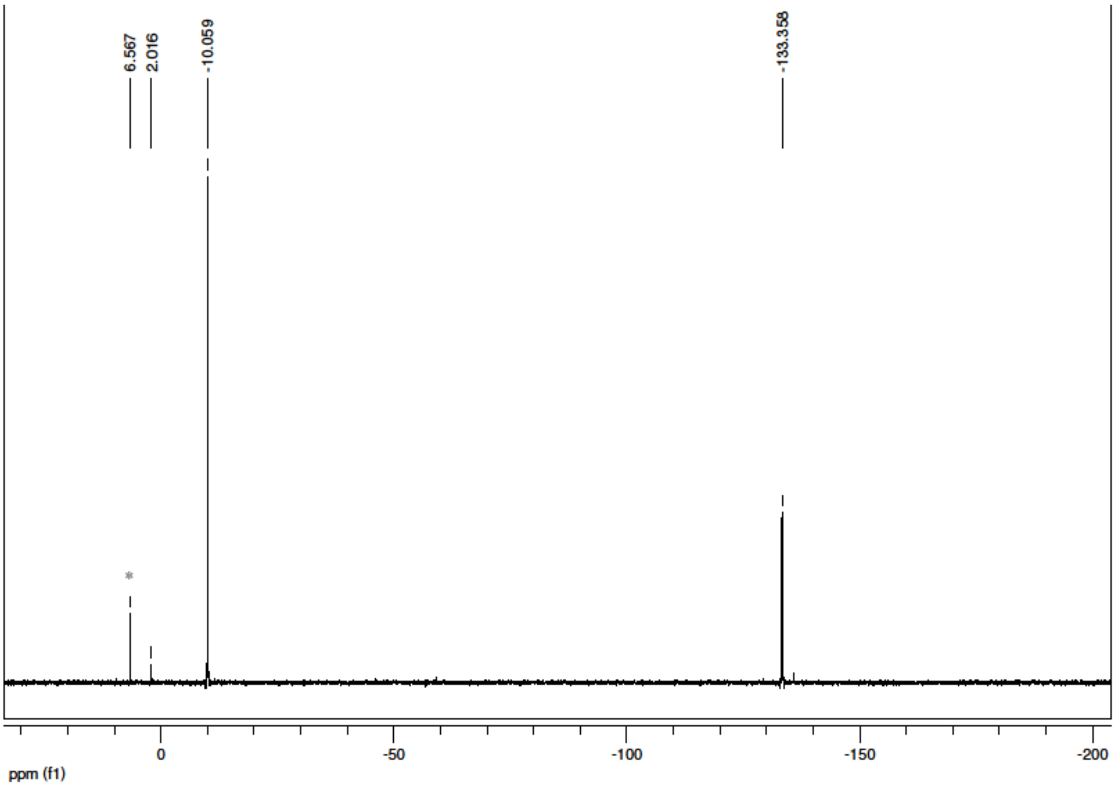

Figure S3. ^{29}Si INEPT NMR spectrum of **1** in C_6D_6 (* = Me_3SiO^tBu)

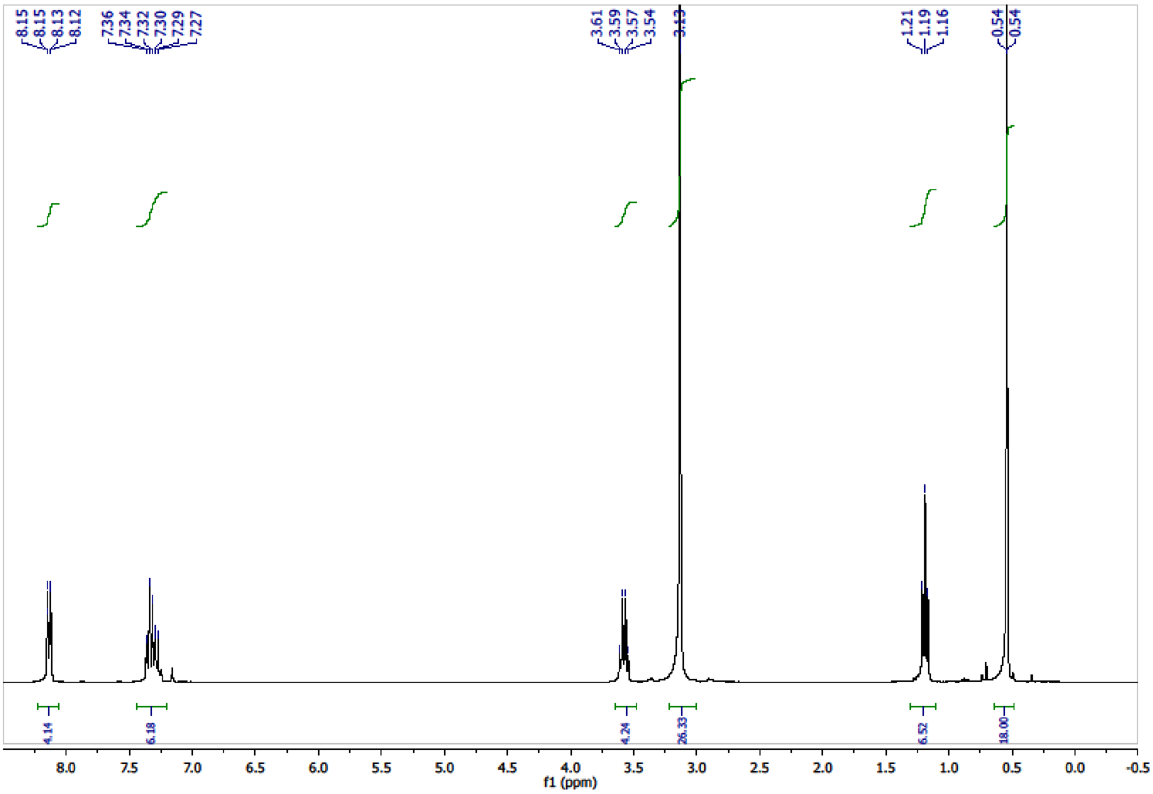

Figure S4. ^1H NMR spectrum of **1a** in C_6D_6

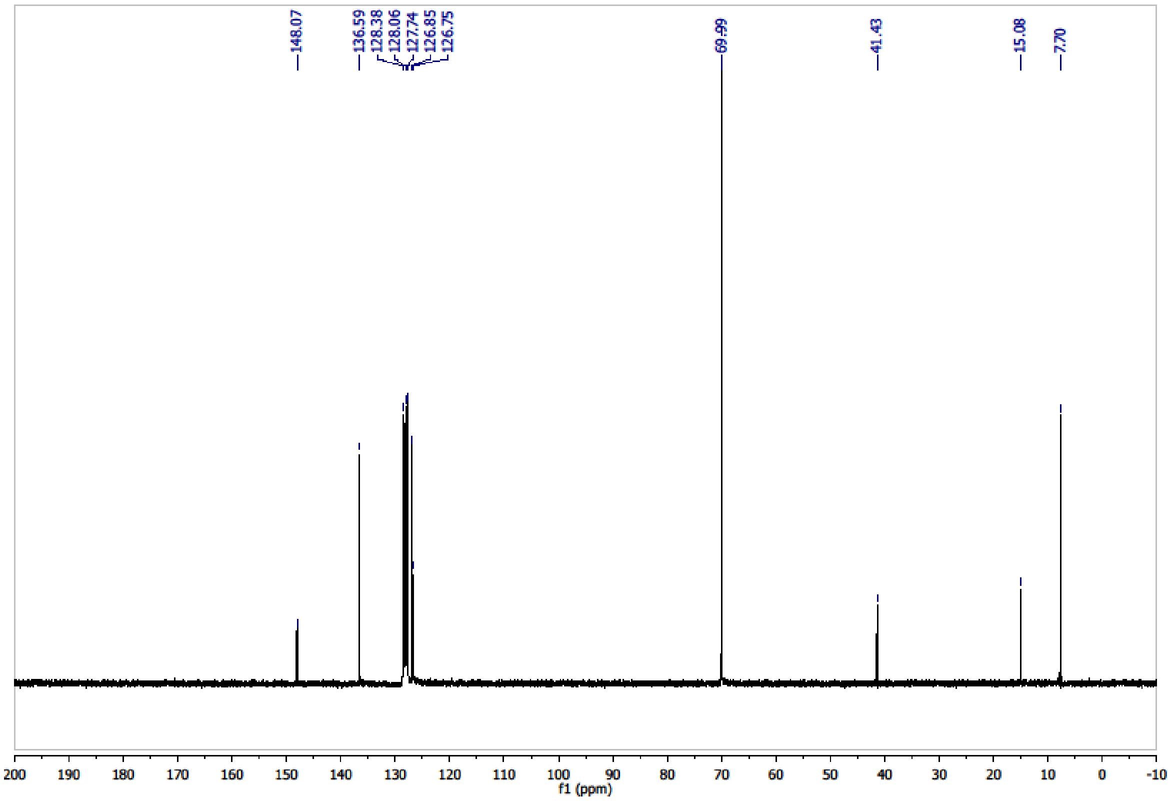

Figure S5. ^{13}C NMR spectrum of **1a** in C_6D_6

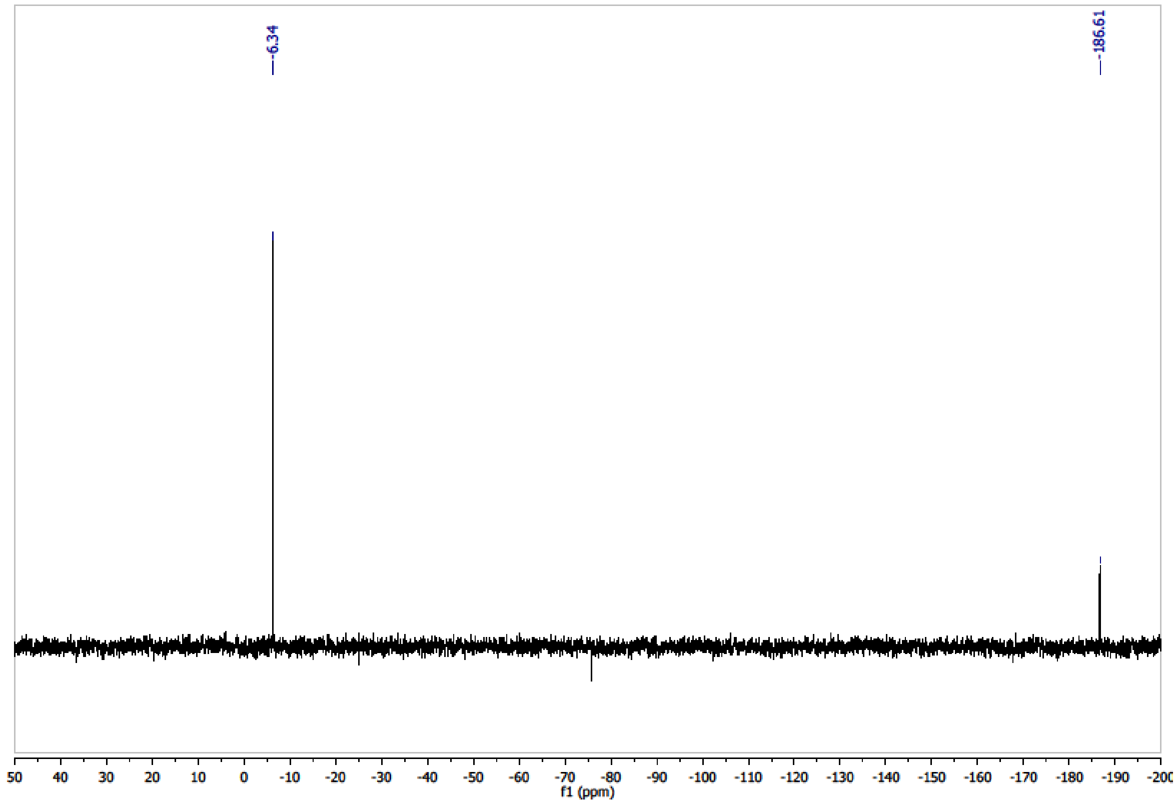

Figure S6. ^{29}Si INEPT NMR spectrum of **1a** in C_6D_6

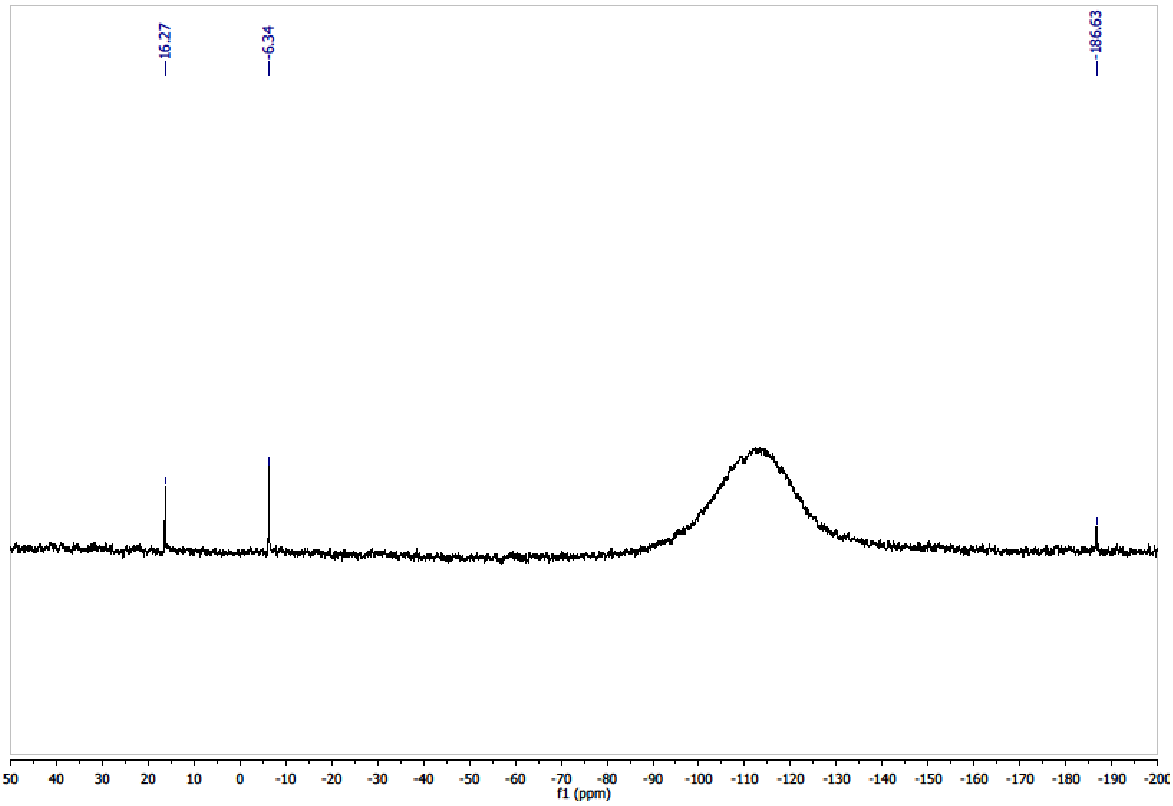

Figure S7. ^{29}Si inverse-gated NMR spectrum of **1a** in C_6D_6

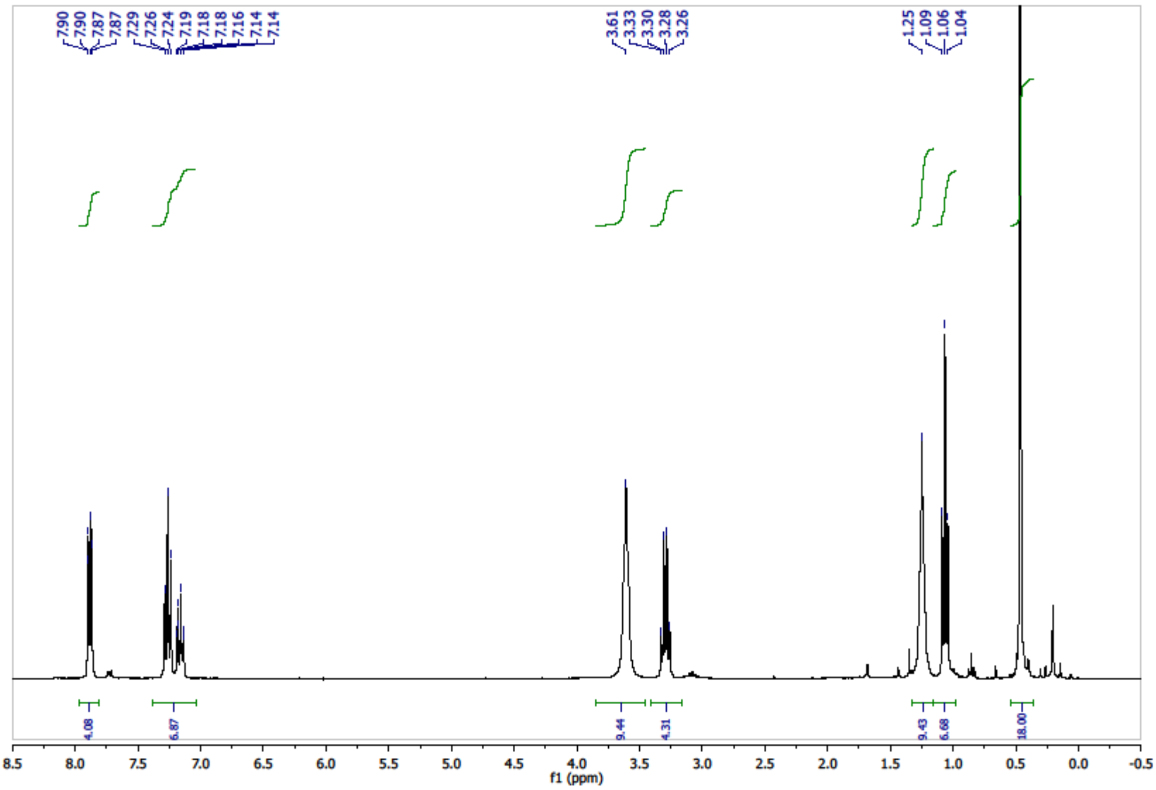

Figure S8. ^{1}H NMR spectrum of **1b** in C_6D_6

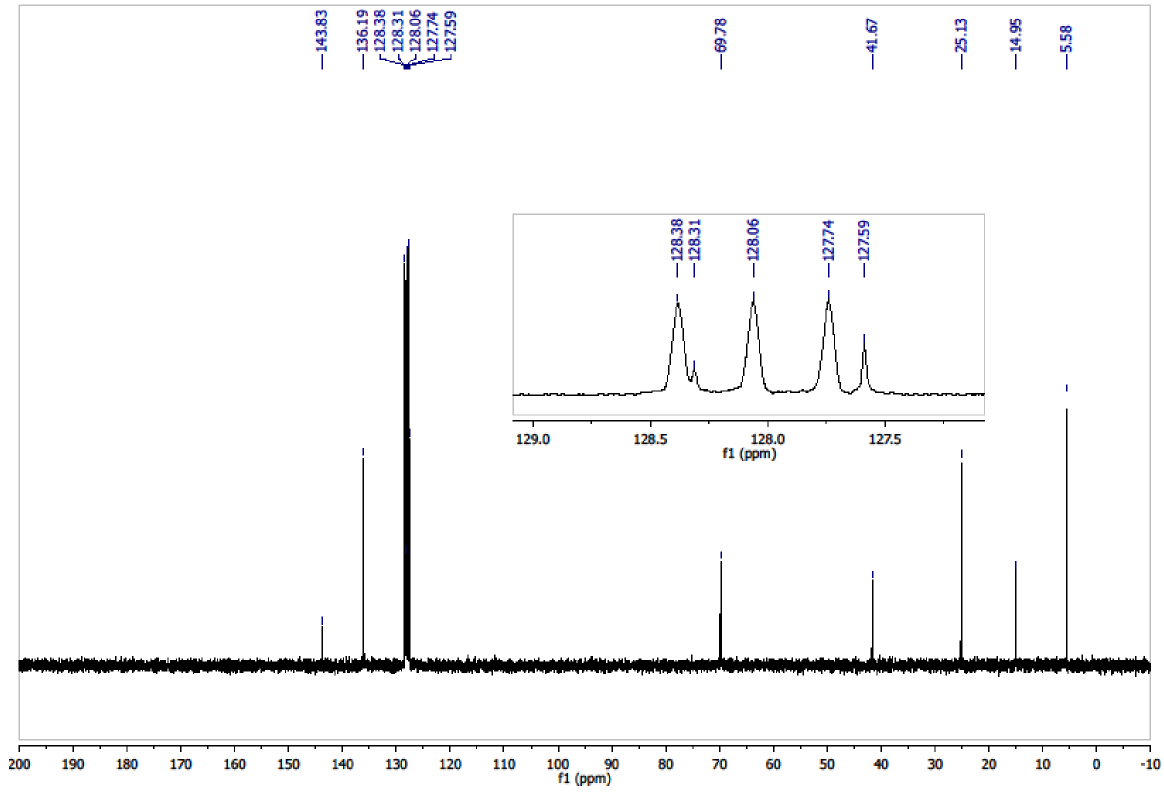

Figure S9. ^{13}C NMR spectrum of **1b** in C_6D_6

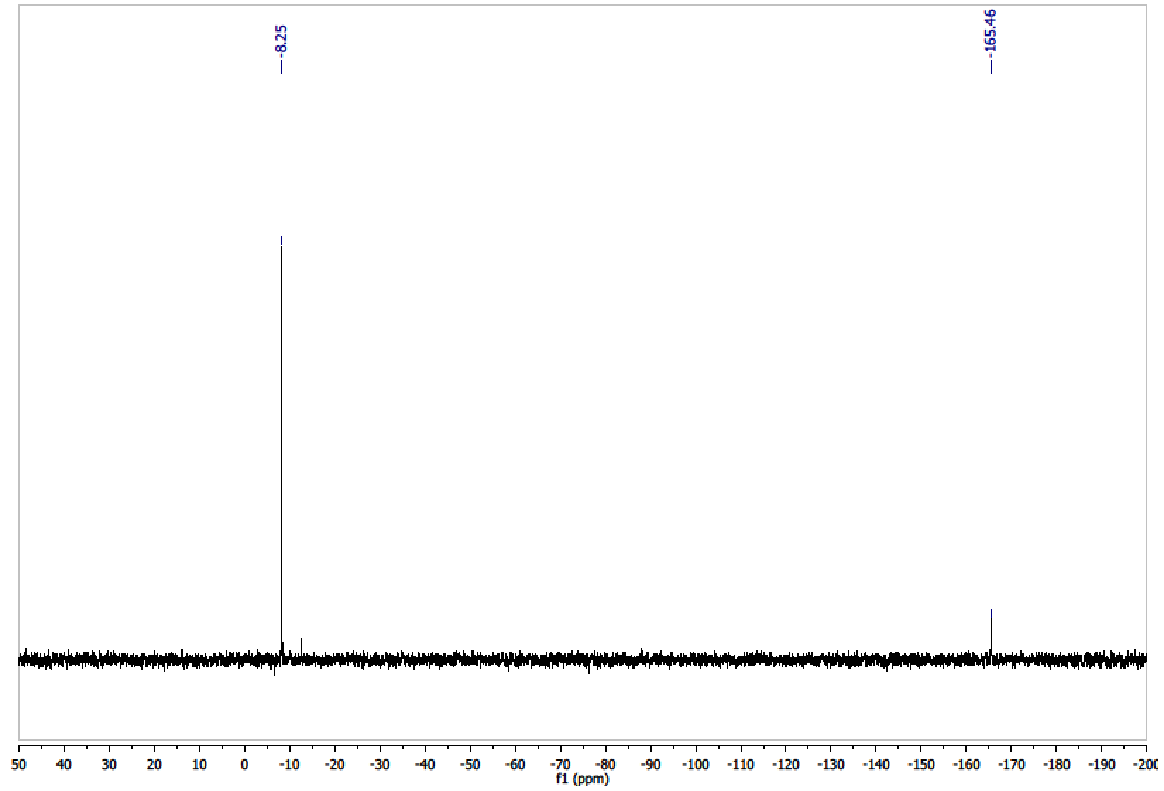

Figure S10. ^{29}Si INEPT NMR spectrum of **1b** in C_6D_6

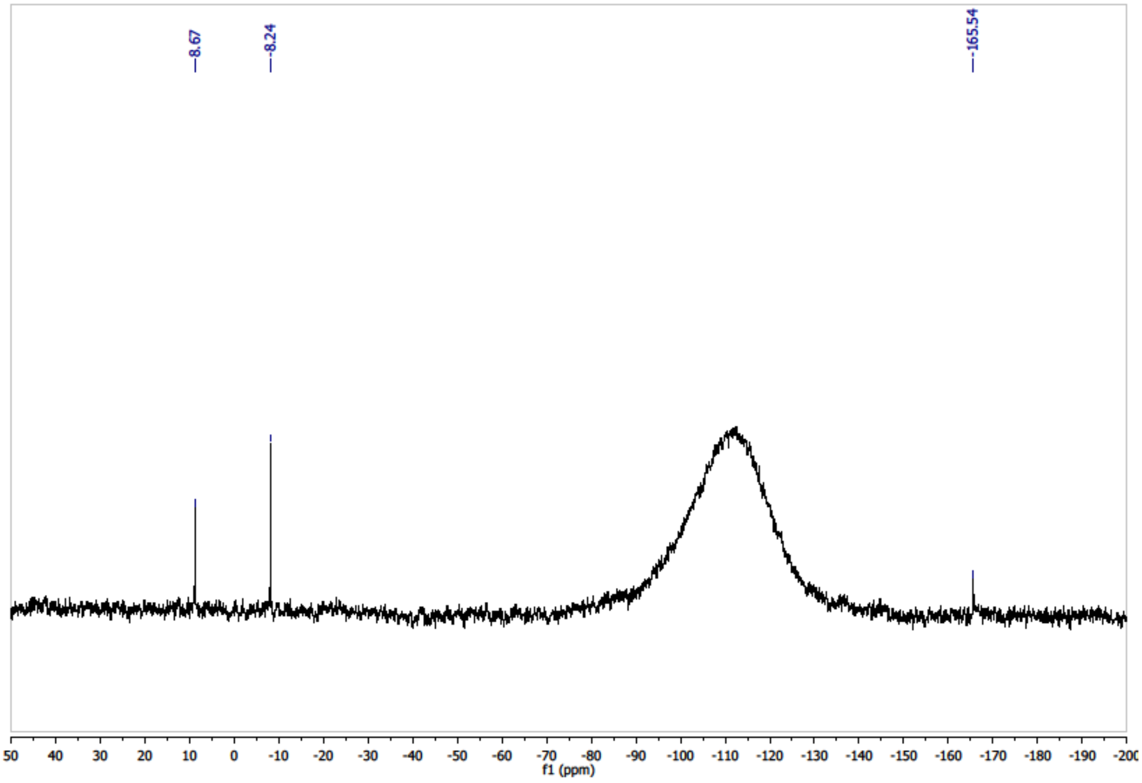

Figure S11. ^{29}Si inverse-gated NMR spectrum of **1b** in C_6D_6

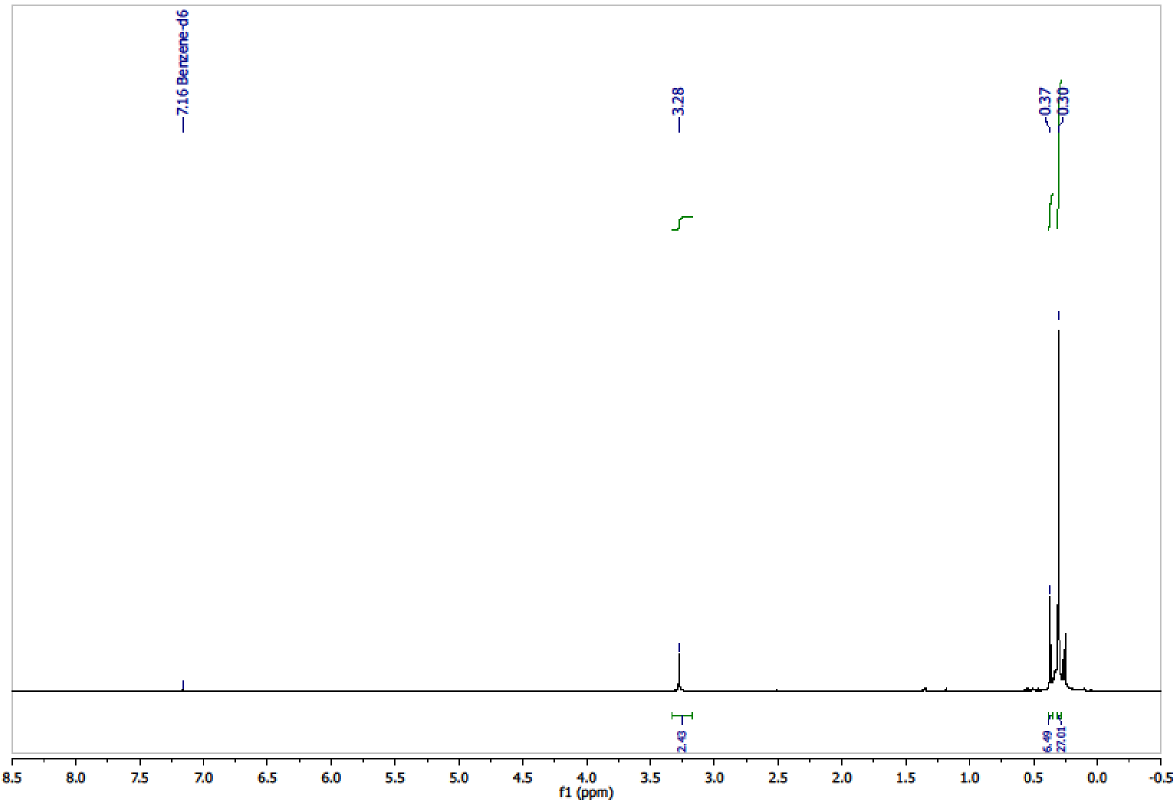

Figure S12. ^{1}H NMR spectrum of **2** in C_6D_6

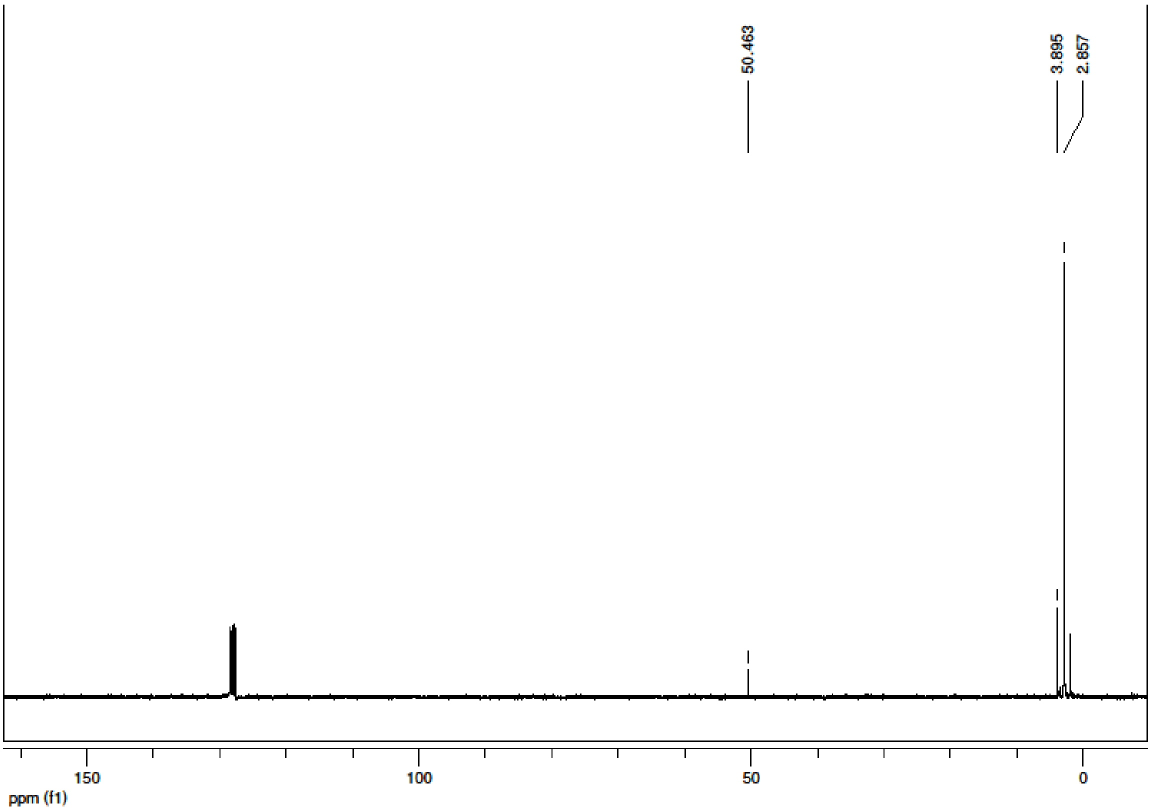

Figure S13. ^{13}C NMR spectrum of **2** in C_6D_6

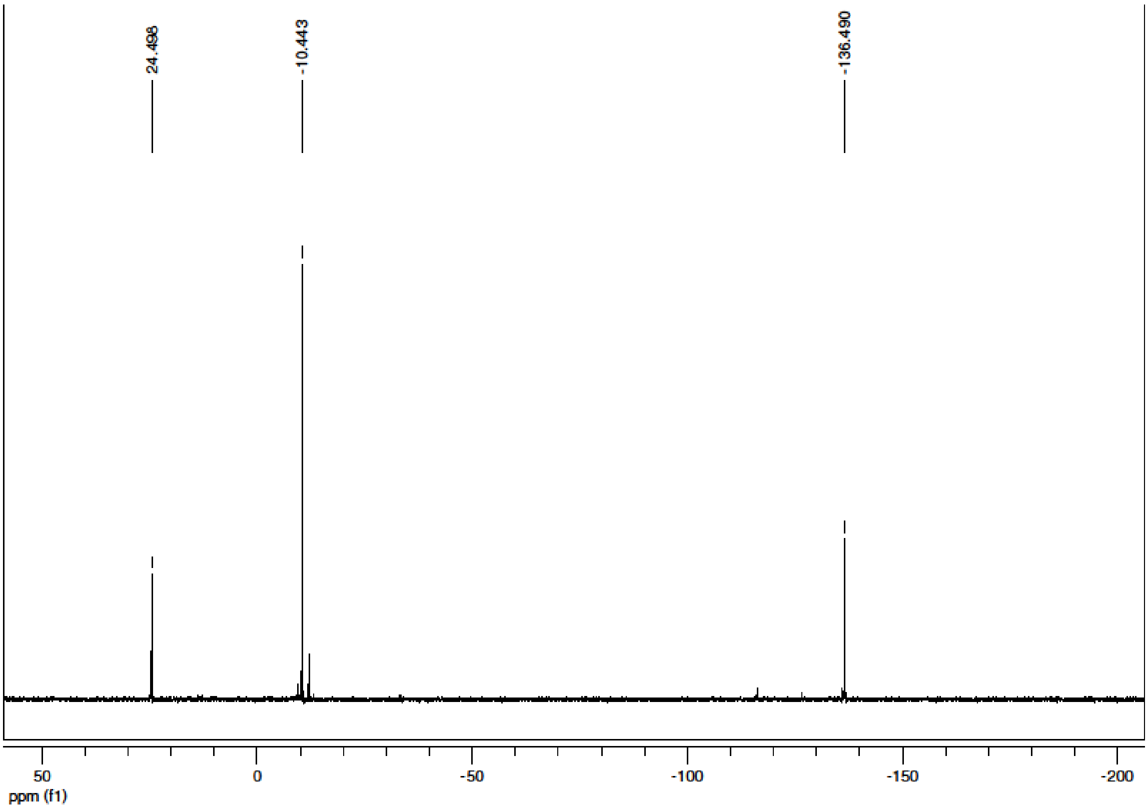

Figure S14. ^{29}Si INEPT NMR spectrum of **2** in C_6D_6

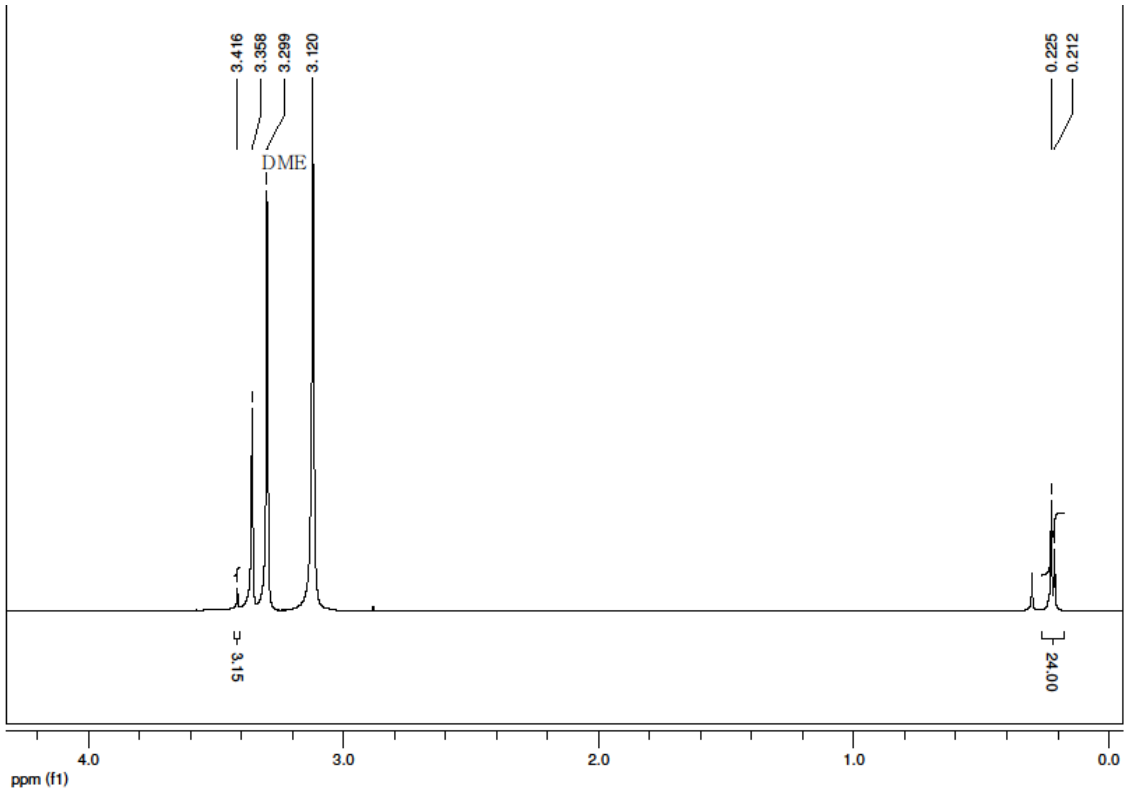

Figure S15. ^{1}H NMR spectrum of **2a** in C_6D_6

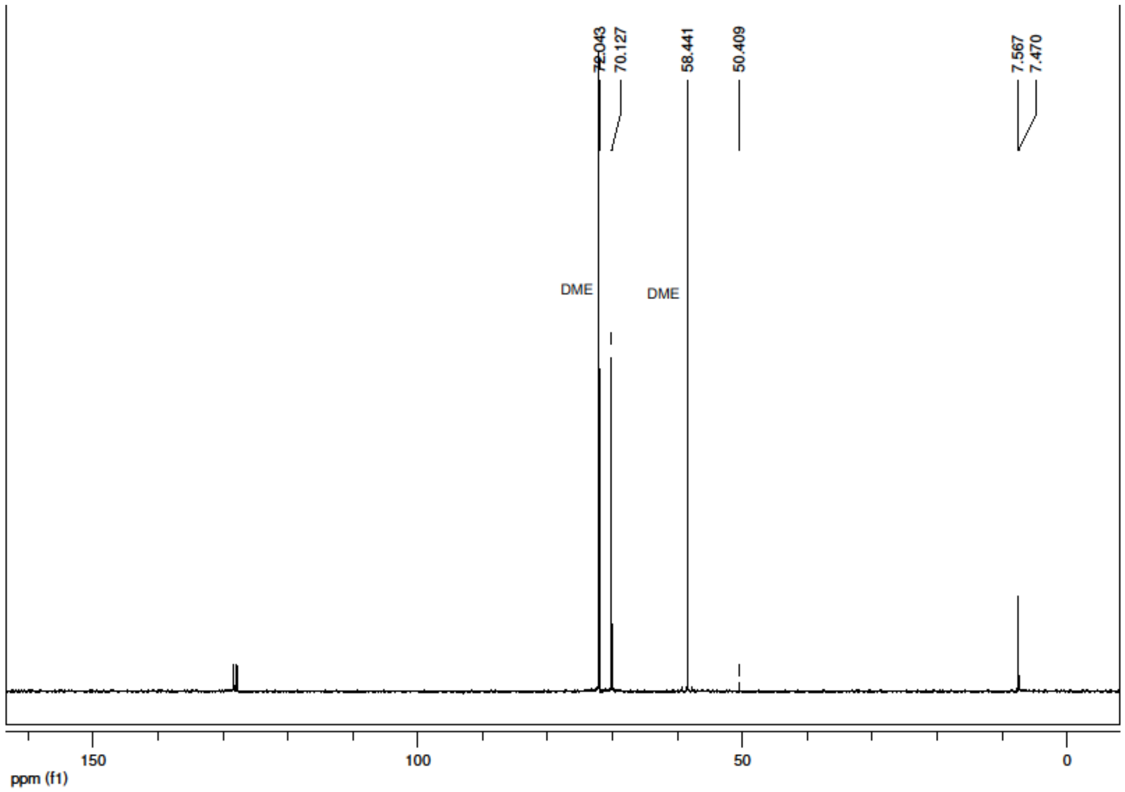

Figure S16. ^{13}C NMR spectrum of **2a** in C_6D_6

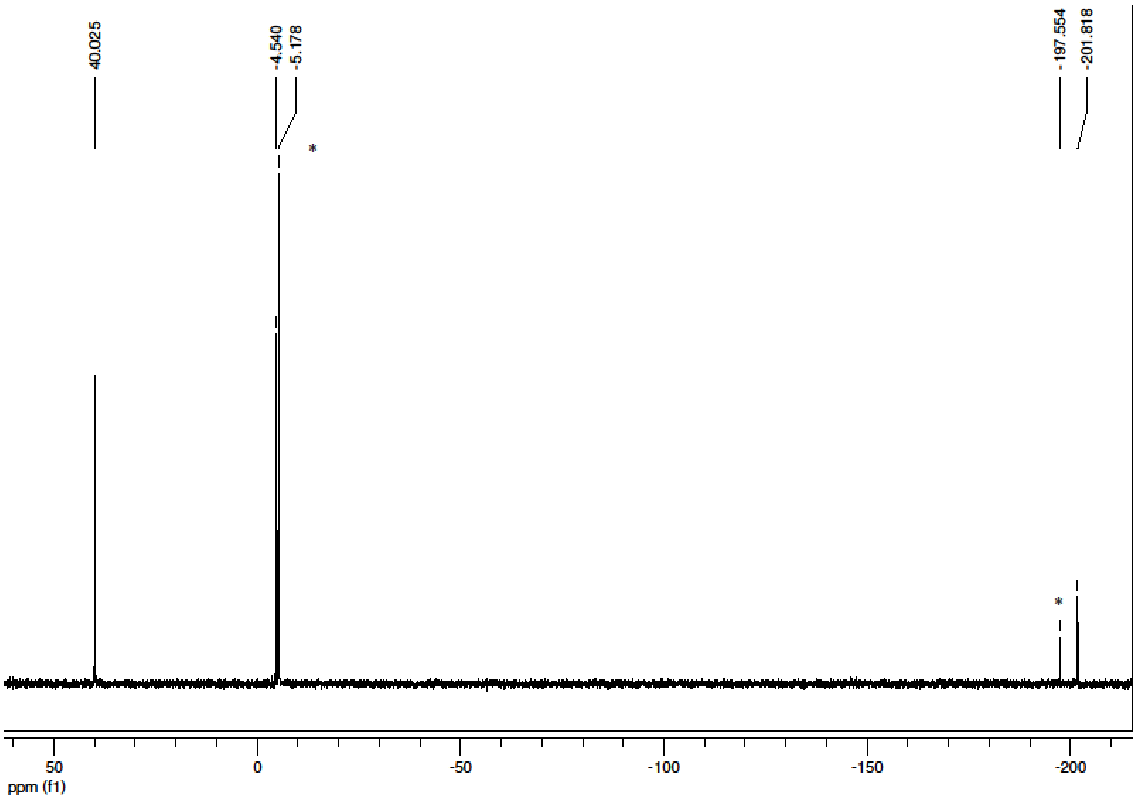

Figure S17. ^{29}Si NMR spectrum of **2a** in C_6D_6 (* = $(Me_3Si)_3SiK$)

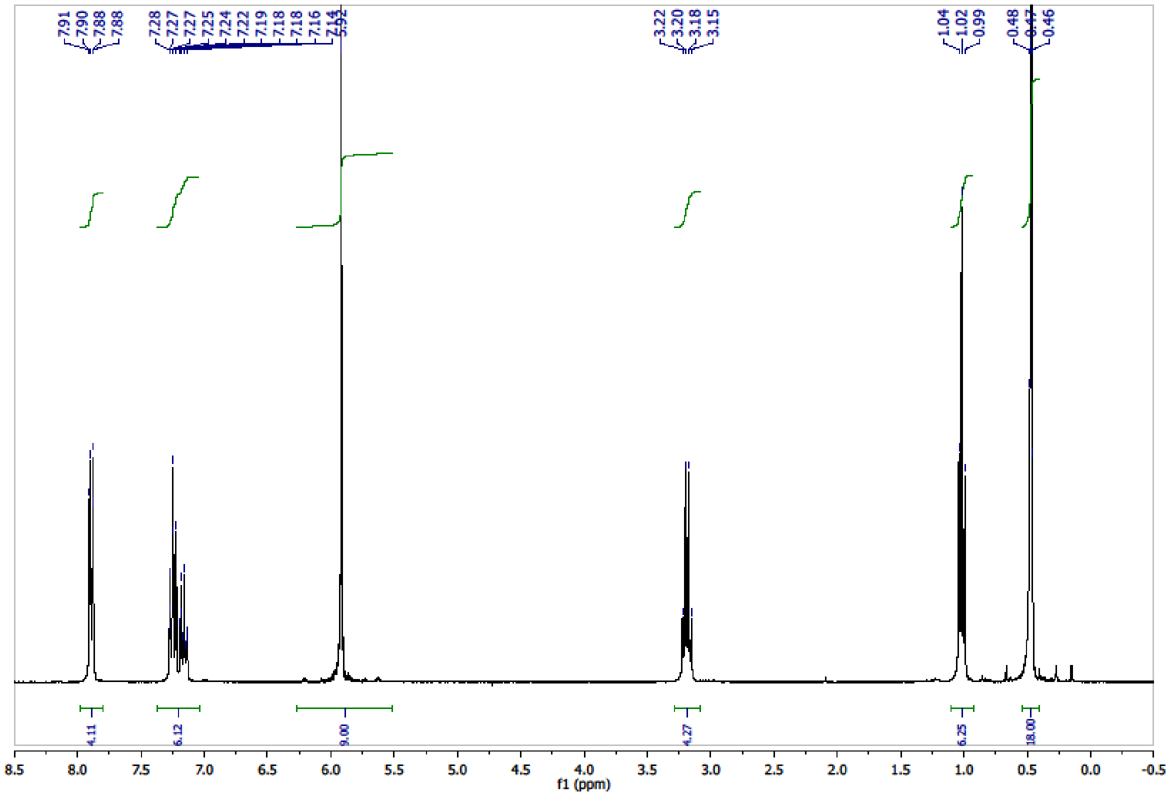

Figure S18. ^{1}H NMR spectrum of **3a** in C_6D_6

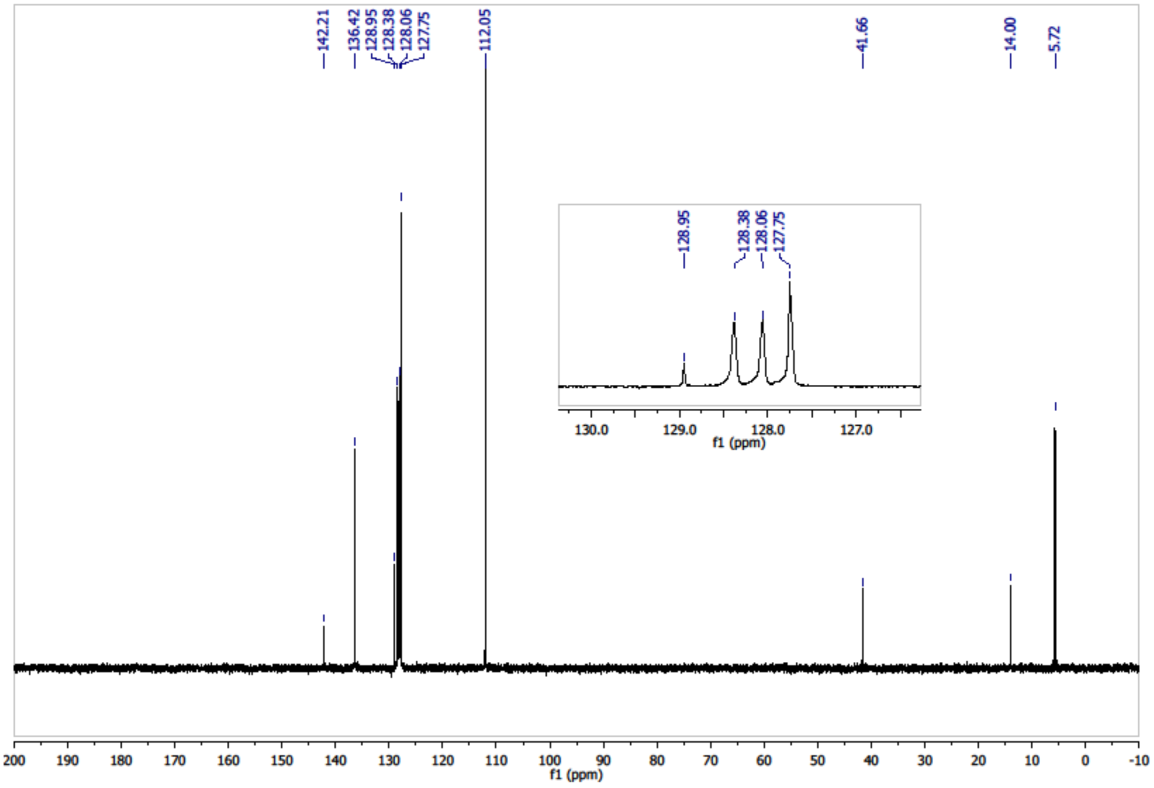

Figure S19. ^{13}C NMR spectrum of **3a** in C_6D_6

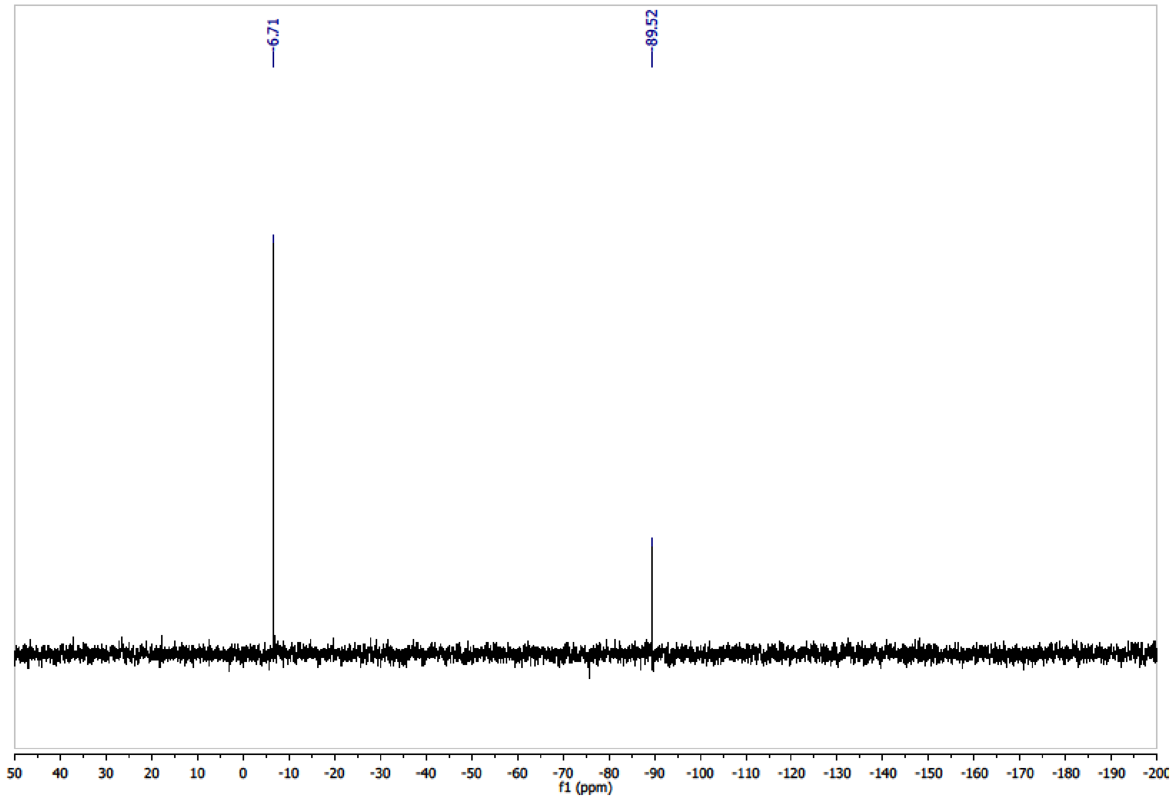

Figure S20. ^{29}Si INEPT NMR spectrum of **3a** in C_6D_6

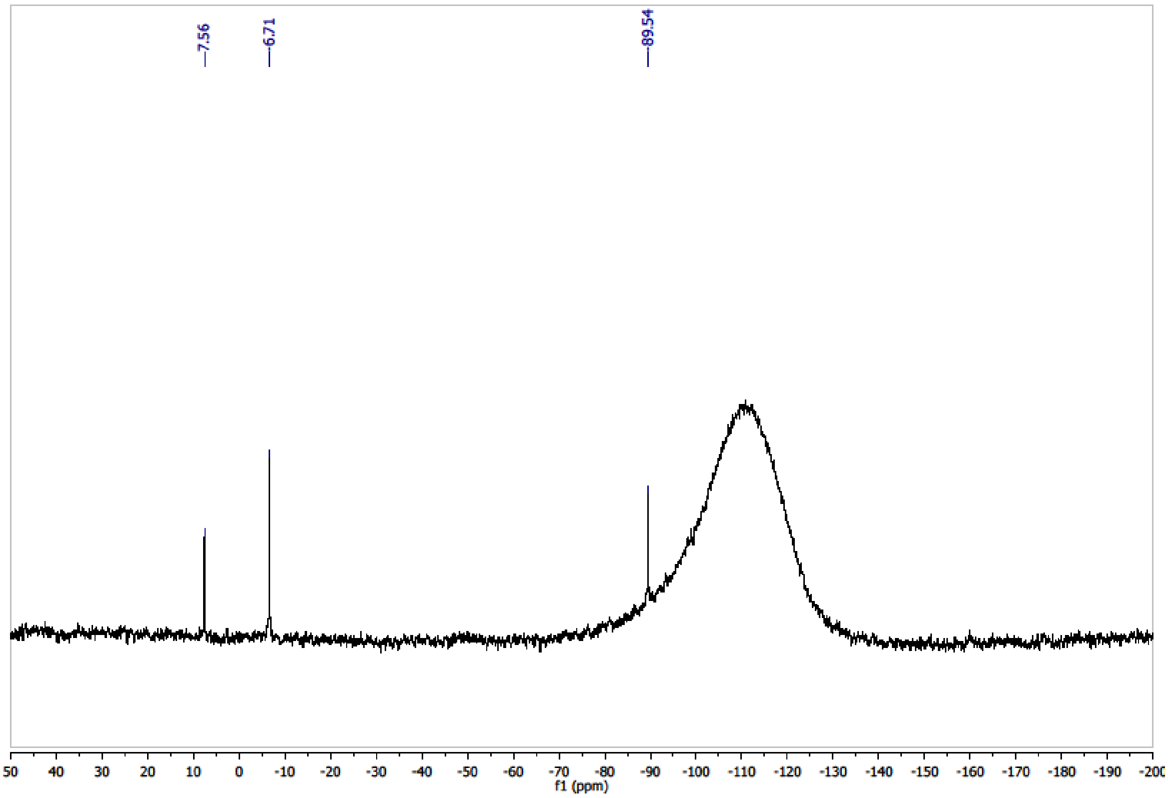

Figure S21. ^{29}Si inverse-gated NMR spectrum of **3a** in C_6D_6

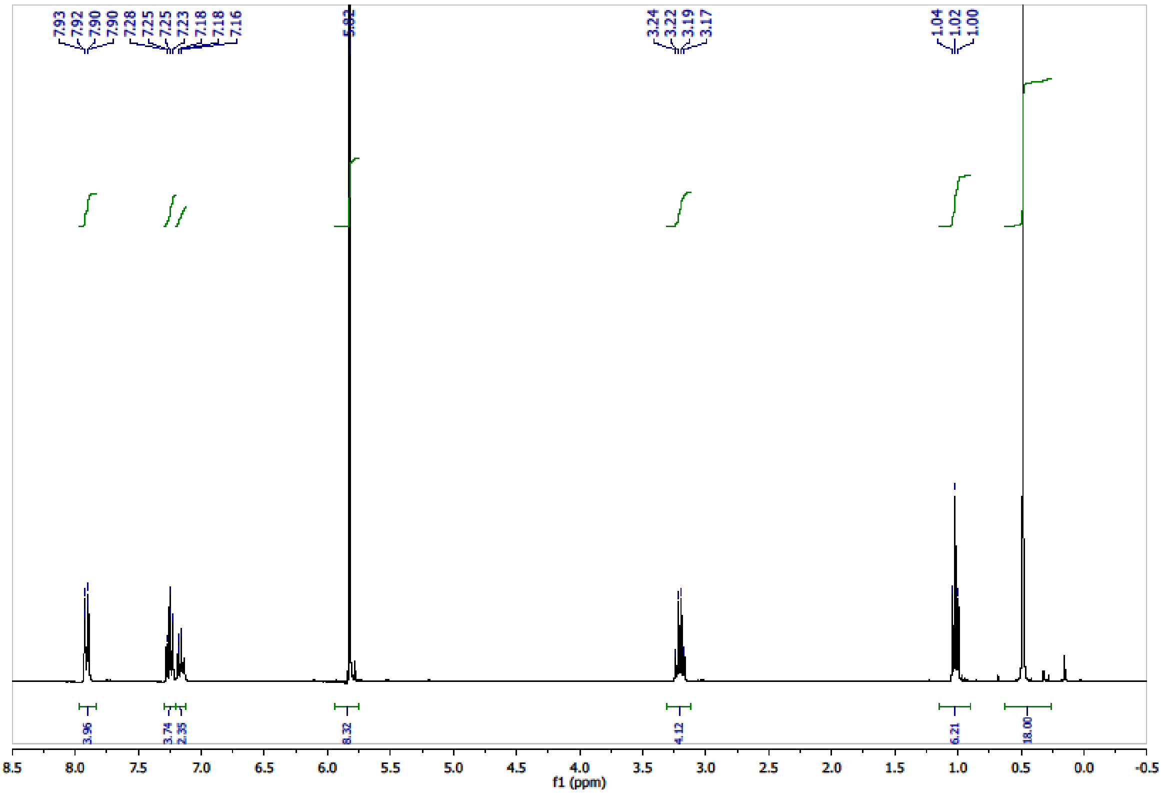

Figure S22. ^{1}H NMR spectrum of **3b** in C_6D_6

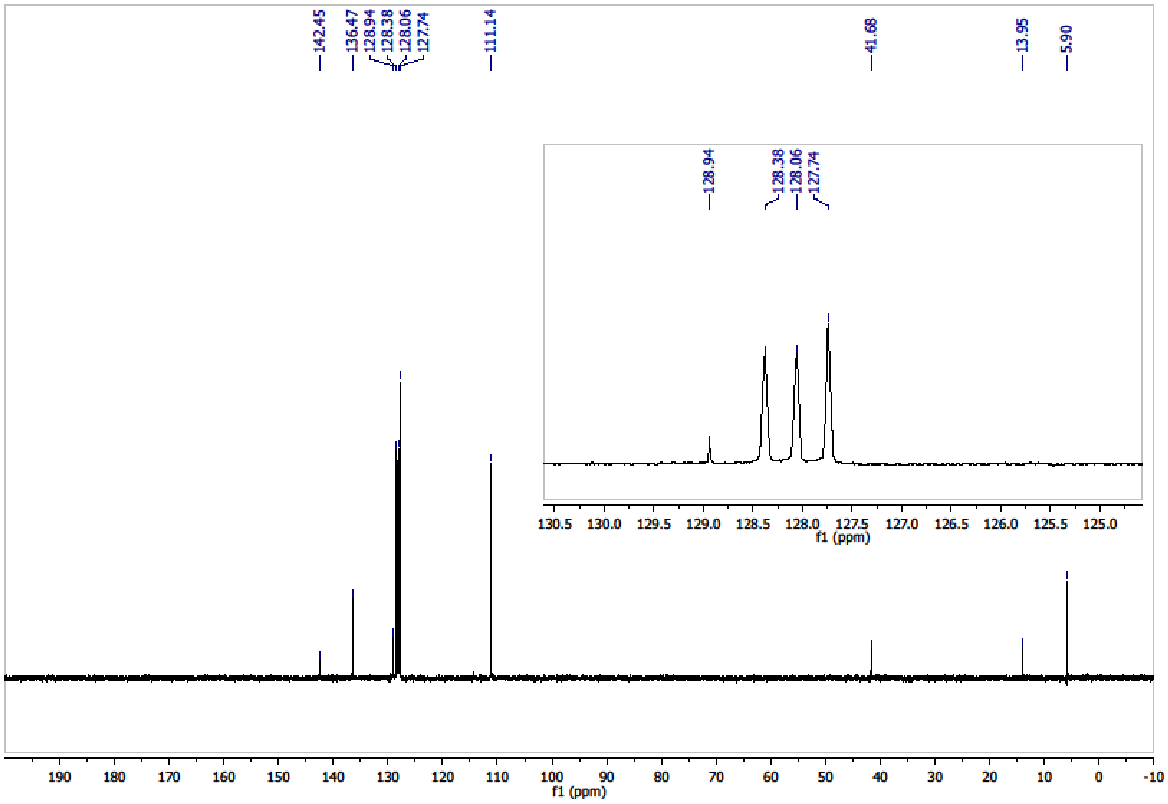

Figure S23. ^{13}C NMR spectrum of **3b** in C_6D_6

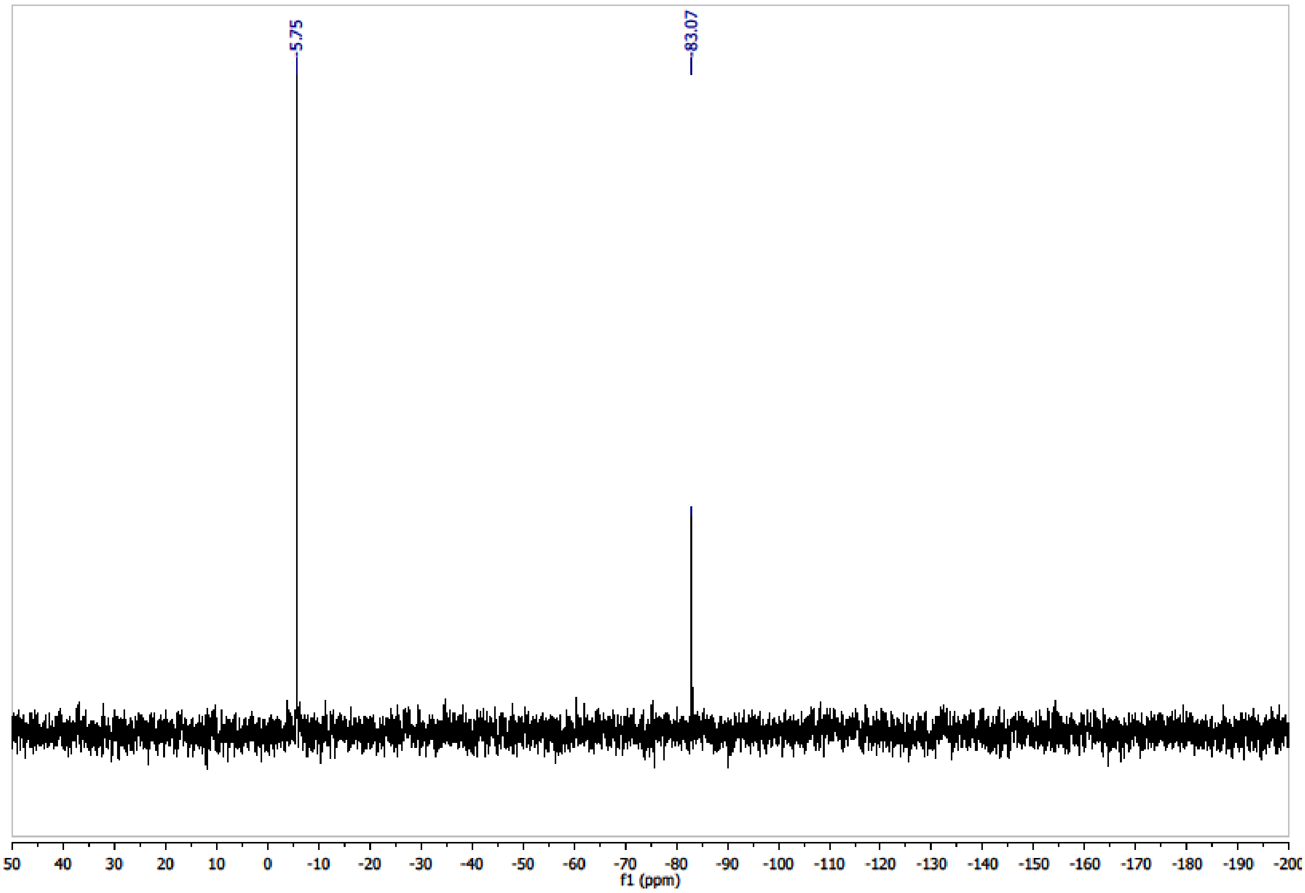

Figure S24. ^{29}Si INEPT NMR spectrum of **3b** in C_6D_6

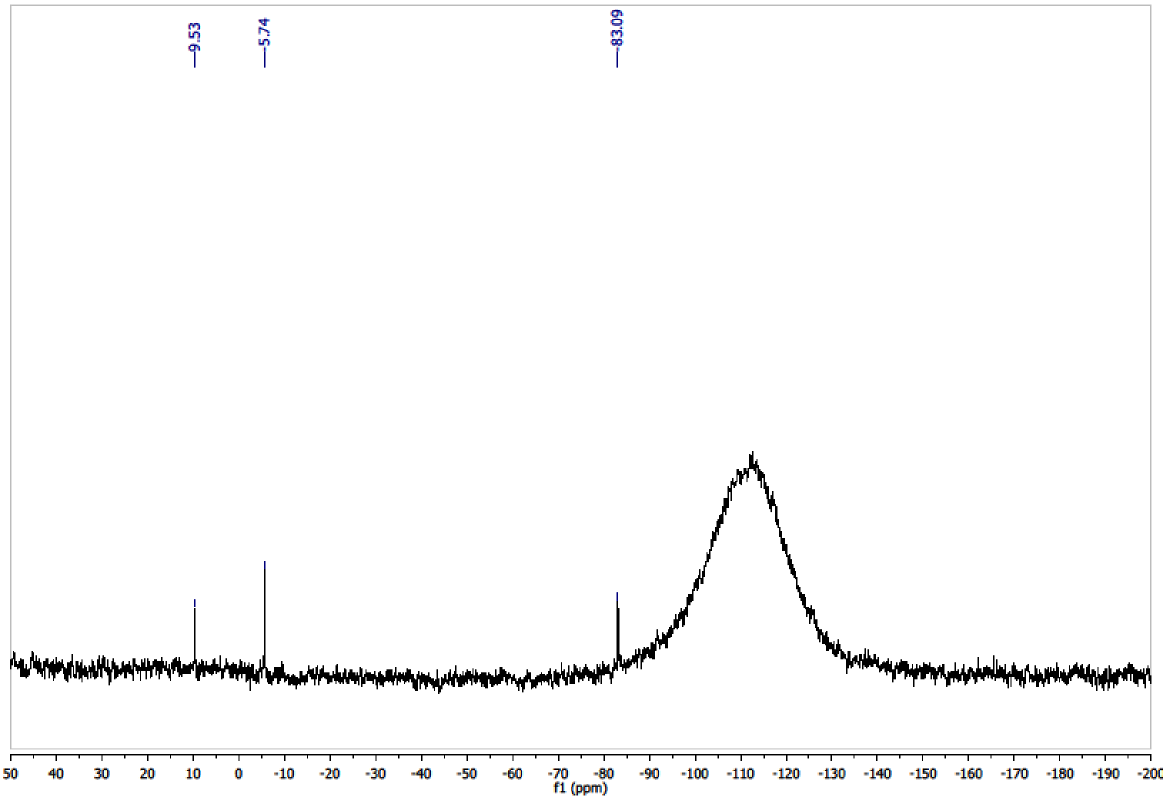

Figure S25. ^{29}Si inverse-gated NMR spectrum of **3b** in C_6D_6

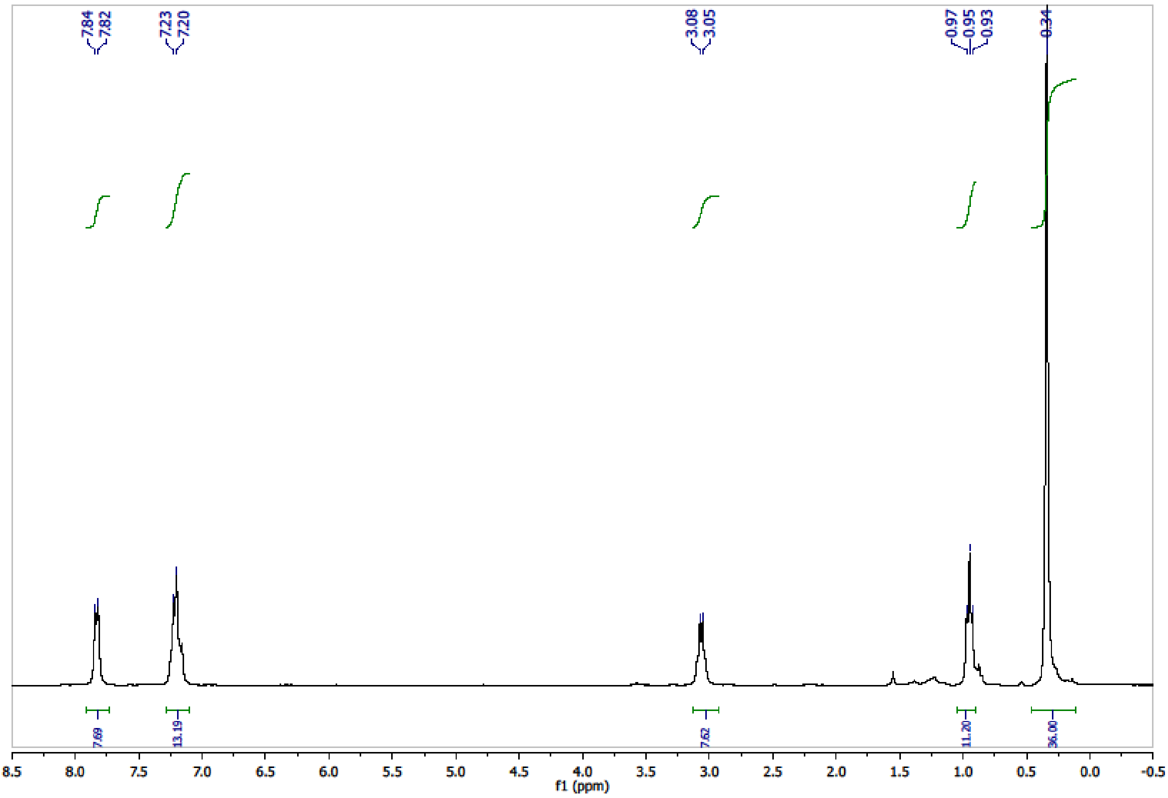

Figure S26. ^{1}H NMR spectrum of **4** in C_6D_6

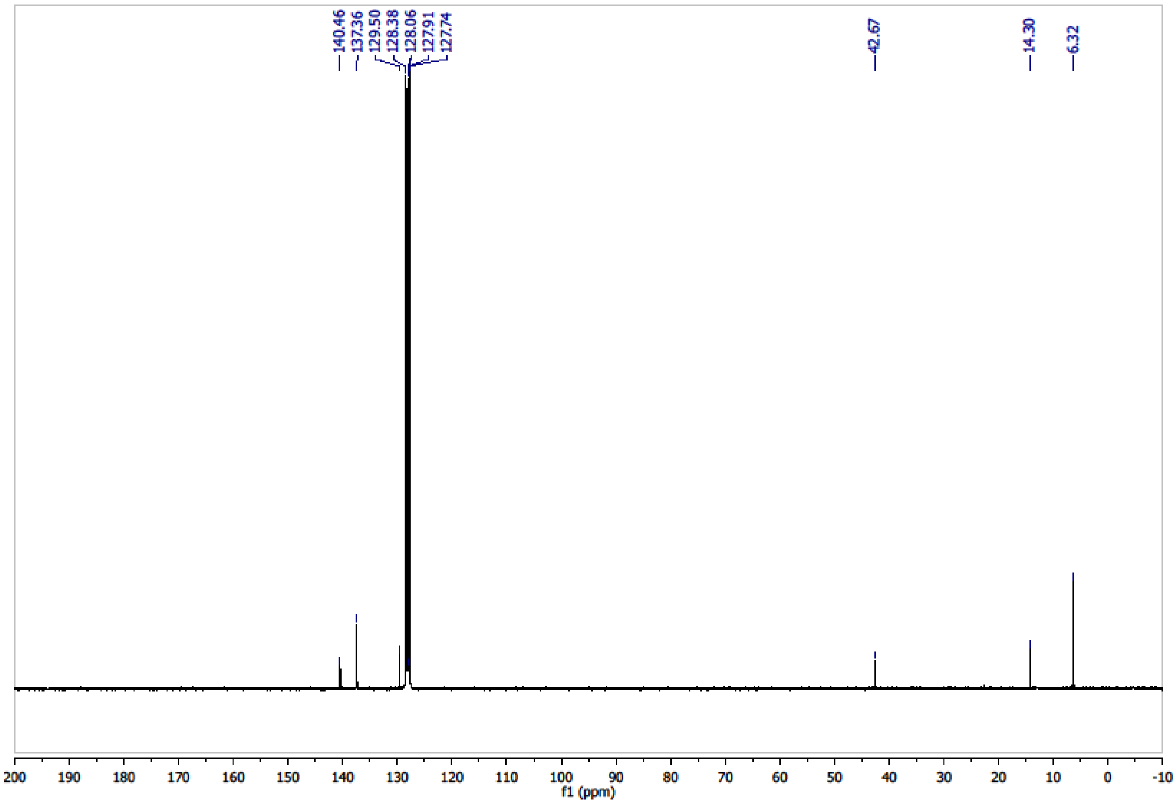

Figure S27. ^{13}C NMR spectrum of **4** in C_6D_6

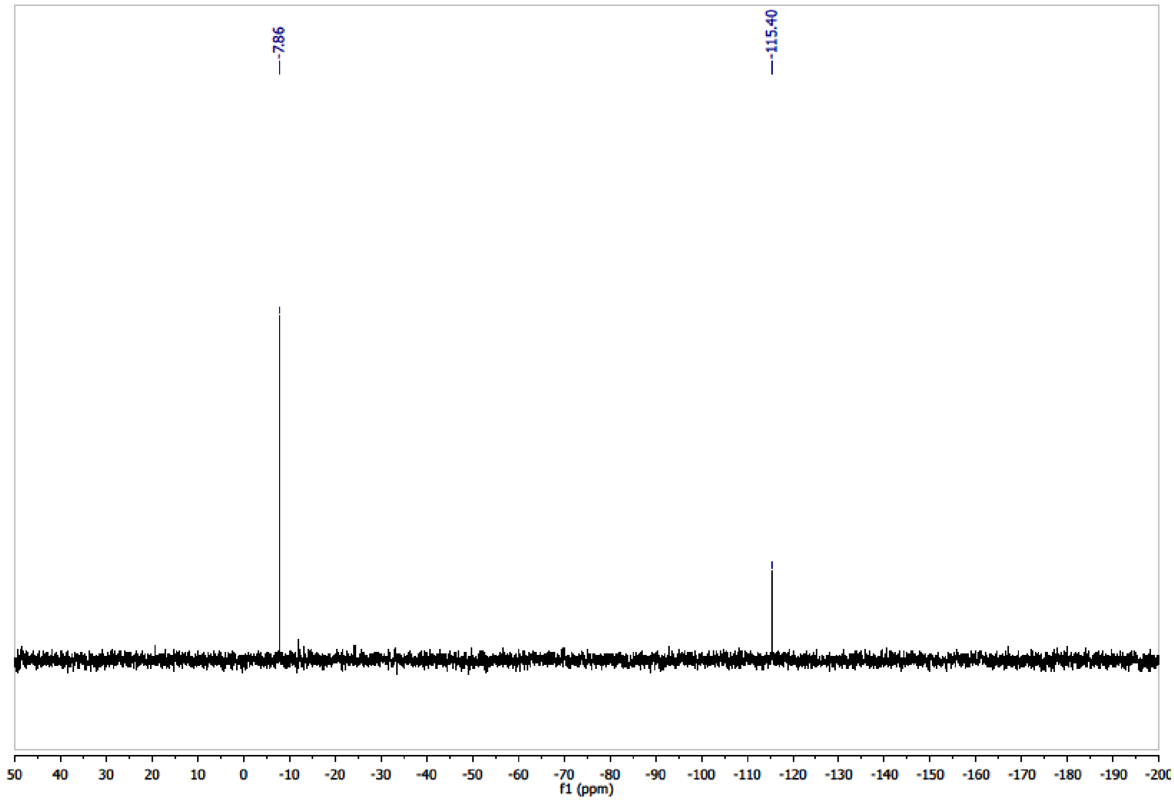

Figure S28. ^{29}Si INEPT NMR spectrum of **4** in C_6D_6

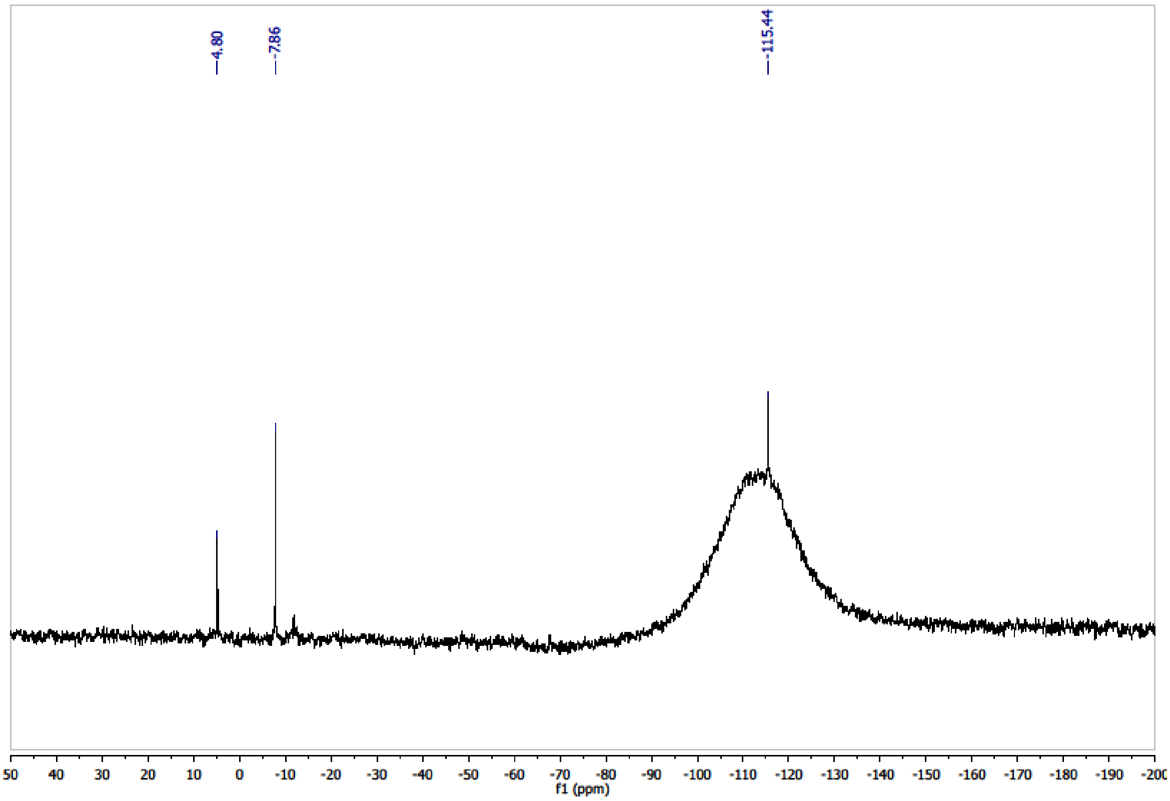

Figure S29. ^{29}Si inverse-gated NMR spectrum of **4** in C_6D_6

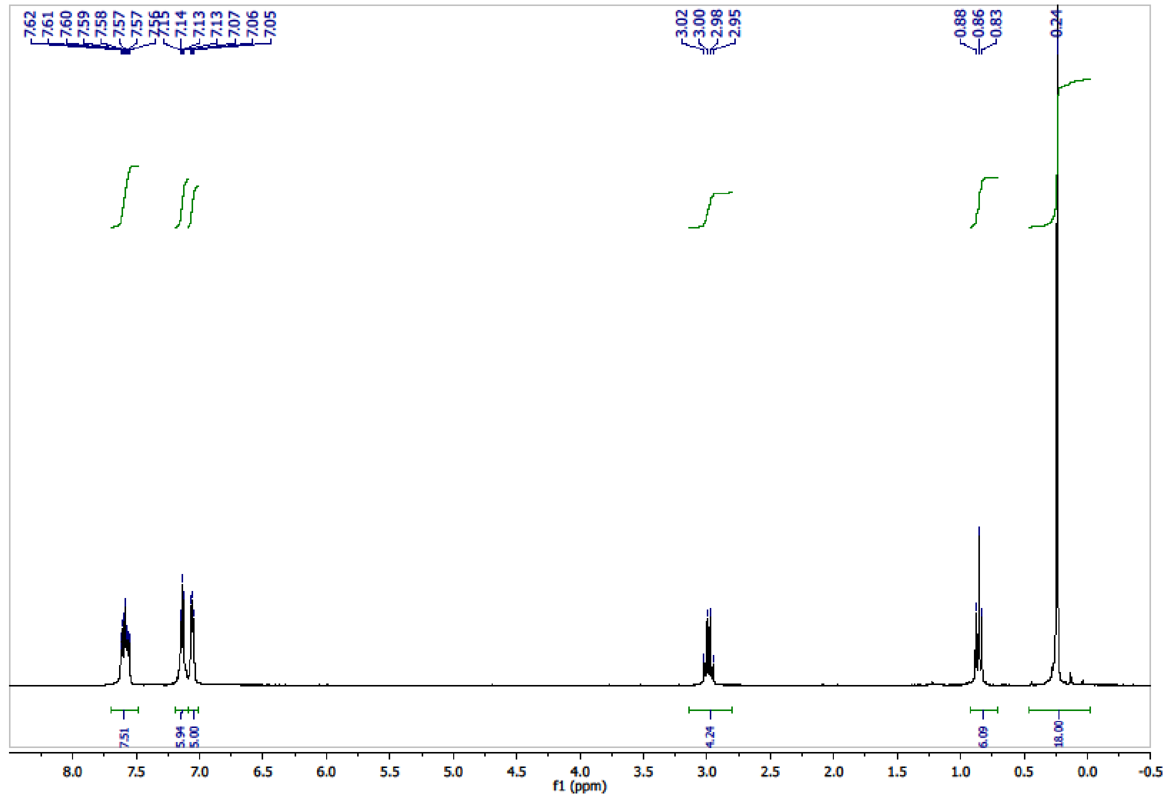

Figure S30. ^{1}H NMR spectrum of **5** in C_6D_6

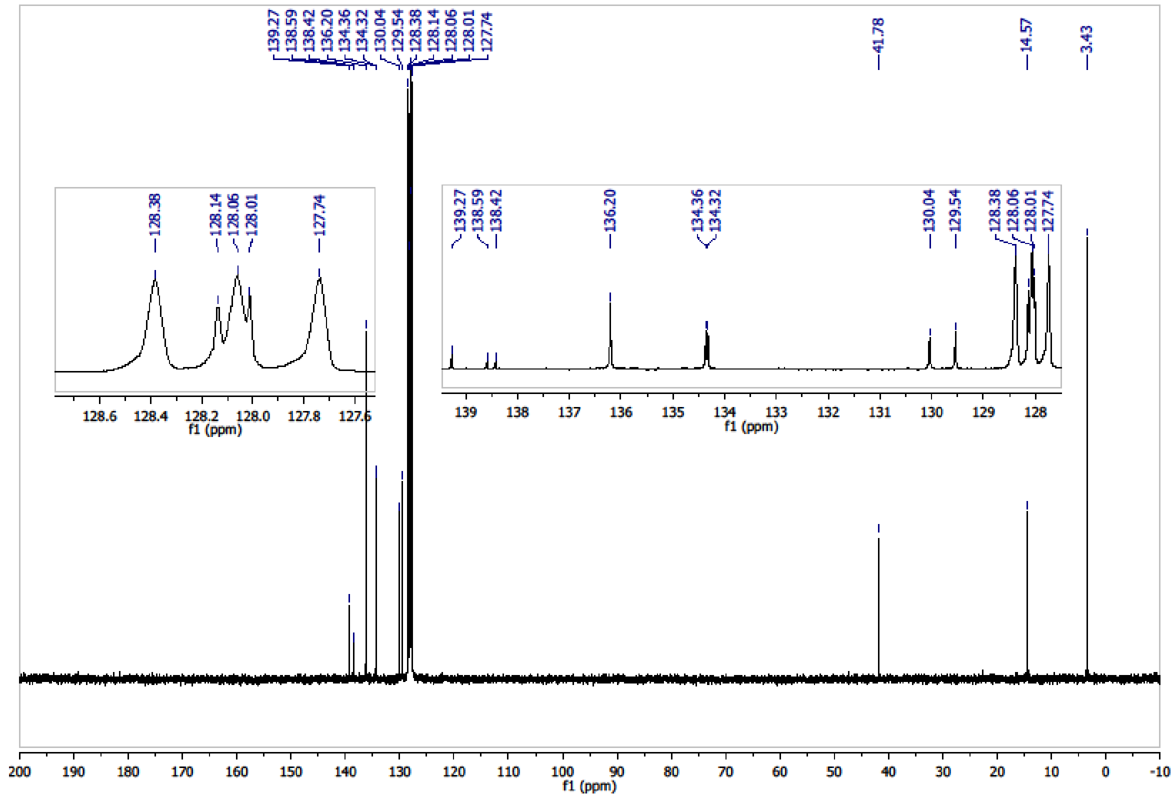

Figure S31. ^{13}C NMR spectrum of **5** in C_6D_6

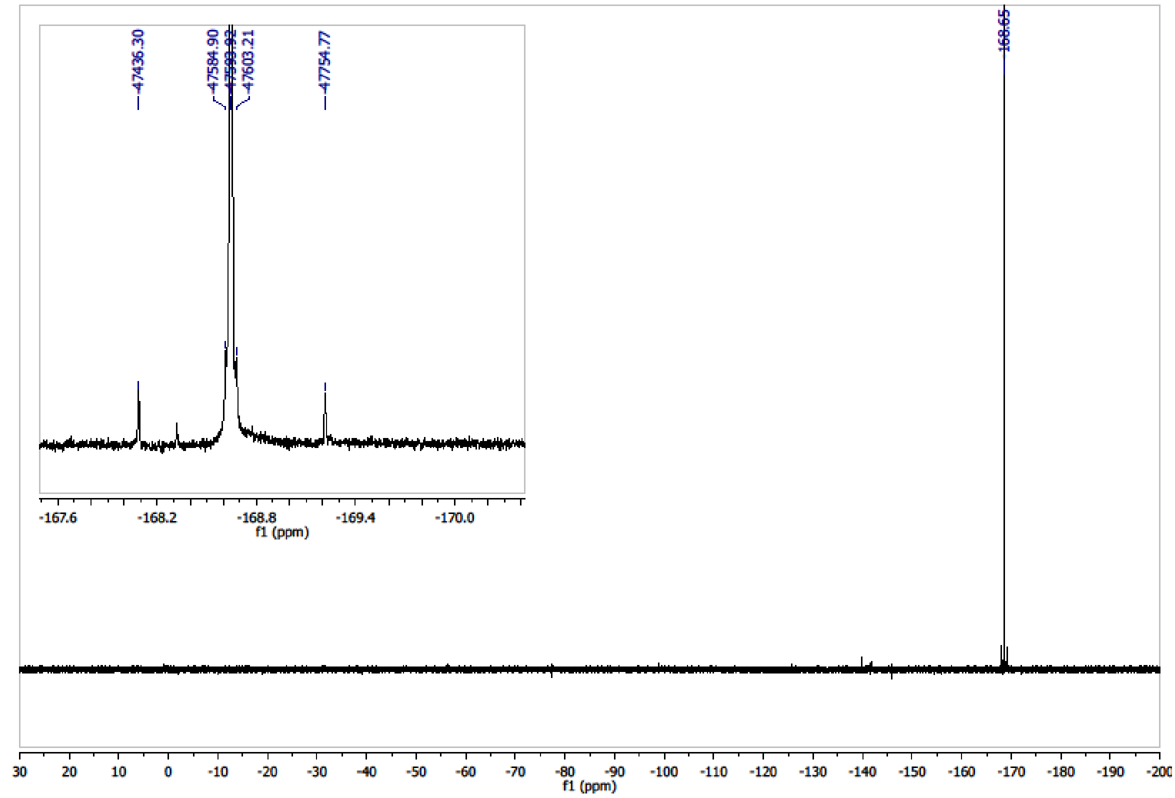

Figure S32. ^{19}F NMR spectrum of **5** in C_6D_6

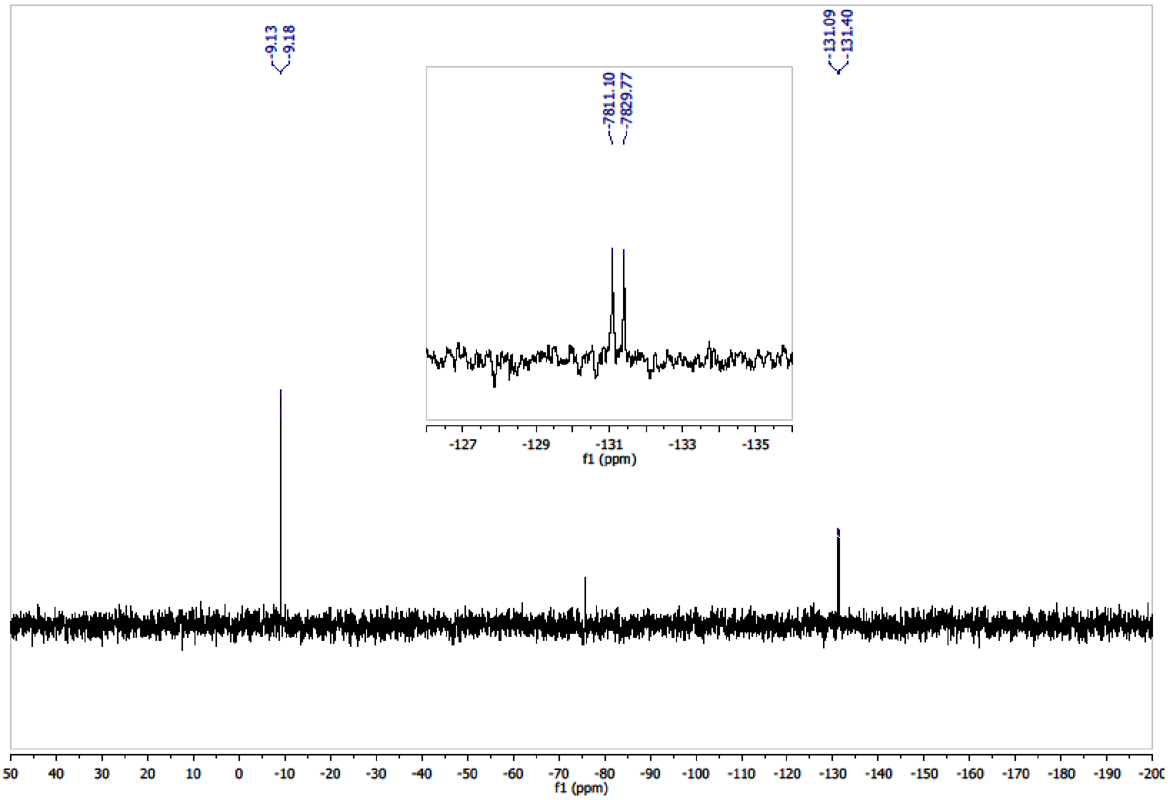

Figure S33. ^{29}Si INEPT NMR spectrum of **5** in C_6D_6

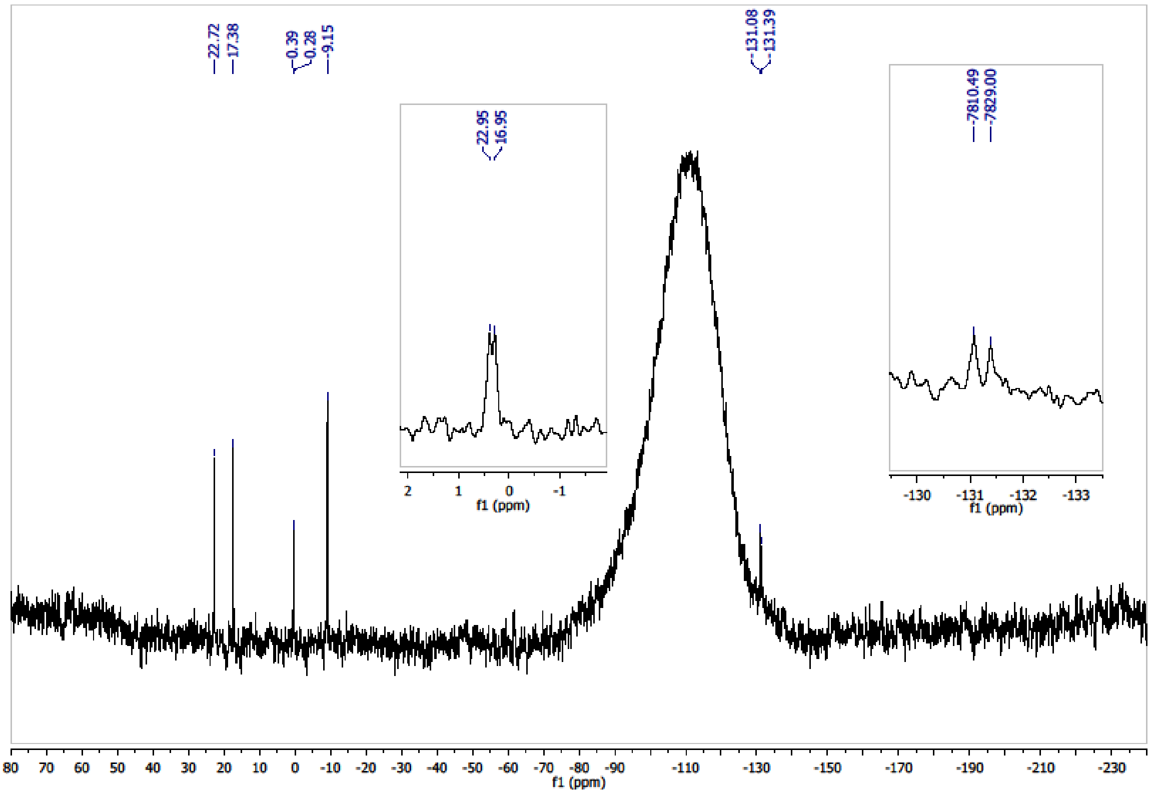

Figure S34. ^{29}Si inverse-gated NMR spectrum of **5** in C_6D_6

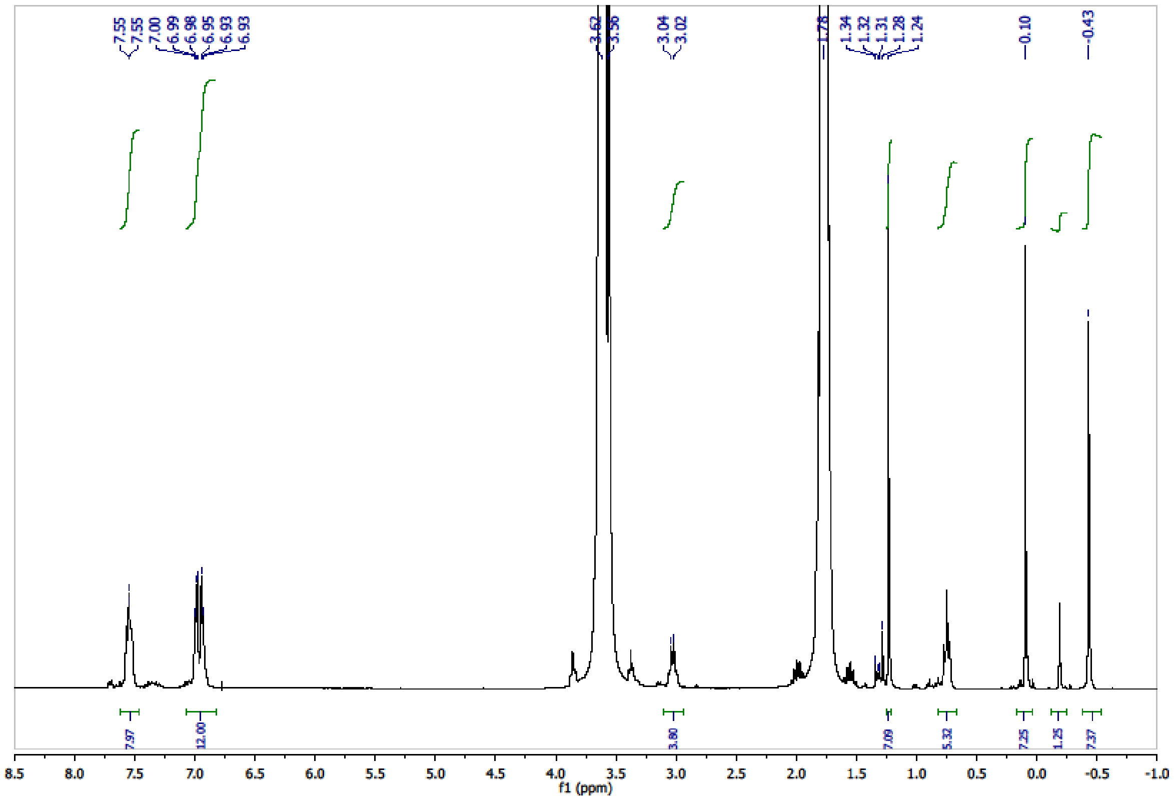

Figure S35. ^{1}H NMR spectrum of the reaction solution of **5a** in THF at −30 ℃

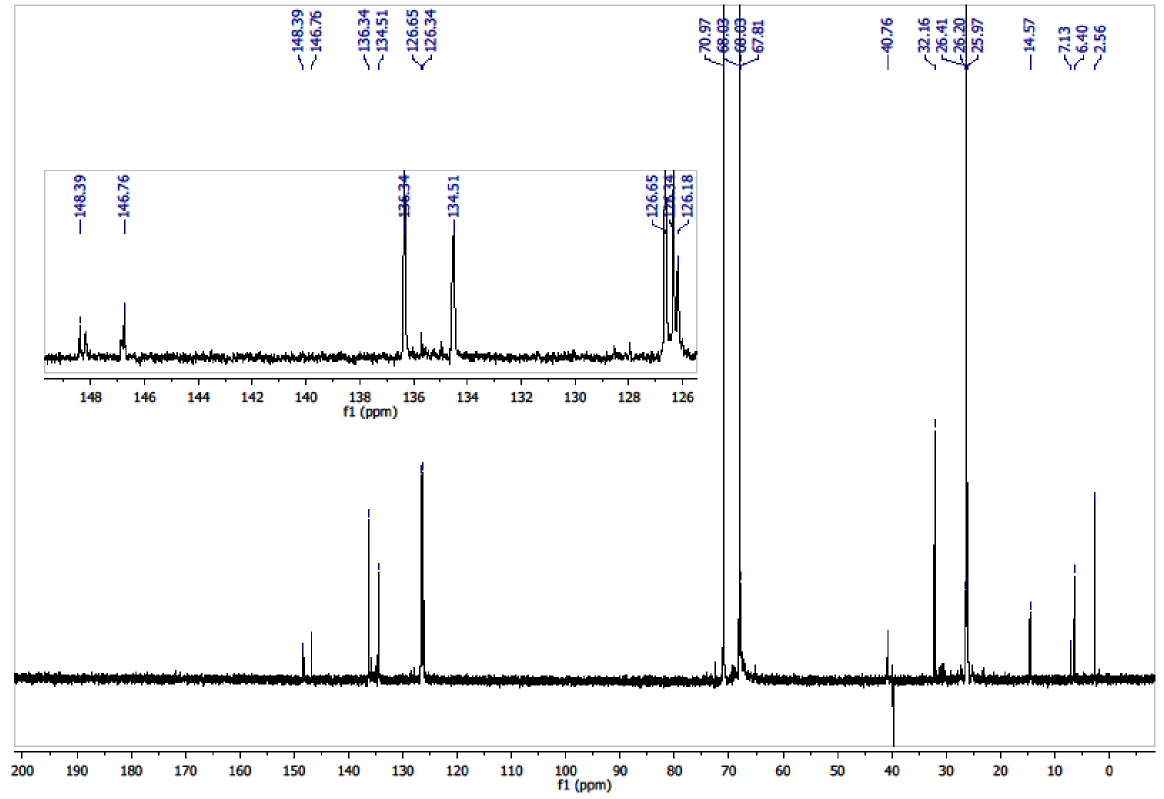

Figure S36. ^{13}C NMR spectrum of the reaction solution of **5a** in THF at −30 ℃

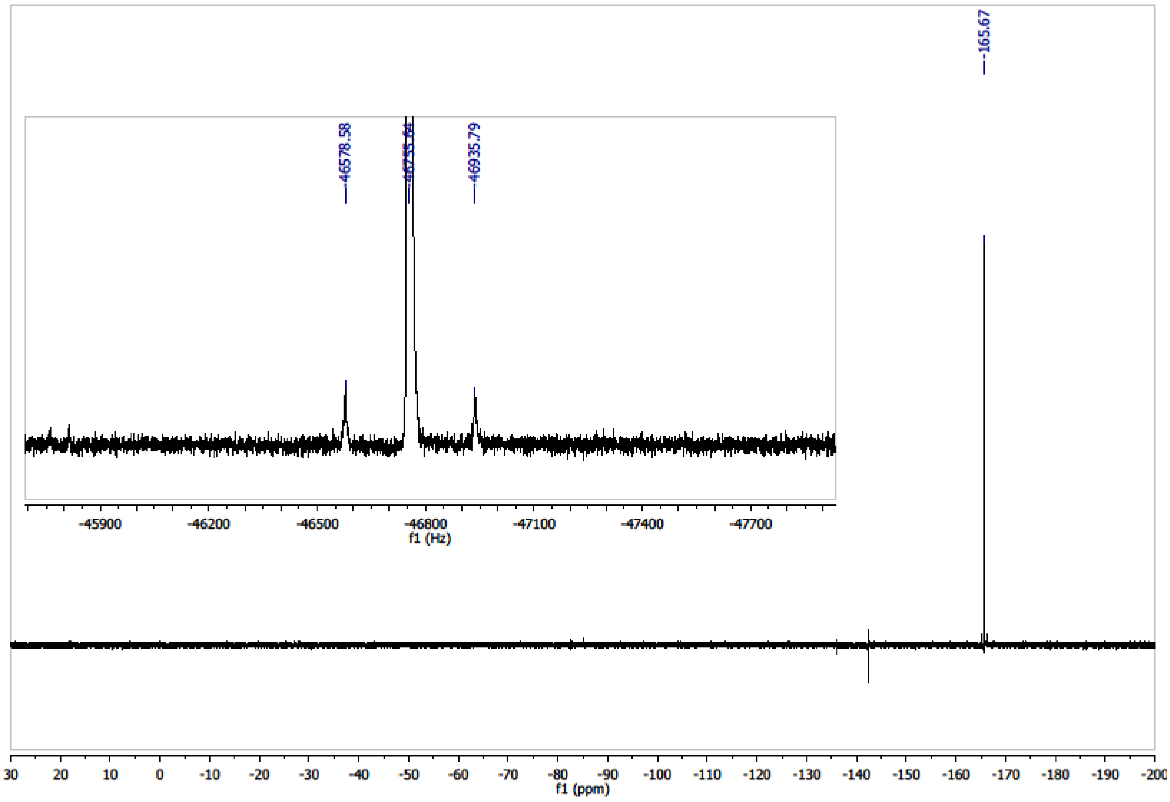

Figure S37. ^{19}F NMR spectrum of the reaction solution of **5a** in THF at −30 ℃

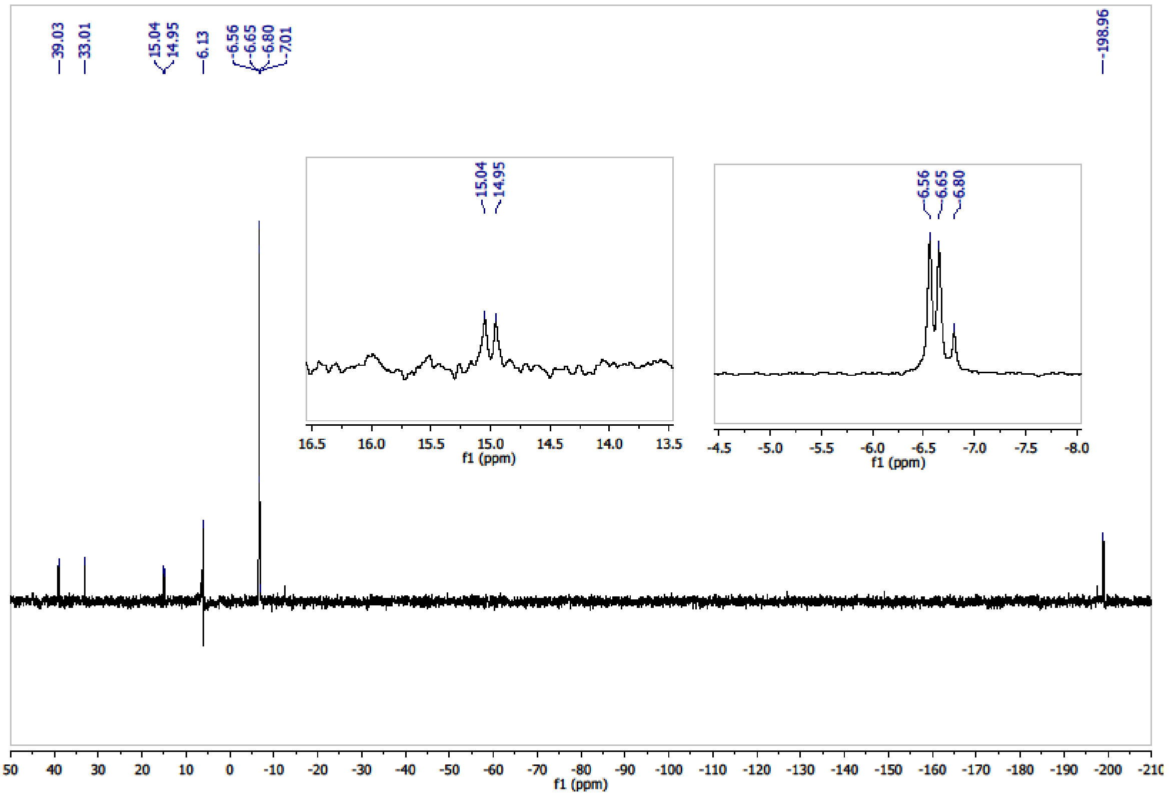

Figure S38. ^{29}Si INEPT NMR spectrum of the reaction solution of **5a** in THF at −30 ℃

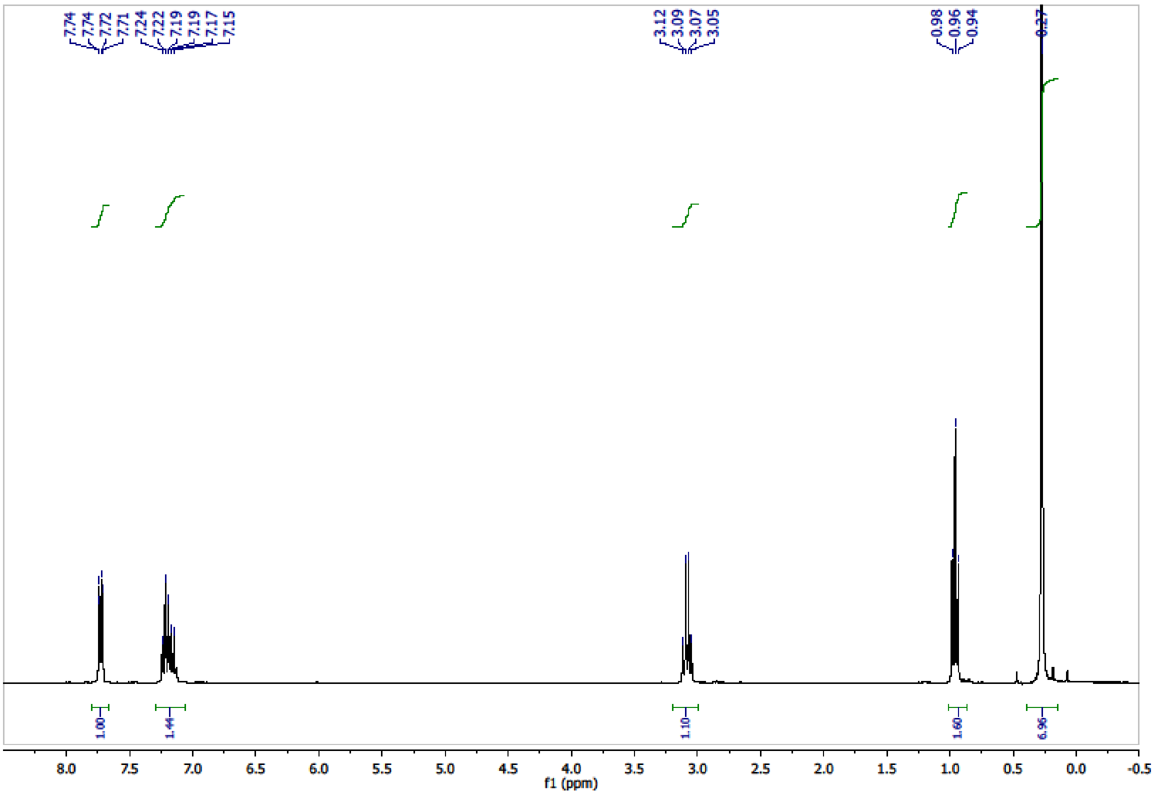

Figure S39. ^{1}H NMR spectrum of **6** in C_6D_6

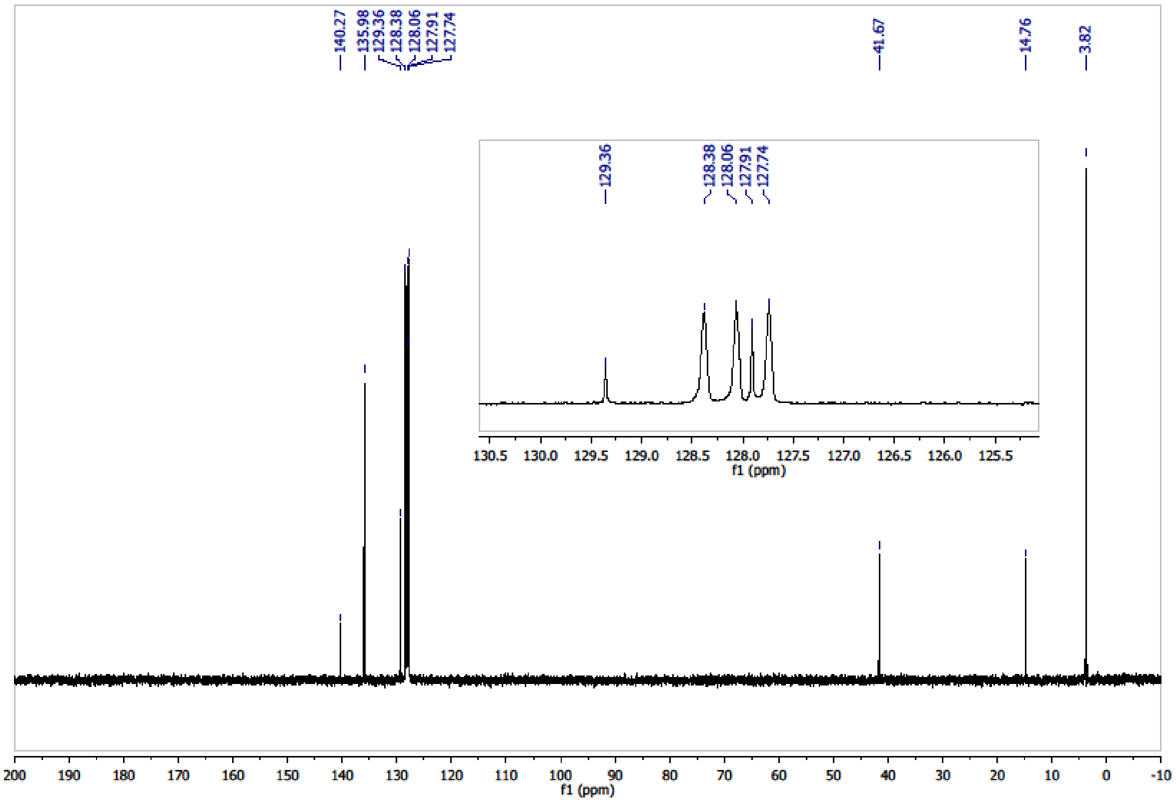

Figure S40. ^{13}C NMR spectrum of **6** in C_6D_6

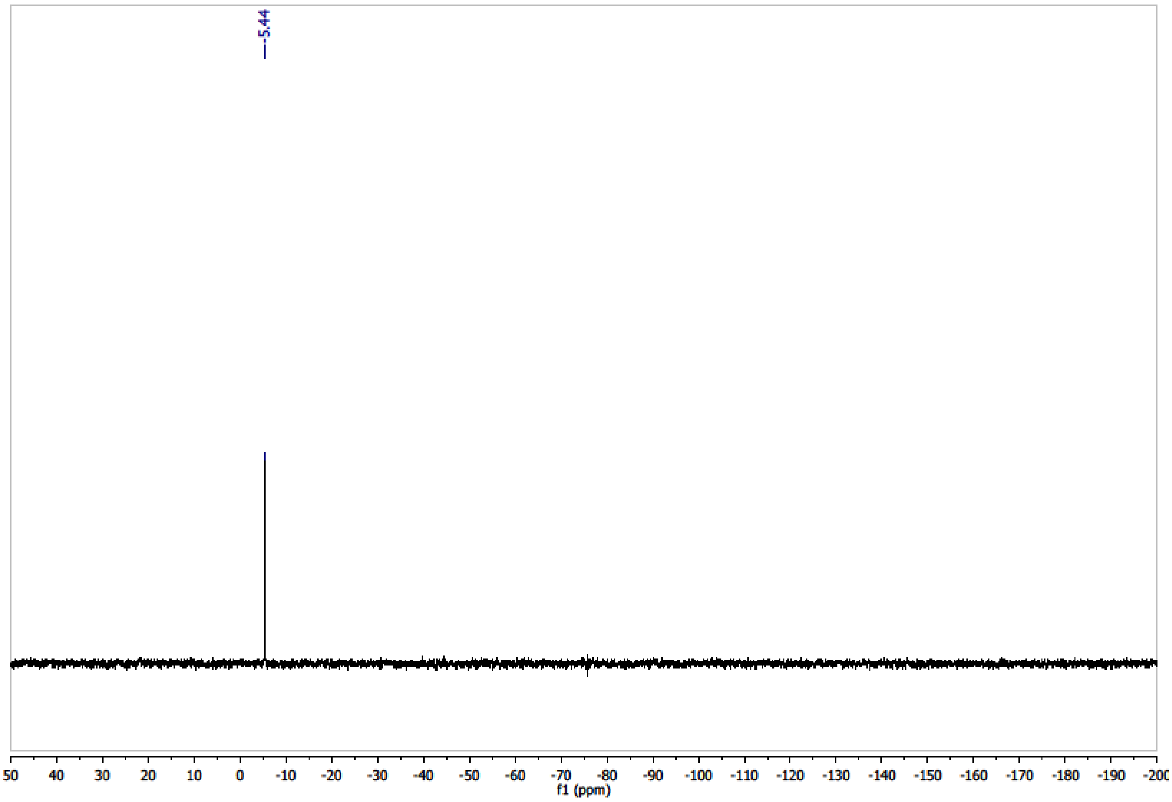

Figure S41. ^{29}Si INEPT NMR spectrum of **6** in C_6D_6

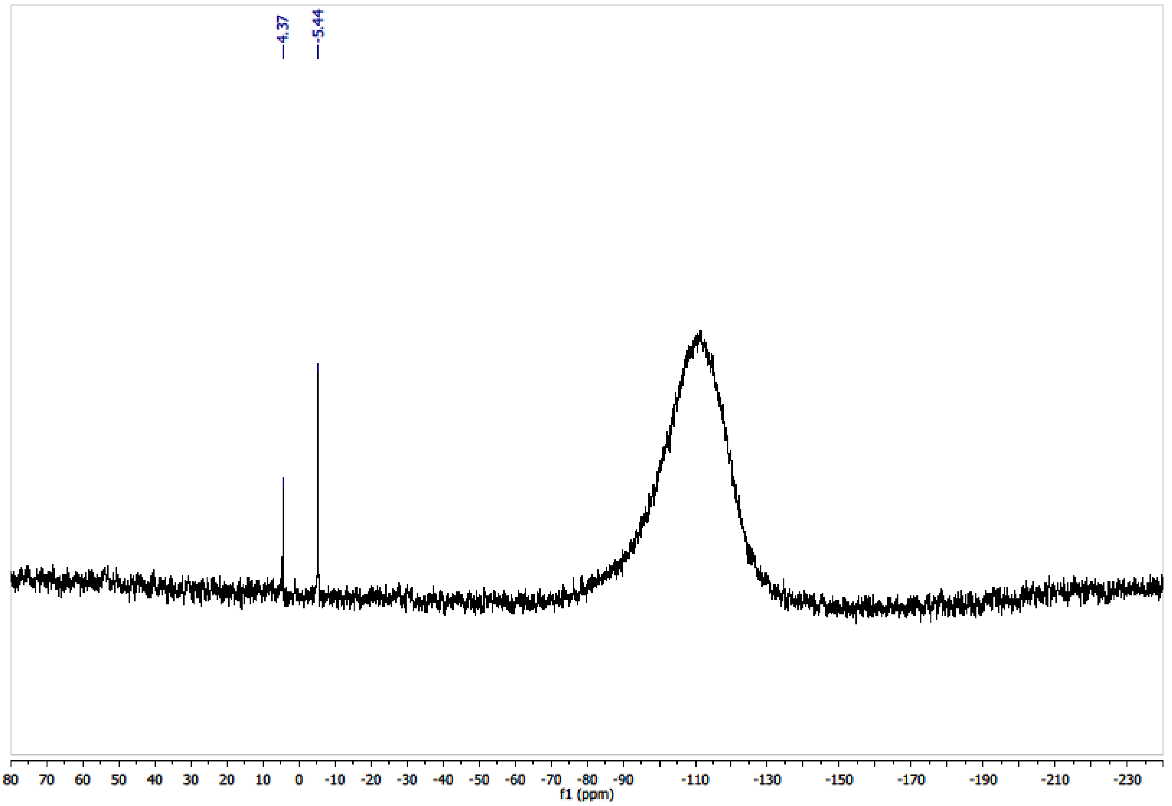

Figure S42. ^{29}Si inverse-gated NMR spectrum of **6** in C_6D_6

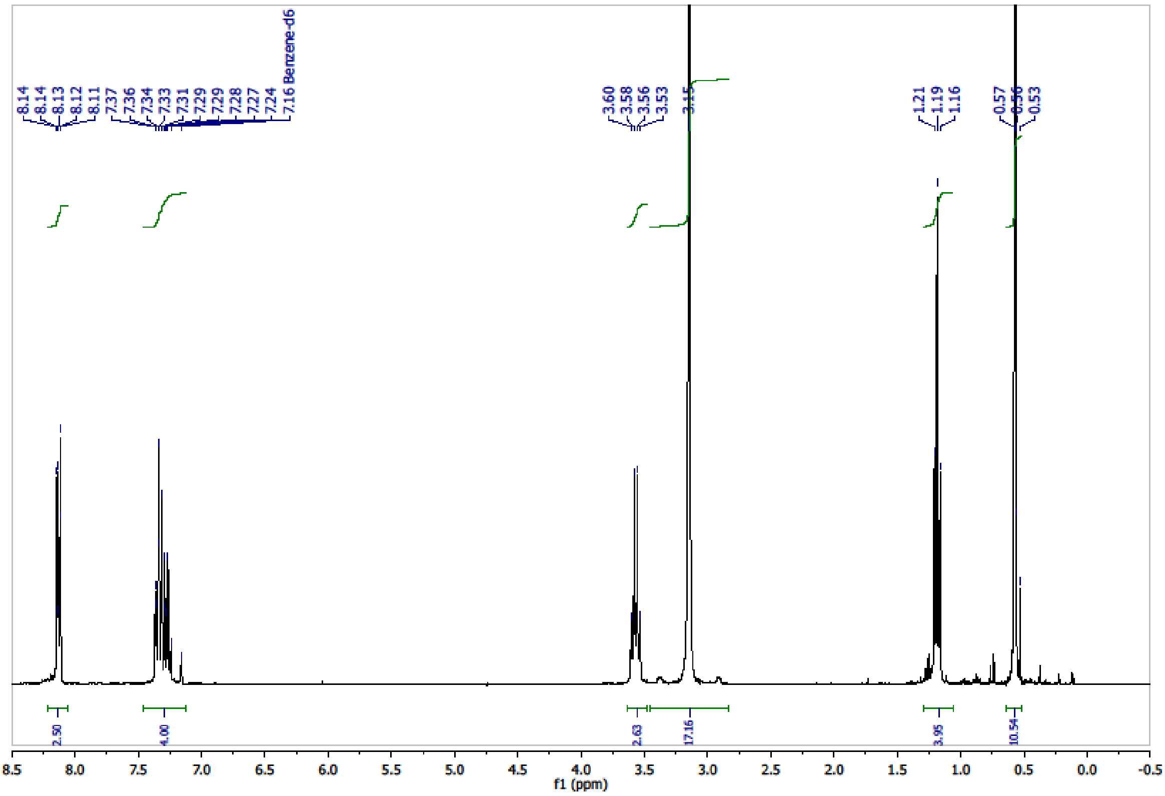

Figure S43. ^{1}H NMR spectrum of **6a** in C_6D_6

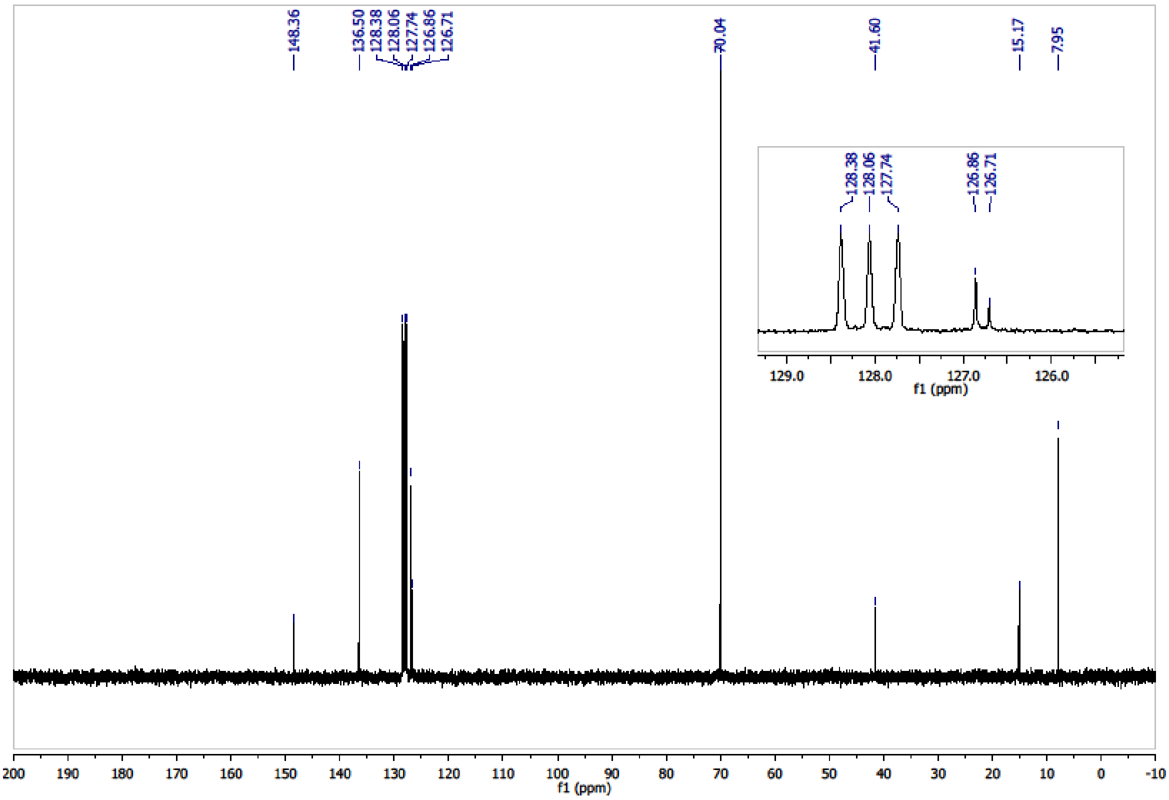

Figure S44. ^{13}C NMR spectrum of **6a** in C_6D_6

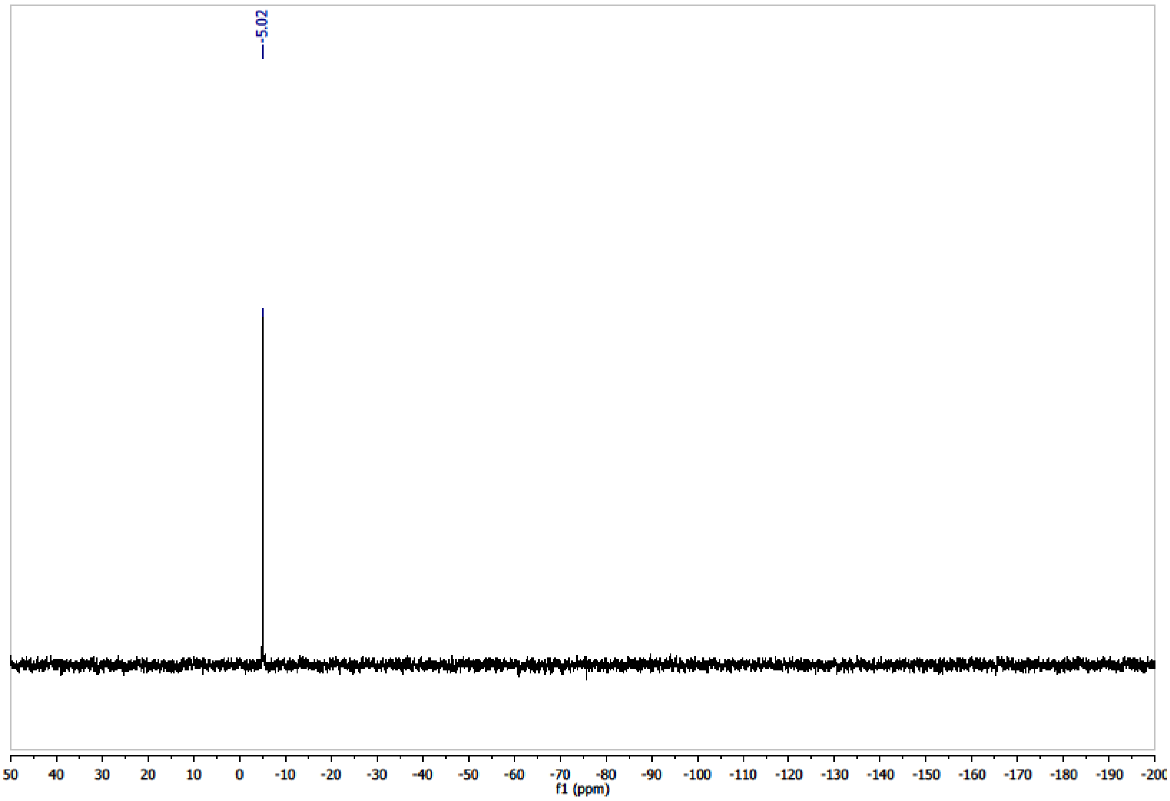

Figure S45. ^{29}Si INEPT NMR spectrum of **6a** in C_6D_6

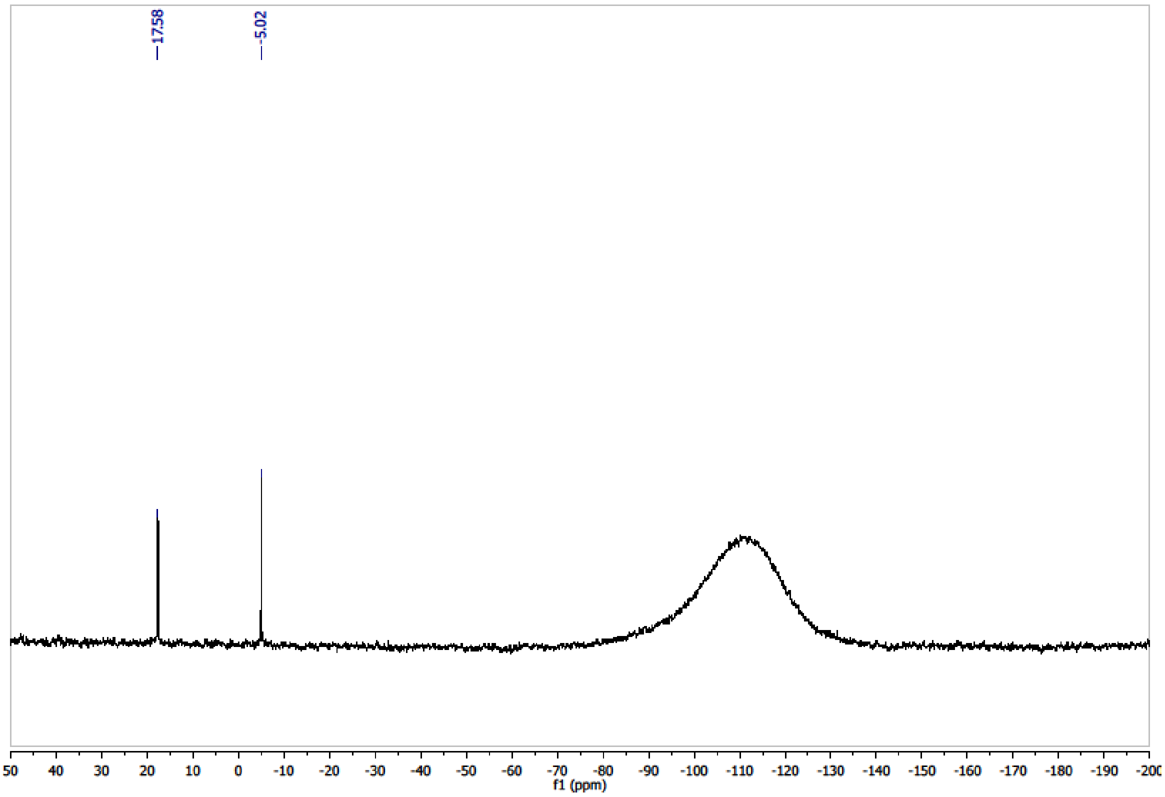

Figure S46. ^{29}Si inverse-gated NMR spectrum of **6a** in C_6D_6
